# Supplementary material for: Pseudo-RNA-Binding Domains Mediate RNA Structure Specificity in Upstream of N-Ras
Source: Cell Rep. 2020 Jul 21;32(3):107930. doi: 10.1016/j.celrep.2020.107930 (PMC7383231; doi:10.1016/j.celrep.2020.107930)
Supplement: Document S1. Figures S1–S5 and Tables S1–S3 and S5 [file mmc1.pdf]

Cell Reports, Volume 32

Supplemental Information

Pseudo-RNA-Binding Domains Mediate

RNA Structure Specificity in Upstream of N-Ras

Nele Merret Hollmann, Pravin Kumar Ankush Jagtap, Pawel Masiewicz, Tanit Guitart, Bernd Simon, Jan Provaznik, Frank Stein, Per Haberkant, Lara Jayne Sweetapple, Laura Villacorta, Dylan Mooijman, Vladimir Benes, Mikhail M. Savitski, Fátima Gebauer, and Janosch Hennig

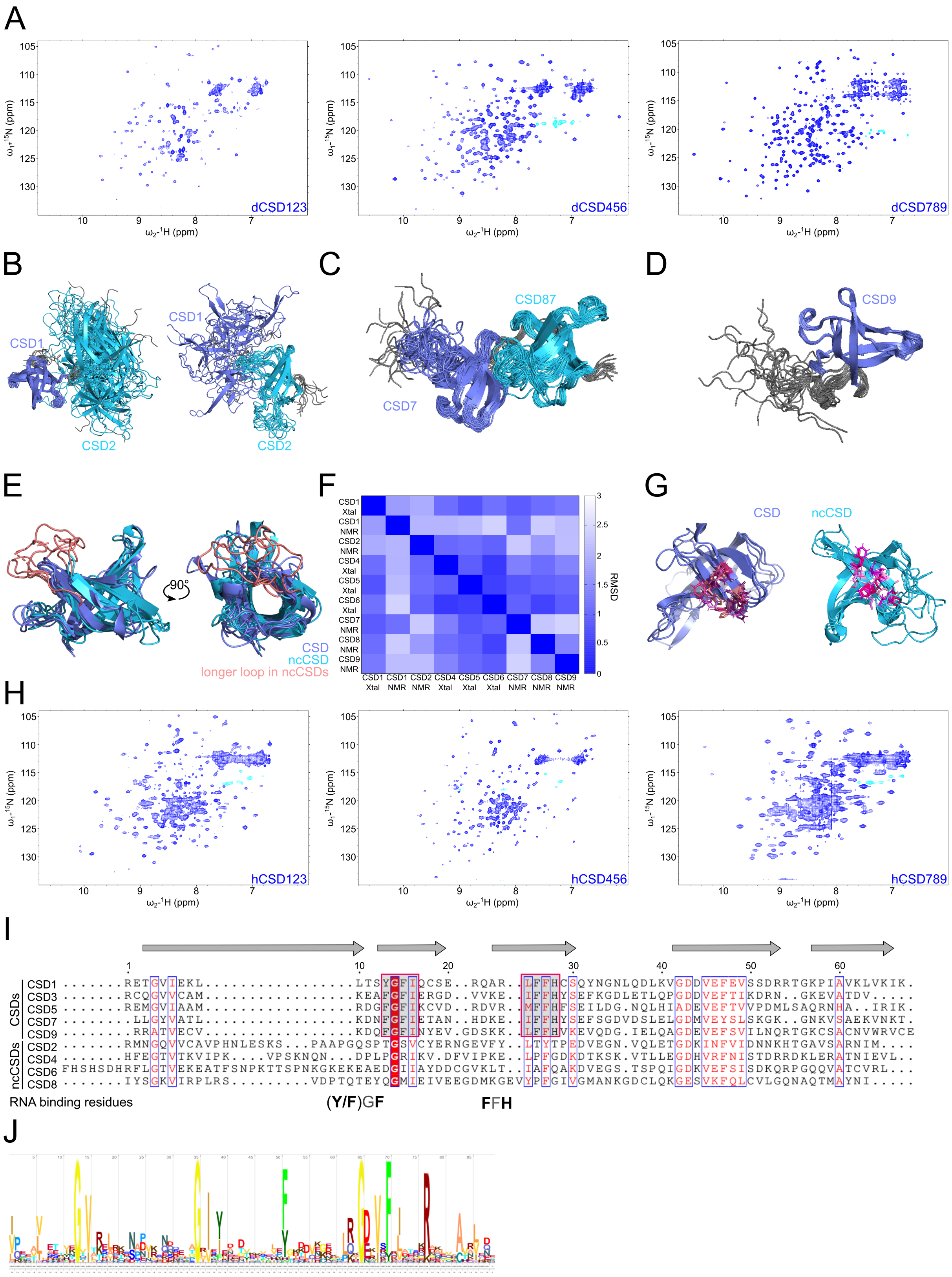

Supplementary Figure 1, Related to Figure 1: A: $^{1}H,^{15}N$-HSQC spectra of CSD123, CSD456 and CSD789 constructs. B: Ensemble of the 20 lowest energy NMR conformations of CSD12, superimposed on CSD1 (left) (residues: 197-237) and CSD2 (right) (residues: 269-274, 293-296 and 306-328) separately. C: Ensemble of the 20 lowest energy NMR conformations of CSD78 superimposed on secondary structure elements, excluding flexible loops and termini (residues 764-813, 824-834, 843-854, 867-873 and 879-920). D: Ensemble of the 20 lowest energy NMR conformations of CSD9 superimposed on the core CSD domain (residues 912-990). E: Superimposition of canonical CSDs 1, 5,

7 and 9 with ncCSDs 2, 4, 6 and 8. The additional loop that is present only in ncCSDs is highlighted in pink. F: Matrix of RMSD values from superimpositions of each single CSD of dUnr. For the comparison with CSD1 the crystal structure from PDB entry 4QQB was used. G: Superimposition of canonical CSDs 1, 5, 7 and 9 and ncCSDs 2,4,6 and 8 showing the RNA binding residues (F/Y-G-F and F-F-H) or equivalent residues pointing to the outside of the barrel (highlighted in shades of pink). H: $^{1}H,^{15}N$-HSQC spectra of CSD123, CSD456 and CSD789 of human Unr exhibit peak dispersion indicative of folded proteins and similar to dUnr. I: Sequence alignment of canonical and ncCSDs of human Unr. The same or similar residues between all domains are colored, similar residue regions between the canonical CSDs are highlighted by boxes, which align with the two RNA binding regions (Y/FGF and FFH). The alignment has been done using Emboss Needle (Madeira et al., 2019) and ESPript (Robert and Gouet, 2014) has been used for illustration. J: Presentation of the sequence logo that was used for the hidden markov model search.

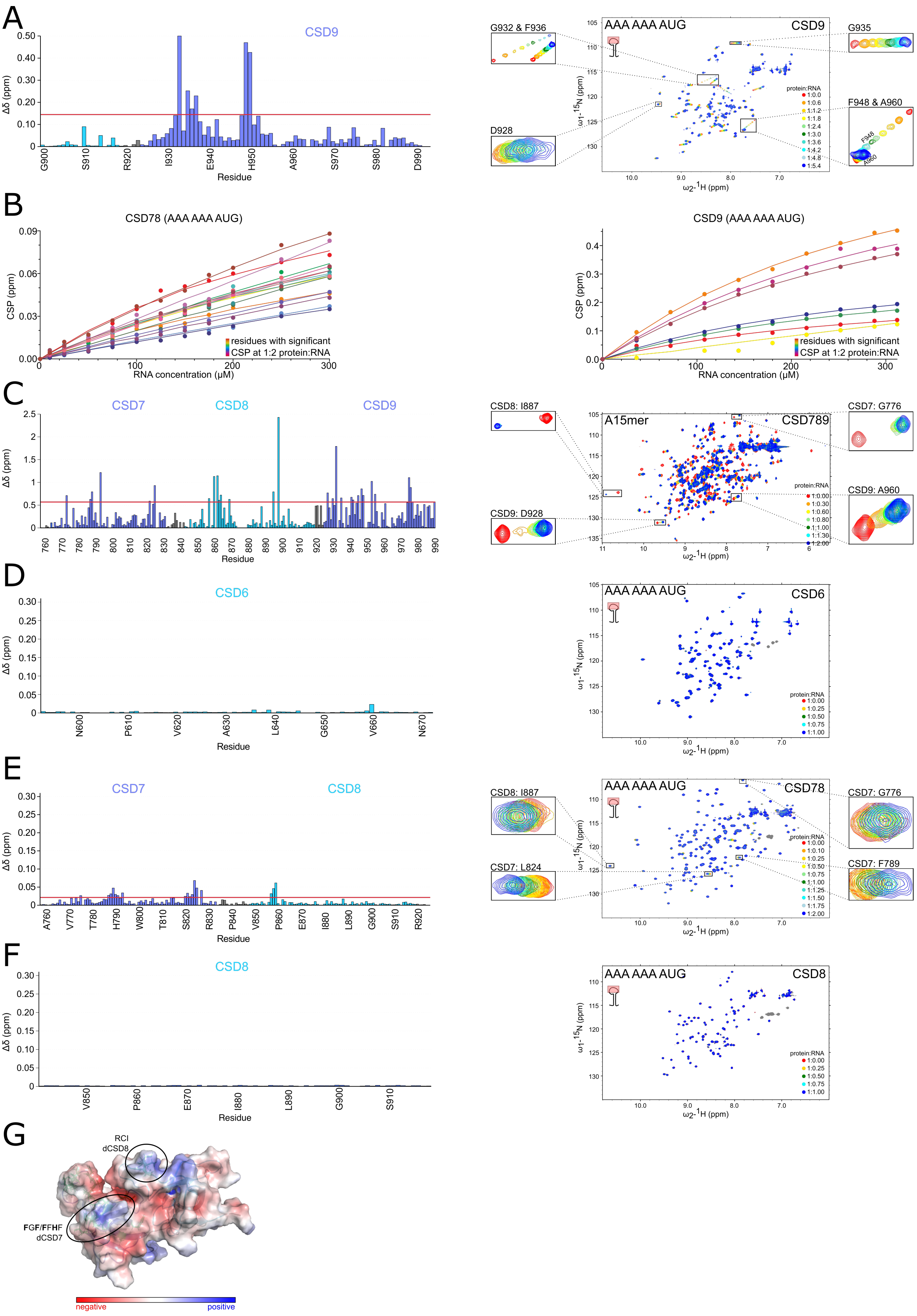

Supplementary Figure 2, Related to Figure 2: A: ^{1}H, ^{15}N-HSQC NMR titration of CSD9 (898-990) with a 9-mer RNA (AAA AAA AUG) zooming into regions, which show distinct shifts for some residues (right) and a histogram with chemical shift perturbation magnitude at end points per residue (left). B: The chemical shift perturbations and the corresponding fit for the different titration

concentrations for residues shifting significantly (CSP larger than the average plus standard deviation of all shifts) are shown. C: ^{1}H, ^{15}N-HSQC NMR titration of CSD789 (756-990) with an A15-mer RNA oligonucleotide zooming into regions, which show exemplary residues with distinct shifts (right) and a histogram with chemical shift perturbation magnitudes at the titration end points per residue (left). D: ^{1}H, ^{15}N-HSQC NMR titration of CSD6 (593-677) with a 9-mer RNA (AAA AAA AUG) (right) devoid of significant chemical shift perturbations, as shown also in the CSP plot (left). E: ^{1}H, ^{15}N-HSQC NMR titration of CSD78 (756-922) with a 9-mer RNA (AAA AAA AUG) zooming into regions, which show distinct shifts for some residues (right) and a histogram with chemical shift perturbations magnitude at end points per residue (left). F: ^{1}H, ^{15}N-HSQC NMR titration of CSD8 (841-922) with a 9-mer RNA (AAA AAA AUG) (right) devoid of significant chemical shift perturbations, as shown also in the CSP plot (left). G: Calculated surface potential for CSD78. Highlighted is the region of RNA binding residues of canonical CSD7 and the region of residues in ncCSD8, that show significant shifts after addition of RNA.

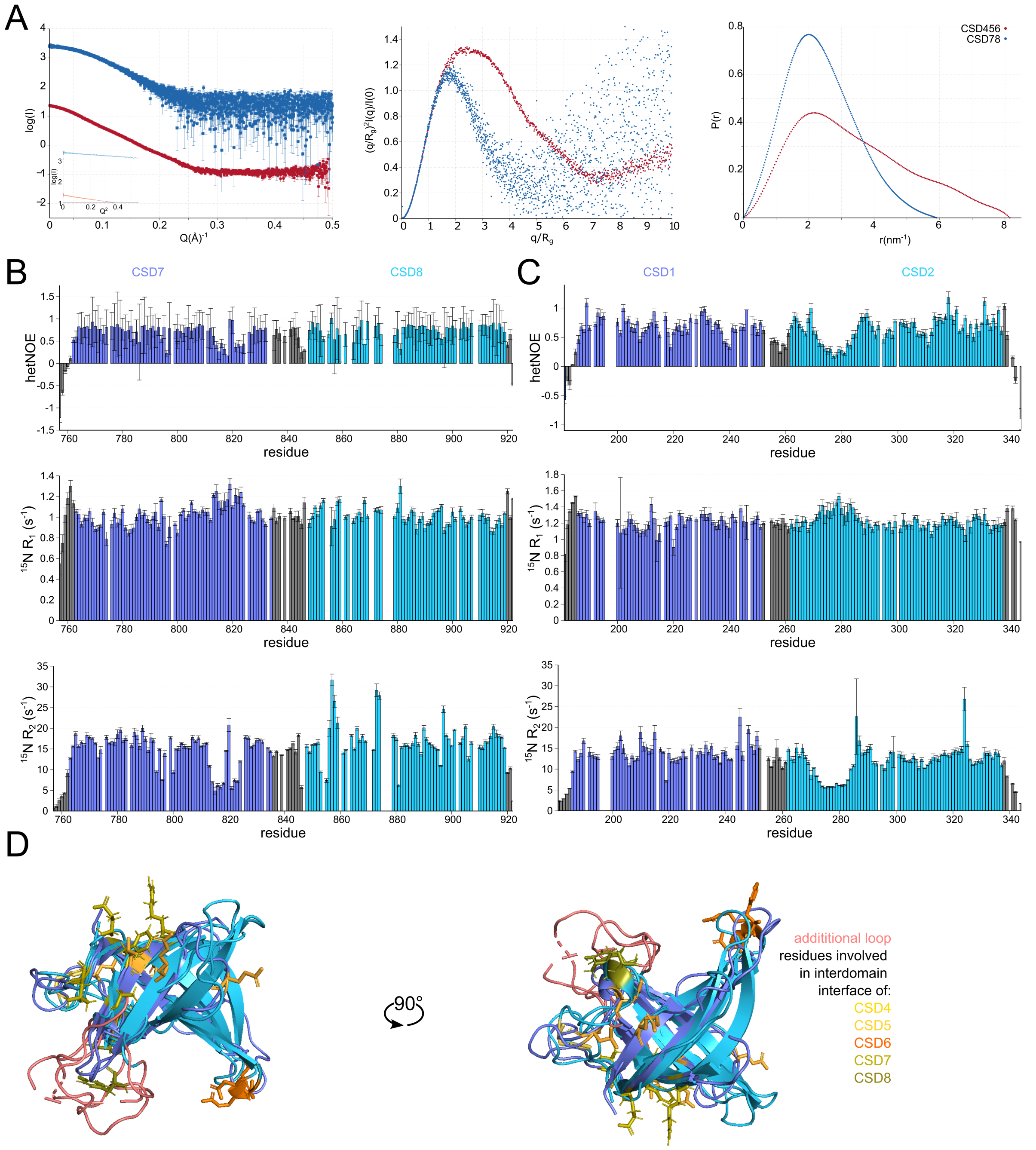

Supplementary Figure 3, Related to Figure 3: A left: $I(q)$ *versus* q as log-linear plots with the inset showing the Guinier fits for $qR_g < 1.3$ indicating good data quality and no aggregation for the curves of CSD456 (red) and CSD78 (blue). Middle: Dimensionless Kratky plots indicate that proteins (CSD456: red; CSD78: blue) are mostly structured with low flexibility. Right: $P(r)$ *versus* r profiles normalized to equal areas, showing the highest radius of gyration for CSD456 (red) and the lowest for CSD78 (blue). B and C: ^{15}N relaxation parameters of CSD78 (B) and CSD12 (C) showing the ^{1}H-^{15}N heteronuclear NOEs (hetNOE), the ^{15}N longitudinal relaxation rates (^{15}N R_1) and the ^{15}N transverse relaxation rates (^{15}N R_2). D: Superimposition of CSD5 and 7 and ncCSD4, 6 and 8, highlighting the additional loop that is present in the ncCSDs (pink) and the residues that are involved in formation of the interdomain interaction surface (shades from yellow to green).

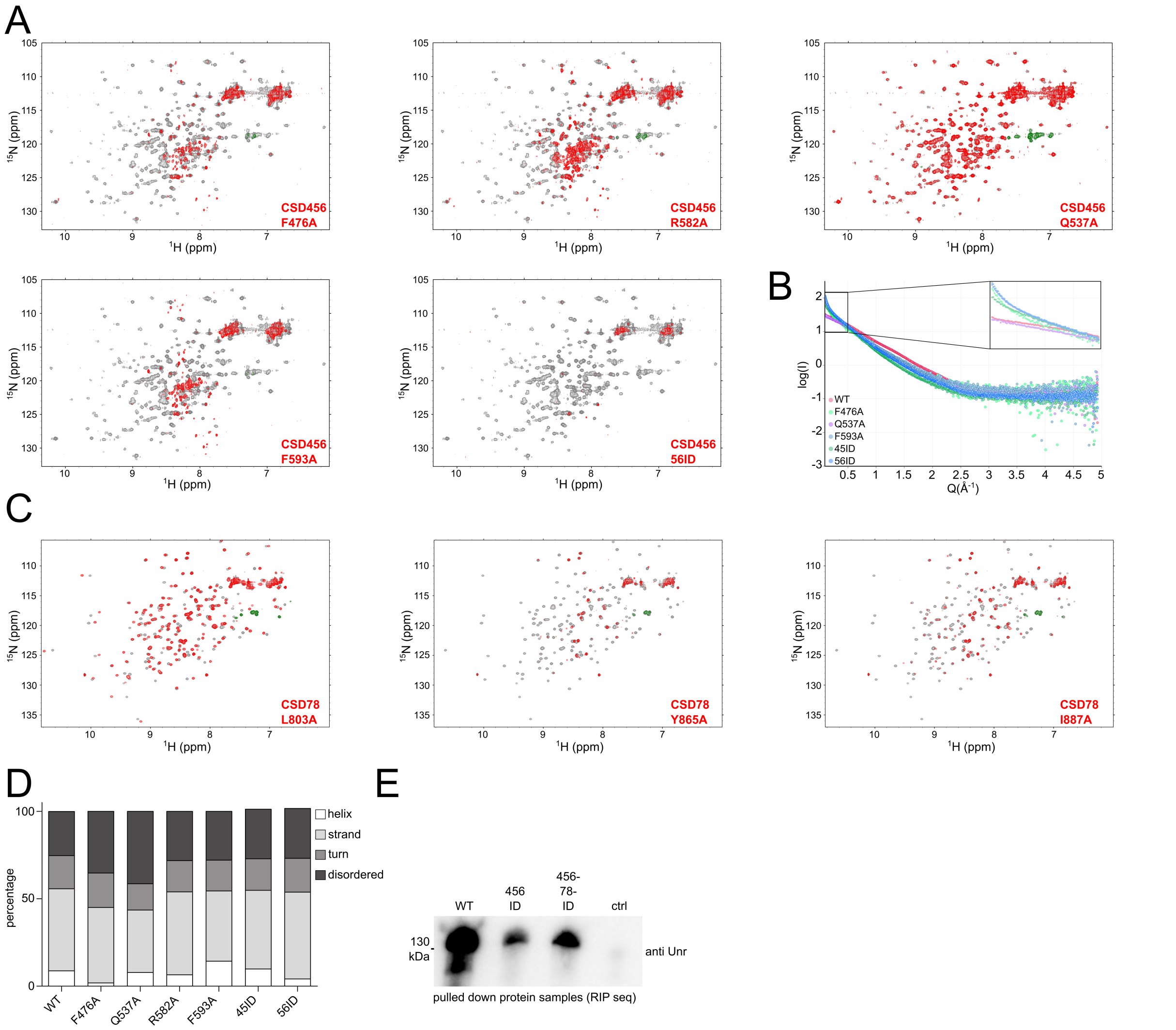

Supplementary Figure 4, Related to Figure 4: A: ^{1}H,^{15}N-HSQC spectra of CSD456 wild type (grey) overlaid with the spectra of CSD456 mutants (red) showing aggregation of most of the mutants compared to the wild type. The proteins had a concentration of 30 µM. B: SAXS curve of CSD456 wild type construct and the different mutants measured at the same concentration (1 mg/ml). Again, except for the Q538A mutant aggregation could be measured. C: ^{1}H,^{15}N-HSQCs of CSD78 wild type (grey) overlaid with the different spectra of CSD78 mutants (red). The L803A mutant shows CSPs compared to the wild type sample, whereas for the other two mutants aggregation could be observed The proteins had a concentration of 80 µM. D: Prediction of different secondary structure elements (helix, strand, turn and disordered) from CD curves of Unr full-length wt and different interdomain mutants, showing no difference between the wild type and mutant samples. E Western blot showing the efficiency of the Unr pull-down against the V5 tagged wild type and the two interdomain mutants of the samples (456ID, 456-78-ID) used for RIP seq. No protein was detected in the control sample, which was transfected with an empty vector. Unr antibody against an N-terminal part of the protein was used.

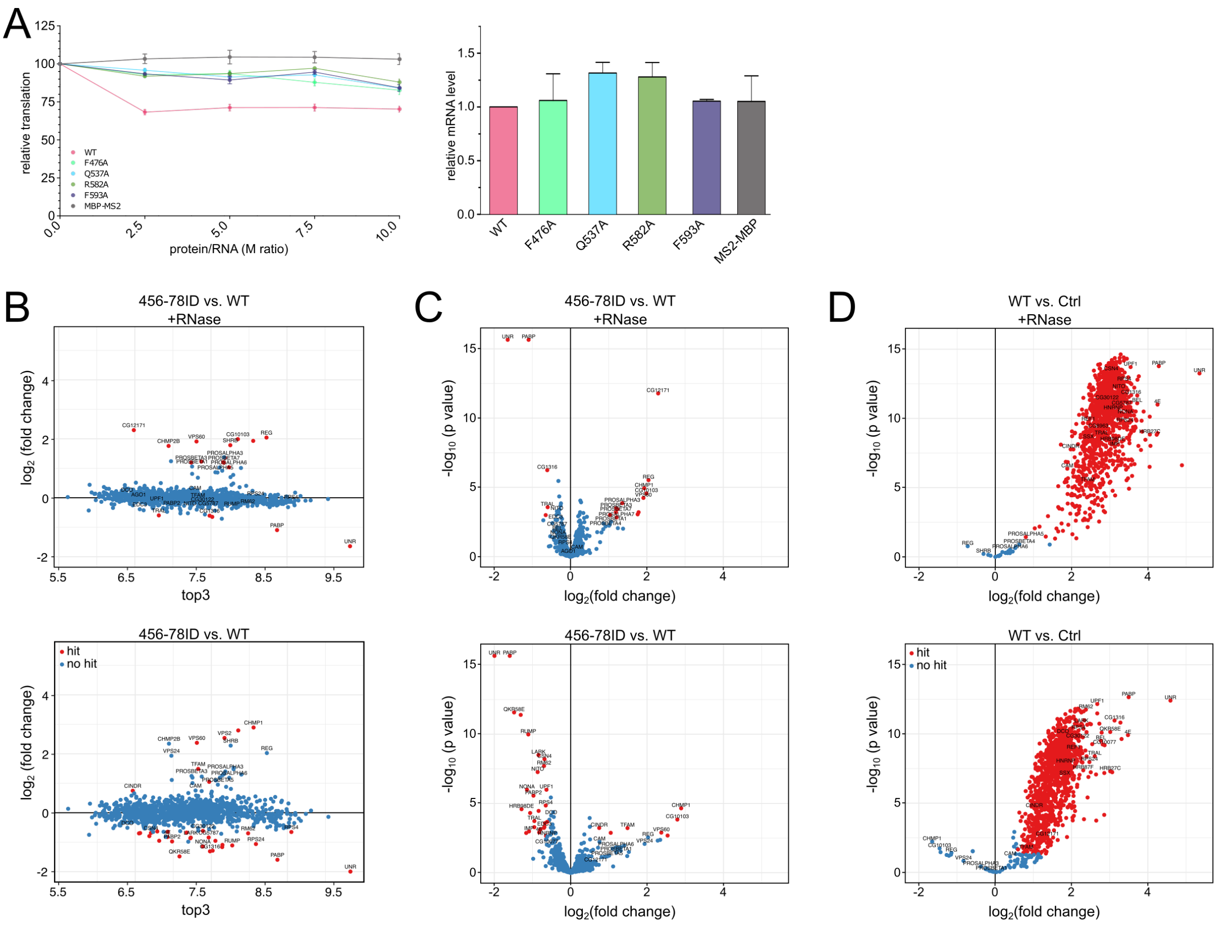

Supplementary Figure 5, Related to Figure 5: A left: Relative *in vitro* translation of the Firefly reporter gene over the internal control Renilla after adding different amounts of single dUnr mutants compared to wild type dUnr. N=3. The wild type protein shows a higher translation repression rate than the mutants. The control (MBP-MS2) doesn't show any repression. Right: Relative mRNA levels of the ratio of Renilla and Firefly after in vitro translation experiments, measured by qPCR. N=3 from 2.5 excess of protein over RNA, indicating, that the observed difference in signal is not due to different mRNA levels.. B: Scatter plot showing the top3 value (average abundance of a protein in the mass spectrometry run) versus the log2(fold change) of interdomain mutant vs WT for RNase treated (top) and non-treated (bottom) conditions. N=3. C/D: Volcano plots showing the difference of protein targets of the IP-MS between the WT sample and the 456-78 interdomain mutant (C) or the control (D) with (upper graph) and without RNase treatment (lower graph). The $\log_2$(fold change) is plotted against the negative $\log_{10}$(p value). Differentially regulated proteins are colored in red. D: Due to the low background level of the control sample, almost all proteins were classified as differentially regulated. N=3

Table S1, Related to Figure 1. List of soluble and unsoluble protein constructs tested for *Drosophila* Unr.

boundaries	old nomenclature	new nomenclature	tag
2-130	N-temrinus + q-rich	N-temrinus + q-rich	His
2-183	N-temrinus + q-rich	N-temrinus + q-rich	His
11-130	N-temrinus + q-rich	N-temrinus + q-rich	His
11-183	N-temrinus + q-rich	N-temrinus + q-rich	His
176-414	CSD1/2	CSD1/2/3	His
176-669	CSD123	CSD1-6	His
176-677	CSD123	CSD1-6	His
186-252	CSD1	CSD1	His-Trx
186-324	CSD1	CSD12	His
186-339	CSD1	CSD12	His
186-341	CSD1	CSD12	His
186-344	CSD1	CSD12	His
186-414	CSD1/2	CSD1/2/3	His
186-990	CSD1-5	CSD1-9	His
345-515	CSD2	CSD34	His
345-586	CSD35	CSD345	His
422-515		CSD4	His
422-586	CSD3	CSD4/5	His
422-677	CSD3	CSD456	His
422-853	CSD3/4	CSD4-7	His
428-990	CSD345	CSD4-9	His
516-581	CSD3	CSD5	His
516-669	CSD3	CSD5/6	His
593-677		CSD6	His
593-757		CSD6 + q-rich	
593-922	CSD4	CSD678	His
678-922	CSD4	q-rich + CSD78	
757--922	CSD4	CSD78	His
757-853	CSD4	CSD7	His
757-856	CSD4	CSD7	His
757-991	CSD4/5	CSD7/8/9	His
856-991	CSD5	CSD8/9	His
899-991	CSD5	CSD9	His
2-52	N-terminus	N-terminus	His
2-61	N-terminus	N-terminus	His
11-52	N-terminus	N-terminus	His
11-61	N-terminus	N-terminus	His
47-130	N-temrinus + q-rich	N-temrinus + q-rich	His
47-183	N-temrinus + q-rich	N-temrinus + q-rich	His
54-130	q-rich	q-rich	His

- soluble - not used in publication
- soluble - used in publication
- not soluble

54-183	q-rich	q-rich	His
62-130	q-rich	q-rich	His
62-183	q-rich	q-rich	His
186-1017	CSD1-5	CSD1-9	His
186-669	CSD123	CSD1-6	His
186-677	CSD123	CSD1-6	His
285-424	CSD2	CSD2/3	His
286-502	CSD2	CSD34	His
292-414	CSD2	CSD23	His
293-581	CSD35	CSD345	His
293-586	CSD35	CSD345	His
293-642	CSD35	CSD345	His
293-669	CSD35	CSD3456	His
293-677	CSD35	CSD3456	His
298-502	CSD2	CSD34	His
298-515	CSD2	CSD34	His
345-414	CSD2	CSD3	His
345-414	CSD2	CSD3	His-MBP
345-502	CSD2	CSD34	His
345-581	CSD35	CSD345	His
345-643	CSD35	CSD345	His
345-669	CSD35	CSD3456	His
345-677	CSD35	CSD3456	His
422-1017	CSD345	CSD4-9	His
422-502		CSD4	His
422-581	CSD3	CSD4/5	His
422-642	CSD3	CSD45	His
422-832	CSD34	CSD4-7	His
422-835	CSD34	CSD4-7	His
422-856	CSD34	CSD4-7	His
422-875	CSD34	CSD4-7 (8half)	His
422-910	CSD34	CSD4-7 (8half)	His
422-910	CSD34	CSD4-8	His
422-990	CSD345	CSD4-9	His
428-1017	CSD345	CSD4-9	His
428-502		CSD4	His
428-515		CSD4	His
428-581	CSD3	CSD4/5	His
428-596	CSD3	CSD4/5	His
446-642	CSD3	CSD56	His
446-832	CSD34	CSD4-7	His
446-835	CSD34	CSD4-7	His
446-853	CSD34	CSD4-7	His
446-856	CSD34	CSD4-7	His

446-875	CSD34	CSD4-7 (8half)	His
446-910	CSD34	CSD4-8	His
516-586	CSD3	CSD5	His
516-586	CSD3	CSD5	His-MBP
516-586	CSD3	CSD5	His
516-677	CSD3	CSD5/6	His
516-832	CSD34	CSD5-7	His
516-835	CSD34	CSD5-7	His
516-853	CSD34	CSD5-7	His
516-856	CSD34	CSD5-7	His
516-875	CSD34	CSD5-7 (8half)	His
516-910	CSD34	CSD5-8	His
593-669		CSD6	His
678-757	q-rich	q-rich	His
678-703	q-rich	q-rich	His
703-757	q-rich	q-rich	His
757-1017	CSD4/5	CSD7/8/9	His
757-875	CSD4	CSD7	His
757-890	CSD4	CSD78	His
757-900	CSD4	CSD78	His
757-911	CSD4	CSD78	His
763-832	CSD4	CSD7	His
763-832	CSD4	CSD7	His-MBP
763-910	CSD4	CSD7	His
856-1017	CSD5	CSD8/9	His
899-1017	CSD5	CSD9	His
910-1017	CSD5	CSD9	His
911-990	CSD5	CSD9	His
923-990	CSD5	CSD9	His
345-414	CSD2	CSD3	His-Trx
516-586	CSD3	CSD5	His-Trx
763-832	CSD4	CSD7	His-Trx
923-990	CSD5	CSD9	His-Trx

Table S2, Related to Figure 1. Statistics of the NMR structure calculation of *Drosophila* Unr CSD12 bound to SL6 apical RNA, CSD78 and CSD9 and data collection and refinement statistics of the crystal structure of *Drosophila* Unr CSD456. Statistics for the highest-resolution shell are shown in parentheses.

NMR structure statistics			
	**dCSD12+SL6 apical**	**dCSD78**	**dCSD9**
	**Experimental restraints**		
Total NOEs	3911	4530	3712
distance restraints	1788	2323	1831
Short range (|i – j| ≤ 1)	1005	1242	544
Medium range (|i – j| < 5)	124	211	283
Long range (|i – j| > 5)	659	870	1004
Dihedral restraints (ϕ/ψ)	224	167	93
	**Structural Quality**		
Coordinate precision (Å)			
Backbone (N, Cα, C′)	0.40 (CSD1), 0.53 (CSD2) (a)	0.39 (CSD7), 0.39 (CSD8) (b)	0.38 (c)
Heavy atoms	0.99 (CSD1), 1.05 (CSD2) (a)	0.84 (CSD7), 0.86 (CSD8) (b)	0.80 (c)
Restraint RMSD			
Distance restraints, Å	0.02 +/- 0.0019	0.0196 +/- 0.002	0.0253 +/- 0.003
Dihedral restraints, °	0.666 +/- 0.104	1.437 +/- 0.101	0.565 +/- 0.105
Deviation from idealized geometry			
Bond lengths, Å	0.0038 +/- 0.00008	0.0036 +/- 0.00009	0.00434 +/- 0.00013
Bond angles, °	0.504 +/- 0.01	0.491 +/- 0.011	0.542 +/- 0.022
	**Whatcheck analysis**		

First generation packing	-3.271 +/- 0.228	-2.751 +/- 0.161	-1.912 +/- 0.191
Second-generation packing	-2.491 +/- 0.274	-2.402 +/- 0.239	-1.560 +/- 0.284
Ramachandran plot appearance	-3.806 +/- 0.298	-3.843 +/- 0.329	-4.442 +/- 0.480
χ-1/χ-2 rotamer normality	-3.806 +/- 0.532	-3.915 +/- 0.473	-5.416 +/- 0.431
Backbone confirmation	-1.261 +/- 0.534	-2.329 +/- 0.537	-1.326 +/- 0.376
	**Ramachandran analysis, %**		
Favored regions	80.9	76.7	80.5
Allowed regions	16.7	20.9	18.9
Generously allowed	1.9	1.7	0.6
Disallowed	0.4	0.7	0.0

a	For residues using 197-237 (CSD1) and 269-274, 293-296 and 306-328 (CSD2)
b	For residues using 764-813 and 824-834 (CSD7) and 843-854, 867-873 and 879-920 for (CSD8)
c	For residues using 912-990

**Crystallography statistics CSD456**	
Wavelength	1.005 Å
Resolution range	77.84 - 2.02 (2.092 - 2.02)
Space group	P 62
Unit cell	89.88 89.88 58.75 90 90 120
Total reflections	113928 (6419)
Unique reflections	34128 (2534)
Multiplicity	3.3 (2.5)
Completeness (%)	98.00 (87.3)
Mean I/sigma(I)	7.8 (1.0)
Wilson B-factor	39.76
R-merge	0.073 (0.78)
R-meas	0.087 (0.95)
R-pim	0.046 (0.54)
CC1/2	0.99 (0.57)
Reflections used in refinement	32428 (2409)
Reflections used for R-free	1698 (124)

R-work	0.22 (0.32)
R-free	0.25 (0.32)
Number of non-hydrogen atoms	2062
macromolecules	1842
ligands	7
solvent	213
Protein residues	238
RMS(bonds)	0.002
RMS(angles)	0.38
Ramachandran favored (%)	96.93
Ramachandran allowed (%)	2.63
Ramachandran outliers (%)	0.44
Rotamer outliers (%)	0.00
Average B-factor	47.73
macromolecules	47.83
ligands	51.27
solvent	46.76

Table S3, Related to Figure 3. Statistics of the SAXS data and processing statistics of *Drosophila* Unr CSD456 and CSD78.

	**dCSD456**	**dCSD78**
(a) Sample Details		
Organism	*E. coli* BL2 (DE3)	*E. coli* BL2 (DE3)
Source	*this work*	*this work*
Uniprot sequence ID	Q9VSK3	Q9VSK3
Description	UNR E422-H677, with TEV-cleaved N-terminal His6-tag	UNR A756-K922, with TEV-cleaved N-terminal His6-tag
Molecular mass M from chemical composition (Da)	29.532	18.597
loading concentration (mg/ml)	1.16 and 4.65 mg/ml	0.8 mg/ml
injection volume (ul)	30	30
concentration (uM)	40/160	45
Solvent composition and source	20 mM Hepes/NaOH pH 7.5, 150 mM NaCl and 1 mM DTT	
(b) SAS data collection parameter		
Source and instrument	Grenoble ESRF BM29 with Dectris Pilatus 1M	Hamburg PETRA-III P12 with Dectris Pilatus 6M (REF)
Wavelength (A)	0.9919	1.24
Sample-detector distance (m)	2,867	3.0
q-measurement range (nm-1)	0.0355-4.9391	0.0224-7.3176
Radiation daage monitoring	frame-by-frame comparison	
Exposure time (s) & number	1.0x10	0.195x20
Sample configuration	sample chamger with flow through capillary measurement	
Sample temperature (℃)	20	25
(c) Software employed for SAS data reduction, analysis and interprtation		
SAXS data processing	I(q) vs. q using Bsx cube, solvent subtraction and curve merging using PRIMUSqt from ATSAS (Franke et al., 2017)	
Basic analyses: Guinier, P(r), Vp	PRIMUSqt from ATSAS 2.7.1 (Franke et al., 2017)	

Atomic structure modelling	CRYSOL 2.8.2 from PRIMUSqt in ATSAS 2.8 (Svergun et al., 1995)	
Molecular graphics	--	--
(d) Structural parameters		
	Guinier analysis	
I(0) (raw)	22.8+/-0.06	25.4+/-0.05
R_g (Å)	26.7+/-0.1	18.1+/-0.01
qR_g max (q_{min} = 0.0066 $Å^{-1}$)	1.28	1.3
Coefficient of correlation, R^2	0.97	0.74
	P(r) Analysis from AUTOGNOM	
I(0) (cm-1)	22.8	25.43
R_g (Å)	22.61	18.2
d_{max} (Å)	81.5	59.1
q range ($Å^{-1}$)	0.102-3.00	0.089-4.427
χ^2 (total estimate from *GNOM*)	0.67	0.89
Porod volume ($Å^{-3}$) (ratio V_P/calculated *M)*	37490	27610
(f) Atomistic modelling		
Method	CRYSOL (Svergun et al., 1995)	
Crystal stucture	UNR CSD456 (6Y6E)	UNR CSD78 (6Y4H)
CRYSOL	Constant subtraction allowed	
χ^2	1,026	1,218
Predicted R_g (Å)	26.22	18.09
Vol (Å), Ra (Å), Dro (e $Å^{-3}$)	25504, 1.4, 0.07	17716, 1.8, 0.000

Table S5, Related to STAR Methods. Sequences for DNA oligonucleotides.

REAGENT or RESOURCE	SOURCE	IDENTIFIER
RNAi targeting Unr forward primer: TAATACGACTCACTATAGGGATTGCTGAAGAAGTTGTATAAGCAA	Militti et al., 2014	N/A
RNAi targeting Unr reverse primer: TAATACGACTCACTATAGGGACACTTGGATTTCAATTTCGTTTTGC	Militti et al., 2014	N/A
RNAi targeting GFP forward primer: TAATACGACTCACTATAGGGCCTGAAGTTCATCTGCACCA	Militti et al., 2014	N/A
RNAi targeting GFP reverse primer: TAATACGACTCACTATAGGGTGAAATTTGTGATGCTATTGCTTT	Militti et al., 2014	N/A
forward qPCR primer against firefly luciferase used in *in vitro* translation assay: TTGTTTCCAAAAAGGGGTTG	Graindorge et al., 2013	N/A
reverse qPCR primer against firefly luciferase used in *in vitro* translation assay: CATCGACTGAAATCCCTGGT	Graindorge et al., 2013	N/A
forward qPCR primer against renilla luciferase used in *in vitro* translation assay: TATTGCTTTGATCTTATCTTGATGC	This paper	N/A
reverse qPCR primer against renilla luciferase used in *in vitro* translation assay: ACAAATATCTTACTGCATGGTTTG	This paper	N/A
forward qPCR primer against β-galactosidase used in cell assay: AACGTCGTGACTGGGAAAAC	Graindorge et al., 2013	N/A
reverse qPCR primer against β-galactosidase used in cell assay: GGCCTCTTCGCTATTACGC	Graindorge et al., 2013	N/A
forward qPCR primer against renilla luciferase used in cell assay: ACAAGTACCTCACCGCTTGG	Graindorge et al., 2013	N/A
reverse qPCR primer renilla luciferase used in cell assay: GACACTCTCAGCATGGACGA	Graindorge et al., 2013	N/A
