# Supplementary material for: Pseudo-RNA-Binding Domains Mediate RNA Structure Specificity in Upstream of N-Ras
Source: Cell Rep. 2020 Jul 21;32(3):107930. doi: 10.1016/j.celrep.2020.107930 (PMC7383231; doi:10.1016/j.celrep.2020.107930)
Supplement: Document S2. Article plus Supplemental Information [file mmc3.pdf]

Cell Reports

Article

Pseudo-RNA-Binding Domains Mediate RNA Structure Specificity in Upstream of N-Ras

Graphical Abstract

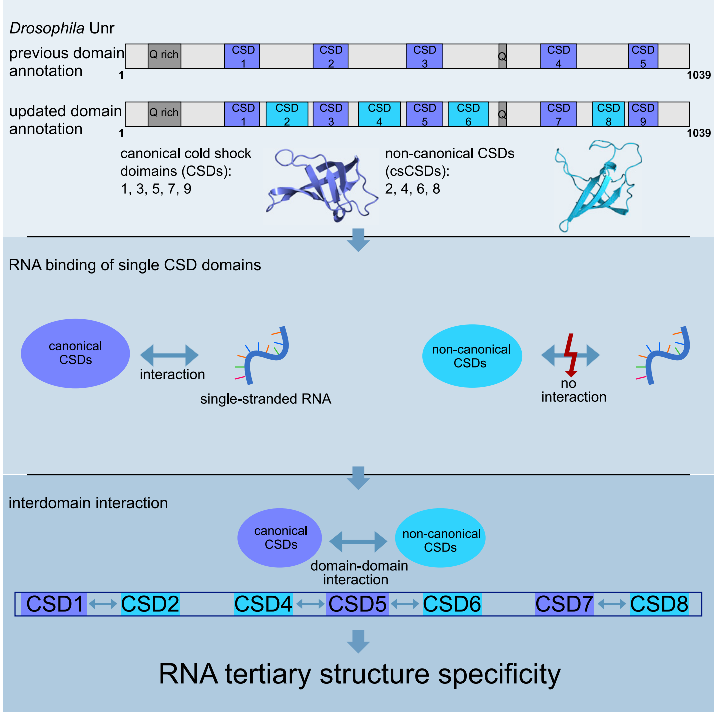

Highlights

- Discovery of non-canonical cold-shock domains
- Non-canonical cold-shock domains do not bind RNA independently
- Interdomain contacts mediate RNA structure specificity and impact translation
- Determination of an Unr-dependent ribonucleoprotein (RNP) interactome

Authors

Nele Merret Hollmann, Pravin Kumar Ankush Jagtap, Pawel Masiewicz, ..., Mikhail M. Savitski, Fátima Gebauer, Janosch Hennig

Correspondence

janosch.hennig@embl.de

In Brief

Hollmann et al. show how non-RNA-binding domains within *Drosophila* Unr, an RNA-binding protein, contribute to its RNA target specificity. The selectivity is mediated by interdomain contacts to the RNA-binding cold-shock domains, which restrict the protein shape.

Hollmann et al., 2020, Cell Reports *32*, 107930
July 21, 2020 © 2020 The Authors.
https://doi.org/10.1016/j.celrep.2020.107930

CellPress

Cell Reports

CellPress
OPEN ACCESS

Article

Pseudo-RNA-Binding Domains Mediate RNA Structure Specificity in Upstream of N-Ras

Nele Merret Hollmann,[1,2] Pravin Kumar Ankush Jagtap,[1] Pawel Masiewicz,[1] Tanit Guitart,[3] Bernd Simon,[1] Jan Provaznik,[5] Frank Stein,[6] Per Haberkant,[6] Lara Jayne Sweetapple,[1] Laura Villacorta,[5] Dylan Mooijman,[7] Vladimir Benes,[5] Mikhail M. Savitski,[6,8] Fátima Gebauer,[3,4] and Janosch Hennig[1,9,*]

[1]Structural and Computational Biology Unit, EMBL Heidelberg, Meyerhofstraße 1, 69117 Heidelberg, Germany
[2]Collaboration for Joint PhD Degree between EMBL and Heidelberg University, Faculty of Biosciences, Heidelberg, Germany
[3]Gene Regulation, Stem Cells and Cancer Programme, Centre for Genomic Regulation (CRG), The Barcelona Institute of Science and Technology, 08003 Barcelona, Spain
[4]Universitat Pompeu Fabra (UPF), 08003 Barcelona, Spain
[5]Genomics Core Facility, EMBL Heidelberg, Meyerhofstraße 1, 69117 Heidelberg, Germany
[6]Proteomics Core Facility, EMBL Heidelberg, Meyerhofstraße 1, 69117 Heidelberg, Germany
[7]Developmental Biology Unit, EMBL Heidelberg, Meyerhofstraße 1, 69117 Heidelberg, Germany
[8]Genome Biology Unit, EMBL Heidelberg, Meyerhofstraße 1, 69117 Heidelberg, Germany
[9]Lead Contact
*Correspondence: janosch.hennig@embl.de
https://doi.org/10.1016/j.celrep.2020.107930

SUMMARY

RNA-binding proteins (RBPs) commonly feature multiple RNA-binding domains (RBDs), which provide these proteins with a modular architecture. Accumulating evidence supports that RBP architectural modularity and adaptability define the specificity of their interactions with RNA. However, how multiple RBDs recognize their cognate single-stranded RNA (ssRNA) sequences in concert remains poorly understood. Here, we use Upstream of N-Ras (Unr) as a model system to address this question. Although reported to contain five ssRNA-binding cold-shock domains (CSDs), we demonstrate that Unr includes an additional four CSDs that do not bind RNA (pseudo-RBDs) but are involved in mediating RNA tertiary structure specificity by reducing the conformational heterogeneity of Unr. Disrupting the interactions between canonical and non-canonical CSDs impacts RNA binding, Unr-mediated translation regulation, and the Unr-dependent RNA interactome. Taken together, our studies reveal a new paradigm in protein-RNA recognition, where interactions between RBDs and pseudo-RBDs select RNA tertiary structures, influence RNP assembly, and define target specificity.

INTRODUCTION

Current estimates indicate that approximately 10% of the entire human genome codes for RNA-binding proteins (RBPs) (Hentze et al., 2018). RBPs interact with coding and non-coding RNAs to regulate gene expression at all levels, including transcription, splicing, polyadenylation, localization, stabilization, degradation, as well as protein synthesis via their roles in translation (Cech and Steitz, 2014; Gerstberger et al., 2014; Hentze et al., 2018; Singh et al., 2015). Consequently, dysregulation or mutation of RBPs impacts cell viability and function, and has been linked to diseases, such as cancer (Pereira et al., 2017) and neurodegeneration (Conlon and Manley, 2017).

RBPs employ RNA-binding domains (RBDs) to engage their target RNAs. The majority of RBPs contain more than one RBD, resulting in a large combinatorial variety of different domain classes, and diversity of architectures and modes of target RNA sequence binding. In many cases these domains are separated by flexible linker regions (Afroz et al., 2015; Gerstberger et al., 2014). The modular architecture of RBPs and the exact spatial arrangement of the RBDs are thought to be critically important for the specificity of target RNA binding. In general, most RBDs can accommodate only three to five contiguous RNA bases, which cannot be used to discriminate target from non-target RNAs within the transcriptome in the complex cellular environment. Therefore, the composition and architecture of multiple RBDs within one or multiple RBPs is believed to be required to endow specificity (Auweter et al., 2006; Hennig and Sattler, 2015; Hennig et al., 2014a, 2014b). Over the years, there have been a number of efforts to examine structural features that dictate RBP/RNA-binding specificity (Cléry and Allain, 2011). These efforts have increased our understanding of how single RBDs engage their target sequences, and in some cases offered insights into the role of multi-domain arrangements in the recognition process. Additionally, efforts to map the RBP/RNA-binding landscape *in vitro* and *in vivo* have now highlighted that protein features beyond the domain boundaries of RBDs play an important role in directing specificity (Sasse et al., 2018). Moreover, we now appreciate that target RNA recognition employs complex binding modes that depend on the target itself, such

This is an open access article under the CC BY license (http://creativecommons.org/licenses/by/4.0/).

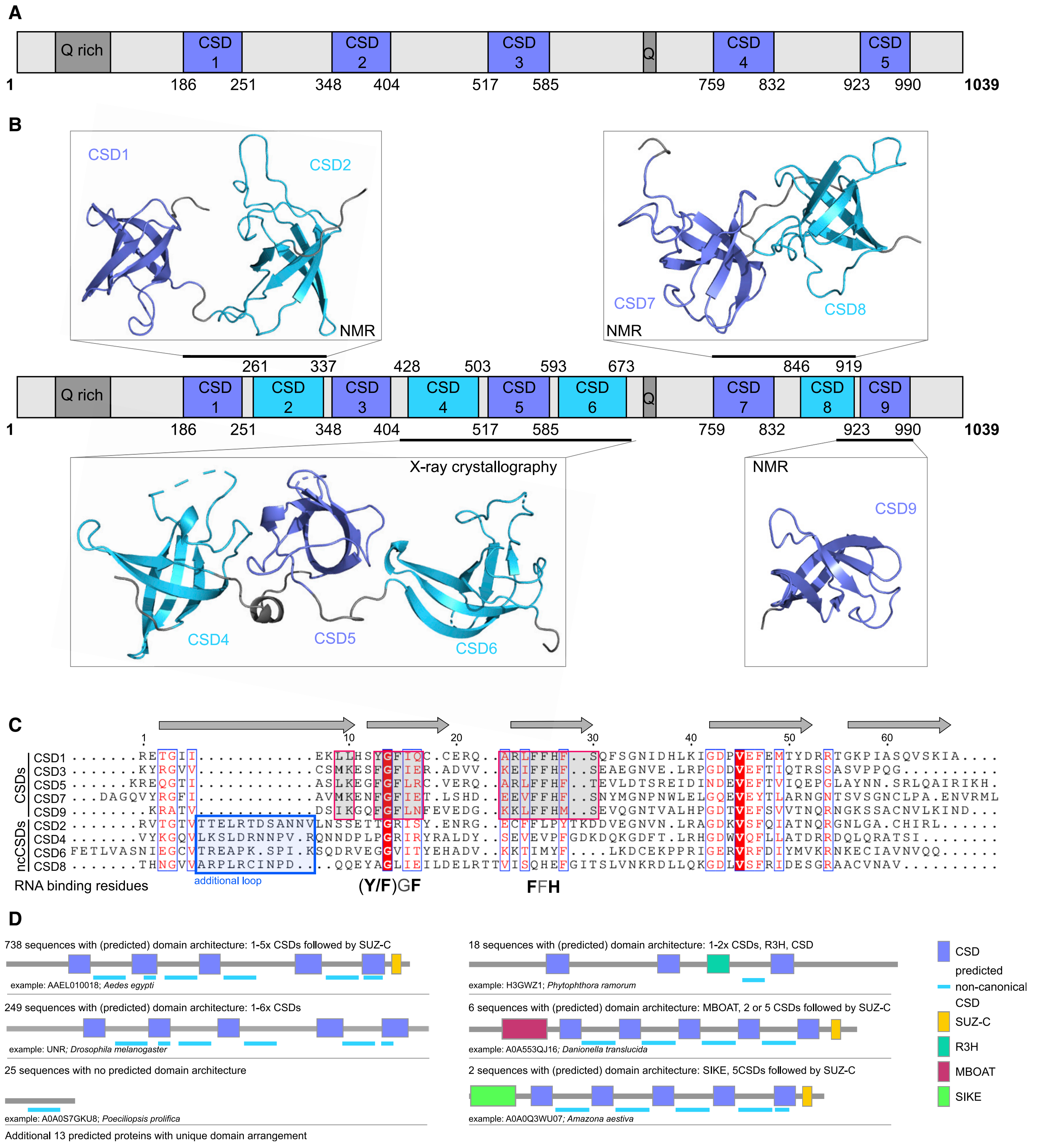

Figure 1. Revised Domain Assignment in *Drosophila* Unr (dUnr)

(A) Hitherto domain arrangement scheme of *Drosophila* Unr that shows the distribution of the five canonical CSDs that have been previously annotated (1, 2, 3, 4, and 5).

(B) Revised domain arrangement scheme (middle) that shows the distribution of previous CSDs (now numbered 1, 3, 5, 7, and 9; purple) and the four non-canonical CSDs (ncCSDs) we discovered in this work (2, 4, 6, and 8; cyan). (Clockwise) NMR solution structures of dUnrCSD12 (aa 179–344; PDB: 6Y6M), CSD78 (aa 756–922; PDB: 6Y4H), CSD9 (aa 899–989; PDB: 6Y96), and a crystal structure of CSD456 (aa 424–677; PDB: 6Y6E), all determined in this study, are arranged around the revised domain arrangement scheme; note that only a single representative NMR assemble structure is shown per construct for clarity, and ensembles are provided in Figure S1.

(legend continued on next page)

as secondary and tertiary structures of the RNA, presence and distribution of bipartite motifs, and nature of flanking nucleotides (Dominguez et al., 2018). However, a detailed mechanistic view of how multiple RBDs recognize their cognate single-stranded RNA (ssRNA) sequences in concert is largely missing.

The main obstacle to structural characterization of RBP/ssRNA interactions stems from technical difficulties in expressing and purifying multi-domain and full-length constructs of RBPs, as well as generating high-quality samples for crystallization or cryo-electron microscopy (cryo-EM) analysis. Some notable exceptions in this area have been the structures of large ribonucleoprotein (RNP) machineries, such as ribosomes (Bieri et al., 2018; Voorhees and Ramakrishnan, 2013) and spliceosomes (Wan et al., 2019; Wilkinson et al., 2018). In those cases, years of breakthrough work toward defining critical components that must engage in order to generate a stable assembly amenable to isolation and structural characterization played a critical role. Therefore, as seen in these examples, defining the interactome can be a powerful strategy toward enabling detailed structural and mechanistic studies.

However, the large majority of RBPs, especially those interacting with long non-coding RNAs (lncRNAs), are proving to be exceptionally recalcitrant to structural characterization. As of the time of this report, the number of lncRNA-related structural information, including RBDs and RBPs, known to engage lncRNA, reported in the Protein Data Bank, has been limited (less than 20 RNA and protein/RNA complexes out of more than 160,000 structures reported). Here, we address this challenge by presenting the results of our comprehensive and systematic investigation of Upstream of N-Ras (Unr), an RBP with multiple RBDs and a model system for understanding sequence specificity of modular RBDs toward target RNAs. In *Drosophila*, Unr (dUnr) performs sex-specific roles during dosage compensation. In female flies, dUnr, together with Sex-lethal (Sxl), represses translation of Male-specific lethal 2 (Msl2), the rate-limiting component of the dosage compensation complex (DCC) (Abaza and Gebauer, 2008; Abaza et al., 2006; Duncan et al., 2006), whereas in male flies, dUnr has an opposite role and acts together with the RNA helicase Maleless (Mle) presumably as an RNA chaperone to remodel the lncRNA roX2 (RNA on X 2), which promotes the assembly of the DCC (Militti et al., 2014). The human ortholog, Unr/CSDE1, is linked to several cellular processes, including cell migration, differentiation, and apoptosis, where it predominantly acts as a cytoplasmic RBP to regulate translation and stability of its target mRNAs (Boussadia et al., 2003; Dormoy-Raclet et al., 2005). Attesting to its important roles in post-transcriptional regulation, Unr/CSDE1 has been linked to diseases, including Diamond-Blackfan anemia, autism, and cancer progression (Fishbein et al., 2017; Guo et al., 2019; Horos and von Lindern, 2012; Sanders et al., 2012; Wurth et al., 2016; Xia et al., 2014).

Previous studies have suggested that Unr, a 1,039-amino acid (aa)-long protein, uses five cold-shock domains (CSDs), distributed evenly throughout the protein sequence, to engage the target RNAs (Jacquemin-Sablon et al., 1994). In this model, CSDs account for about 31% of the entire protein, leading to the suggestion that the rest of the protein was unstructured. Prior structural analysis has focused on the first CSD (Hennig et al., 2014a). Although the work provided an initial view of this region, questions related to target specificity, especially in the context of the full-length protein, were not addressed beyond observations that CSDs in isolation are relatively promiscuous RNA binders (Graumann et al., 1997; Jiang et al., 1997; Kljashtorny et al., 2015; Sachs et al., 2012; Yang et al., 2019a, 2019b; Zou et al., 2020), and that target specificity of Unr might increase for full-length Unr or during cooperative binding with other RBPs (Hennig et al., 2014a).

In this study, we employ a range of biochemical, structural, cell-based, RIP-seq (RNA immunoprecipitation coupled with next generation sequencing), and proteomic approaches to investigate the structural and RNA binding properties of Unr. Our multipronged approach led to an unexpected discovery that Unr contains four additional CSDs. These CSDs display high structural similarity to the five previously identified CSDs. However, we demonstrate that they do not bind RNA directly, but rather play a scaffolding role and make interdomain contacts that stabilize the protein and interactions with RNA and other binding partners within the Unr interactome. Thus, these non-canonical CSDs (ncCSDs) represent a new paradigm in RBD/RNA recognition, whereby structured pseudo-RBDs and interdomain interactions influence RNP target specificity.

RESULTS

Unr Contains Novel ncCSDs

For structure determination of dUnr, we initially employed the "divide and conquer" approach and tested 117 constructs with different boundaries for expression and solubility (Table S1). The boundaries were chosen to encompass predicted CSDs (Figure 1A) and N- and C-terminal extensions, based on secondary structure predictions using JPred4 (Drozdetskiy et al., 2015). Surprisingly, most soluble constructs included additional regions beyond the predicted CSD boundaries and exhibited features of structured domains based on $^{1}H,^{15}N$-HSQC NMR spectra (Figure S1A). We solved crystal and/or NMR structures of four different constructs (Figures 1B and S1B–S1D; Tables S2 and S3; PDB: 6Y6M, 6Y6E, 6Y4H, and 6Y96), which clearly showed the presence of additional CSDs in between those that have already been identified (Figure 1B; Figures S1E–S1G). These additional CSDs are structurally highly homologous to other CSDs (Figures S1E–S1G, with root-mean-square deviations [RMSDs] between 0.7 and 2.5 Å) and showed the

(C) Sequence alignment of *Drosophila* canonical CSDs and ncCSDs. The same or similar residues between all domains are colored; similar residue regions between the canonical CSDs are highlighted by red boxes, which align with the two RNA-binding regions (Y/FGF and FFHF). Additional loops only present in ncCSDs are highlighted by a blue box. The alignment has been done using Emboss Needle (Madeira et al., 2019), and ESPript (Robert and Gouet, 2014) has been used for illustration.
(D) Search results of UniProtKB, using a new hidden Markov model based on an ncCSD sequence alignment (Figure S1J). Examples of different protein families containing the predicted ncCSDs are highlighted.

typical arrangement of five antiparallel β-sheets forming a β-barrel. However, these additional domains lack the conserved canonical RNA-binding residues (FGF and (F/Y)FH; Figure 1C), which on the structures of canonical CSDs were found to point to the outside of the barrel (Figure S1G). Another distinct feature is an extended loop between β strands β1 and β2 (Figures 1C and S1E), which may act as a protein-protein interaction platform. Based on these differences, we refer to these newly discovered domains as ncCSDs and revise the existing Unr model to include the additional four ncCSDs (here numbered 2, 4, 6, and 8) in between the originally annotated five CSDs (now numbered 1, 3, 5, 7, and 9) (Figure 1B).

The presence of these ncCSDs in human Unr/CSDE1 was confirmed using $^{1}H,^{15}N$-HSQC spectra (Figure S1H) and the revised sequence alignment that showed that Unr/CSDE1 ncCSDs also lack RNA-binding residues (Figure S1I). Previously, the hidden Markov model, which was used to identify CSDs, has been trained on these RNA-binding motifs (Pfam: PF00313) (El-Gebali et al., 2019), explaining why these novel ncCSDs passed unnoticed. Of note, the UniProt entry of human Unr (hUnr), but not dUnr, annotates nine CSDs to the sequence. However, this information can be traced back to an entry from the year 2000, and the basis for this prediction is unclear. Nevertheless, all publications about Unr mention five CSDs, whether human or other species. We used HMMer (Potter et al., 2018) to generate a new hidden Markov model based on the conserved hydrophobic core residues of ncCSDs, and iteratively searched the sequence database of UniProtKB (Bateman, 2019) for the existence of ncCSDs in other proteins (Figure 1D). Besides all Unr-related proteins, ncCSDs were also found in other proteins that feature canonical CSDs (e.g., cold-shock DNA-binding domain protein [*Clostridium sp. CAP:1000*]; R3H domain-containing protein [*Phytophthora ramorum*]). Similar to Unr, ncCSDs are most often found in spacing regions between canonical CSDs. However, there is also a class of 25 proteins, without any other domain prediction. In total, 1,038 proteins across species were predicted to contain ncCSDs (Figure 1D).

Taken together, our systematic and unbiased approach to structural characterization of Unr revealed the presence of ncCSDs that lack residues implicated in RNA binding dispersed in between the canonical CSDs. Moreover, we overcame technical challenges to generate high-quality samples that enabled us to conduct in-depth mechanistic studies of how multi-domain RBPs that feature both canonical RBDs (CSD) and pseudo-RBDs (ncCSDs) engage target RNA.

RNA Binding in a Multi-domain Context with Canonical CSDs and ncCSDs

We subjected our multi-domain constructs to further biophysical analysis aimed at quantifying their RNA binding and understanding the roles of different domains in RNA recognition. Electrophoretic mobility shift assays (EMSAs) done with constructs that contain canonical CSDs and ncCSDs (CSD123, CSD456, CSD789) show binding of all tested proteins to stem loops 6 and 7 of roX2 lncRNA (Figure 2A). CSD456, featuring only one canonical CSD, binds with a K_D of 16 μM, comparable with the affinity of CSD1 alone (Hennig et al., 2014a). Similar affinity was seen for CSD789 that includes two canonical CSDs (K_D of 32 μM; Figure 2A). Interestingly, RNA-binding affinities measured by NMR of both CSD78 and CSD9 constructs were found to be significantly weaker (around 200–300 μM; Figures S2A and S2B), suggesting that synergistic binding within CSD789 may play a major role in enhancing RNA-binding affinity. Concomitant with these observations, an NMR titration of CSD789 with an A15-mer RNA results in chemical shift perturbations (CSPs) of residues in all three domains in the intermediate-to-slow exchange regime (Figure S2C) as opposed to the single domains, which showed binding in the fast exchange regime (Figures S2A and S2E), indicating that the affinity of CSD789 toward RNA is considerably stronger than that of the CSD78 or CSD9 constructs.

We observed that CSD123 compared with CSD456 and CSD789 binds RNA with much higher affinity (1–2 μM), potentially because of the higher theoretical isoelectric point of CSD1 (8.03, versus average of 6.06 for other CSDs). This affinity approaches the one measured for full-length Unr (0.5–1 μM; Figure 2A) and is in agreement with the observation that the Unr N-terminal CSDs are sufficient for translational repression of *msl-2* mRNA (Abaza and Gebauer, 2008).

To probe whether ncCSDs contribute to RNA binding of these multi-domain constructs, we employed NMR titration experiments with polypurine-rich 9-mer RNA (adapted from the loop of lncRNA roX2 SL6). Due to aggregation problems with CSD123 at concentrations needed for these experiments, we used CSD12 instead. Our results showed that only CSD1, but not ncCSD2, interacts with the RNA as derived from NMR CSPs (Figure 2B). Similar observations were made for CSD456, where ncCSD4 and ncCSD6 flanking CSD5 do not show major CSPs (Figure 2C). Additionally, we observed no RNA-induced CSPs for the isolated ncCSD6 construct (Figure S2D). Finally, NMR-monitored RNA titration experiments using CSD789 and CSD78 showed that in the context of these constructs, several ncCSD8 residues (e.g., R866, C867, and I868) exhibit CSPs (Figures 2D and S2E). However, titration of RNA to an isolated ncCSD8 did not induce CSPs (Figure S2F), suggesting that the effects seen in the multi-domain constructs are likely due to proximity effects, as a positively charged area on ncCSD8 is located close to the RNA-binding interface of CSD7 (Figure S2G). Taken together, our RNA-binding studies indicate that although ncCSDs do not interact with RNA in isolation, they may contribute to RNA binding. Additionally, we also observed cooperativity effects when RNA affinity was measured in the context of multi-domain constructs that include both canonical and ncCSDs.

Interdomain Contacts Mediate Fixed Spacing and Orientation between Canonical CSDs and ncCSDs

To define the factors that impact the cooperativity further, we analyzed structures of multi-domain constructs used in this study to map the residues involved in interdomain contacts and examine their roles. In the crystal structure of CSD456, interdomain contacts between CSD5 and both ncCSD4 and ncCSD6 are clearly discernable and mediated by F477 on ncCSD4; L505, T521, R533, Q538, E547, L549, and R582 on CSD5; and F593, N663, and R662 on ncCSD6 (Figure 3A). These contacts appear

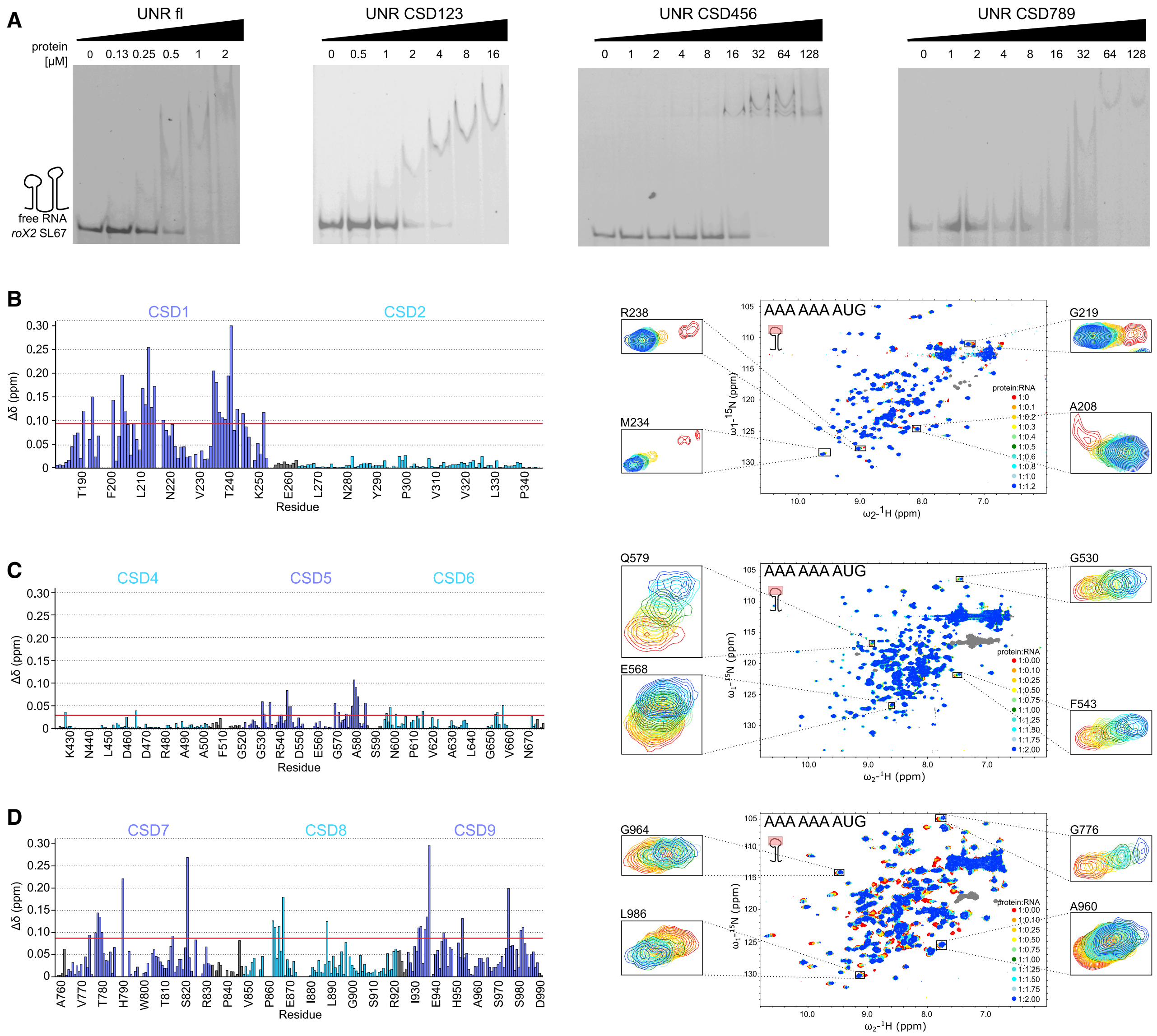

Figure 2. RNA Binding of Unr Constructs

(A) EMSAs show binding of Unr full-length and different triple-domain constructs to the stem loops 6 and 7 of lncRNA roX2 (see inset).

(B–D) ^{1}H,^{15}N-HSQC NMR titration experiments with different amounts of 9-mer RNA (5′-AAA AAA AUG-3′), derived from the stem loop 6 of roX2 and the derived chemical shift perturbation (CSP) plots, for CSD12 (B), CSD456 (C), and CSD789 (D). The red line in the CSP plots indicates the value of the average plus the standard deviation of all measured shifts.

to keep the domains at a certain distance and orientation to each other (Figure 3A). To confirm that these contacts are also present in solution and are not artifacts due to crystal packing, we used small-angle X-ray scattering (SAXS). The fit between experimentally observed scattering densities and back-calculated scattering densities from the crystal structure (χ of 1.02; SASBDB: SASDHJ7) strongly suggests that the fixed domain arrangement seen in the crystal structure is maintained in solution (Figures 3A and S3A; Table S4). Concomitant with these observations, the NMR structure of CSD78 also has a fixed domain-domain distance and orientation, verified by 49 interdomain and 48 domain-linker NOE-based distance restraints (Figures 3B and 3C). This interface mostly consists of hydrophobic interactions formed by residues R765, F767, A769, L803, and E806 on CSD7, and I837, Y865, I887, and T888 on ncCSD8. The fixed domain arrangement and overall conformation were additionally confirmed by SAXS (Figures 3B and S3A; Table S4; χ = 1.1; SASBDB: SASDHK7) and ^{15}N NMR relaxation data, which provide a measure of dynamics on a residue resolution level. The rotational correlation time is similar over all the residues in CSD78 and too elevated if independent molecular tumbling of each domain is assumed (CSD78: τ_c = 12.7 ± 1.2 ns), indicative for a joint tumbling of the two domains (Figures 3D and S3B; Table S5).

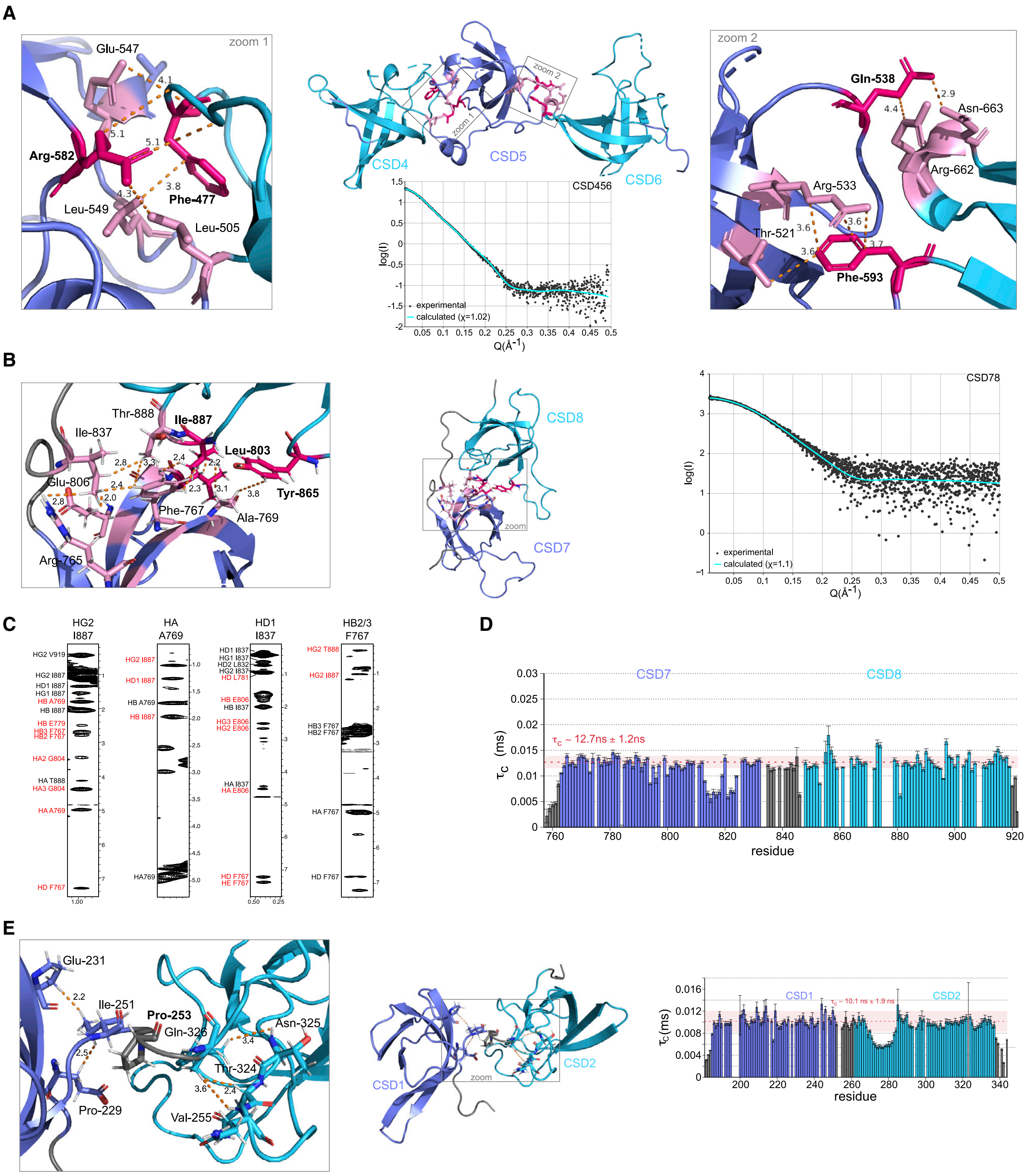

Figure 3. Interdomain Contacts between Canonical CSDs and ncCSDs within Unr

(A) Interdomain contacts between CSD4 and CSD5 and between CSD5 and CSD6 derived from the crystal structure are highlighted in pink (boldfaced labeled residues colored in hot pink are mutated in experiments described below). The SAXS scattering curve of dCSD456 in solution (black dots) fits the back-calculated scattering density of the crystal structure (cyan) (χ = 1.02; SASBDB: SASDHJ7).

(B) The NMR solution structure of CSD78 shows a network of NOEs between both domains. These mostly hydrophobic interactions keep the domain arrangement fixed (highlighted in pink; residues labeled boldfaced and colored in hot pink are mutated in experiments described below). The SAXS scattering curve of dCSD78 in solution (black dots) fits to the back-calculated scattering density of the NMR structure (cyan) (χ = 1.1; SASBDB: SASDHK7).

(legend continued on next page)

^{15}N NMR relaxation experiments showed the same observation for CSD12. The high rotational correlation time (τ_c = 10.1 ± 1.9 ns) suggests that the two domains have a limited freedom of movement with respect to each other (Figures 3E and S3C; Table S5). This indicates a joint tumbling of the two domains as well. Although interdomain NOEs are lacking, linker-domain NOEs between CSD1 and linker region residues up to A252 and NOEs between CSD2 and linker starting after residue E254 may explain this. Thus, there are only three residues in the linker with only sequential and intra-residue NOEs, of which the central residue is a proline (P253) (Figure 3C), which can increase linker rigidity (Poon et al., 2007; Receveur et al., 2002). Despite the absence of rigid domain-domain interactions, the two single domains within CSD12 have a limited freedom of movement with respect to each other.

Interestingly, the already mentioned extended loop between β strands β1 and β2 present in ncCSDs, but not canonical CSDs, is flexible in ncCSD2 (Figures 3E and S3C). Also, electron density in the corresponding region in ncCSD4 and ncCSD6 was weak or absent in X-ray diffraction data of CSD456, indicating flexibility. On the other hand, ^{15}N relaxation data of CSD78 do not show decreased rotational correlation times for corresponding residues in ncCSD8, most likely because of interdomain interactions with CSD7. Because the amino acid sequence in this region is not conserved and also domain-domain interactions between nCSD4-CSD5, CSD5-nCSD6, and CSD7-nCSD8 are of different compositions and at different locations, it cannot be concluded that this extended loop region is a general CSD-CSD interaction interface (Figure S3D).

Taken together, although some linkers between canonical CSDs and ncCSDs may remain flexible, ncCSDs clearly impose spatial restrictions on canonical CSDs. We hypothesize that ncCSDs act as scaffold domains to maintain distance and orientation between the canonical CSDs and restrict conformational flexibility. Thus, ncCSDs might indirectly contribute to RNA binding by positioning the RNA-binding motifs of canonical CSDs (or potential protein-interaction surfaces) toward target RNAs and/or RNP complexes, and as such induce RNA/RNP tertiary structure specificity.

Interdomain Contacts in Full-Length Unr Impact Protein Stability, RNA Binding, and Translation

We next examined the role of interdomain contacts in stabilizing the overall structure of full-length Unr and impacting its function. We focused our analysis on interdomain residues located in loop regions that lack secondary structure, yet were identified to form interdomain contacts. We generated a series of single mutants as shown in Figure 4A, as well as double and triple mutants as follows: F477A/R582A (referred to as 45 ID given that it disrupts interdomain interactions between CSDs 4 and 5), Q538A/F593A (56 ID, disrupting CSDs 5 and 6 interactions), and L803A/Y865A/I887A (78 ID, disrupting CSDs 7 and 8 interface) (Figures 3A, 3B, and 4A). Overall, CSD456 and CSD78 mutants displayed decreased stability when compared with wild-type (WT) CSD456 and CSD78, as established by measuring melting temperatures using differential scanning fluorimetry (DSF) (Figure 4B). As can be seen, even single-point mutants displayed a significant decrease in melting temperature of up to 10°C compared with the WT, suggesting that the structural integrity of these constructs has been substantially compromised. Our ^{1}H,^{15}N-HSQC experiments and SAXS data, as well as the decreased solubility observed during the purification process, all further support this view (Figures S4A–S4C). The aggregation potential observed in ^{1}H,^{15}N-HSQC spectra of different single-mutant CSD78 proteins is not as drastic as for most of the CSD456 mutants (Figure S4C). Aggregation is stronger for the Y865A mutant, followed by the I887A mutant. The L803A mutant is the only one not showing visible aggregation, but strong and numerous CSPs indicative of perturbed domain-domain interactions. The unvaried peak dispersion, however, shows that the overall fold is retained (Figure S4C). Strikingly, in the context of the full-length protein, single and double mutations between CSD45 and CSD56 had a minor influence on full-length protein stability (Figure 4B). To further assess the structural integrity of CSDs in Unr full-length mutants, we employed circular dichroism (CD) spectroscopy (Figure S4D). The derived secondary structure content in WT and mutants was similar, indicating that the mutations indeed affect only the interdomain contacts, but not the overall CSD domain fold (Figure S4D). Thus, mutants in a full-length context allow for meaningful *in vitro* and cellular functional studies at least for the single and double mutants between CSD45 and CSD56.

An exception in both measurements is the single mutation of Q538A, suggesting that this mutation might not disrupt the interdomain contacts efficiently.

We used the full-length Unr mutants (456 ID and 456-78 ID) to further test our hypothesis that interdomain contacts are important determinants of RNA specificity. We performed RIP-seq experiments in cells transfected with V5-tagged Unr WT, 456 ID, 456-78 ID, or an empty V5-tag vector (background control). Differences in pulled down RNAs could be observed between the WT protein sample and the interdomain mutants (456 ID and 456-78 ID). However, the total number of significantly enriched genes is higher in mutant pull-downs. 456 ID shows 218 and 456-78 ID 531 genes that are significantly enriched compared with the WT protein. On the contrary, only 75 genes are significantly enriched

(C) Exemplary NOE strips of the 3D ^{13}C,^{1}H,^{1}H HMQC-NOESY spectrum of CSD78 highlight interdomain NOEs (red) that were detected and used in structure calculations.

(D) ^{15}N relaxation data of CSD78 indicating joint tumbling of CSD7 and CSD8 in solution, with flexible regions (residues between R813 and L824) within the domains (flexible loop between β strands 4 and 5 of CSD7).

(E) CSD12 is lacking interdomain NOEs but shows only a three-residue-long linker, without NOEs, including one proline (labeled bold). Relaxation data of CSD12, indicating tumbling of the two single domains together in solution. Flexible residues are located between L270 and T285, which corresponds to an interdomain loop between β strand 1 and 2 in CSD2. The rotational correlation time (τ_c) derived from ^{15}N longitudinal and transverse relaxation experiments is plotted per residue. The error bars indicate the error propagation from errors of the two relaxation experiments, which are derived from the quality of the exponential fit and the deviation between duplicates of two different relaxation delays.

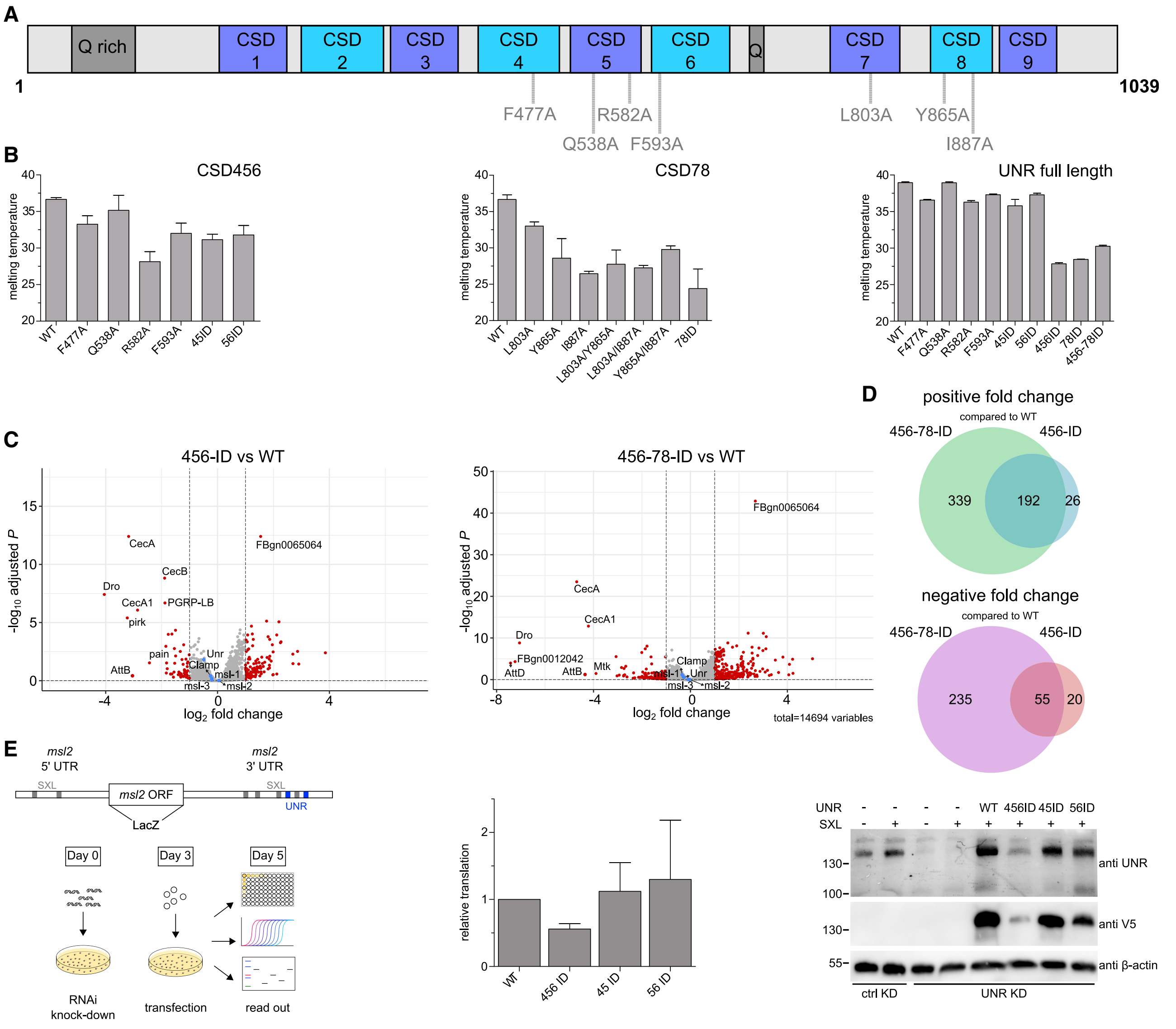

Figure 4. Unr Interdomain Contacts Play a Role in RNA Target Selectivity and Translational Repression

(A) Schematic presentation of the inserted mutations within the full-length protein.

(B) Melting temperatures for CSD456, CSD78, and full-length Unr wild-type (WT) and mutants as determined by DSF. Measurements were done in duplicates. Shown is the mean, and the error bars indicate the standard error.

(C) Volcano plots showing the difference of RNA targets of the RIP-seq experiment between the WT and interdomain mutant samples (456 ID left graph, 456-78 ID right graph). The log2(fold change) is plotted against the log10 adjusted p value; n = 3.

(D) Venn diagrams showing the numbers of positive (upper graph) and negative (lower graph) enriched targets of the different mutants compared with the WT.

(E) Left: schematic representation of the experimental setup of the cellular reporter gene assay and the used reporter gene construct. The 5′ and 3′ UTRs are derived from the *msl2* mRNA exhibiting the binding sites for SXL (gray) and Unr (blue); the open reading frame is coding for β-galactosidase. Middle: relative translation of β-galactosidase after expression of different Unr mutants normalized to the level of the WT protein. Translation levels were further normalized to the ones from Renilla and the levels of the reporter RNA; n = 3. Shown is the mean, and the error bars indicate the standard error. Right: knockdown and transfection efficiency assessed by western blot analysis. The upper band in the anti-Unr blot is full-length Unr, and the lower band is a C-terminal truncation.

in the WT over the 456 ID mutant and 290 over the 456-78 ID. 192 genes are overlapping in the positively enriched and 55 in the negatively enriched samples between the two mutants. The total number of differentially bound mRNAs increases with the number of mutations, and most of the changes in 456 ID were also seen in the 456-78 ID mutant, indicating that higher conformational heterogeneity results in less discriminate RNA binding (Figures 4C and 4D). Due to higher protein levels in the pulled down samples of the WT (Figure S4E), we cannot say with certainty whether the enriched RNAs in the WT samples are due to different binding behavior to these RNAs or to the different input amount. However, these data indicate that, once the conformational heterogeneity of

CellPress
OPEN ACCESS

Unr is increased due to mutations, more RNAs are bound and pulled down.

To test the effects of this different RNA-binding behavior on the protein function, we performed reporter gene assays in SL2 cells using *msl-2* mRNA as previously described (Duncan et al., 2006; Graindorge et al., 2013; Hennig et al., 2014a). We first depleted endogenous Unr and, 3 days after depletion, we transfected a β-galactosidase reporter gene construct containing the 5′ and 3′ UTRs of *msl2* mRNA, together with a Renilla control plasmid and plasmids encoding for Sxl and V5-tagged Unr (Figure 4E). The interdomain mutant 456 ID shows a significantly higher translational repression of the target mRNA (Figure 4E), despite having lower cellular protein levels (Figure 4E, right panel), indicating either increased binding to the reporter or a strengthened translation repression. Mutation of only one of the interdomain interfaces does not show a significant difference with respect to the WT protein. Altogether, these data show that the scaffolding role of ncCSDs and their influence on reduction of conformational heterogeneity have an influence on protein function and change mRNA target specificity of Unr.

Unr Protein Shape Influences Translation Regulation and Binding Partner Interactions

To assess whether the observed differences in translational repression are due to different binding of Unr to the target RNA or to a direct effect on translation, we used an *in vitro* tethering translation assay in *Drosophila* embryo extracts as described by Abaza and Gebauer (2008). For tethering, we used a construct containing the Firefly luciferase open reading frame (ORF) and a 3′ UTR consisting of nine MS2 binding sites (Figure 5A, left panel). Recombinant MS2-tagged Unr was then added, and luciferase levels were measured as a proxy of translation. As controls, Renilla luciferase without MS2 binding sites was co-translated, and the data were corrected for variations in Renilla luciferase levels. As negative controls, we added untagged Unr and an unrelated MS2-tagged protein (MBP-MS2). The results showed that, compared with WT Unr, the tested mutants showed decreased activity in translational repression for low protein concentrations (Figure 5A, middle panel; Figure S5A). These effects are unrelated to variations in mRNA levels (Figure 5A, right panel; Figure S5A), supporting the conclusion of differential translational regulation by the Unr constructs. Because tethering separates the role of Unr in translation from that in RNA binding, we conclude that the fixed interdomain orientation between CSD456 promotes the regulation of translational repression by Unr independent of the RNA-binding event. Compared with results shown in Figure 4E, these data also suggest that the differences observed *in cellulo* are due to altered interactions of Unr interdomain mutants with mRNA and/or other protein binding partners.

To investigate the binding of Unr interdomain mutants to protein partners and map their RNP composition, we performed a protein immunoprecipitation assay followed by comparative quantitative mass spectrometry (MS) for V5-tagged WT Unr and ID mutants expressed in SL2 cells after depletion of endogenous Unr. To distinguish between direct and RNA-mediated protein-protein interactions, we prepared samples with and without RNase treatment. As observed in the cellular assays described above (Figure 4E), mutant 456-78 ID had a lower expression level in cells, which inevitably led to lower Unr concentrations in the pull-downs (Figures 5B and S5B). Consequently, enriched protein hits for the mutant sample, especially in cluster 5, were subunits of the proteasome (Figures 5C, 5D, S5B, and S5C, cluster 5), which indicates that mutant Unr has a severely affected stability and is targeted by the protein quality-control system. As a consequence, we cannot definitively claim that the proteins are depleted in mutant Unr samples as a consequence of lower affinity for mutant versus WT Unr. However, focusing on Unr interactors that decreased in the 456-78 ID background compared with WT, we found that only 32 candidates are enriched in the WT (Figures 5C and 5D, clusters 1 and 2). Thus, having a mutation that brings down the Unr levels enabled us to define the Unr interactome and define the composition of Unr RNPs (Figures 5C and 5D, clusters 1 and 2). All of the 32 hits are RBPs, and some of them are well-known regulators of translation or mRNA degradation. Notably, our analysis was able to identify a previously reported binding partner of Unr, poly(A)-binding protein (PABP) (Chang et al., 2004; Duncan et al., 2009), which we find to be enriched even in RNase-treated samples, indicating that this interaction is RNA independent. In contrast, other targets show reduced binding after RNase treatment, showing that binding is likely mediated by RNA. One example for an RNA-dependent interaction is Sister-of-sex-lethal (Ssx), which is a homolog of Sxl, known to exhibit comparable *msl-2* mRNA-binding activity while being incapable of engaging Unr directly (Moschall et al., 2019). This consensus with previous studies further strengthens the reliability of our results. Additional hits of special interest include Hrb27c (Hrp48), which is known to be a necessary component of the *msl2* mRNA translation repression complex together with Sxl and Unr (Szostak et al., 2018). Other interaction partners we identified include proteins that play a role in miRNA processing (Ago1; Okamura et al., 2004), splicing (Rm62 [Bates et al., 2005; Lin et al., 2005], Nito [Lence et al., 2016], and Rump [Gattoni et al., 1996; King et al., 2014]), RNA degradation (Upf1 [Gatfield et al., 2003] and Edc3 [Fenger-Grøn et al., 2005]), and RNA localization (Imp [Nielsen et al., 2001], tral [Wilhelm et al., 2005], and Ref1 [Rodrigues et al., 2001]). Another striking fact is that some of the targets, such as 4E (Lim et al., 2011; Wang et al., 2017), tral, Hrb27C, and Imp (Hansen et al., 2015), are already previously identified PABP interaction partners, suggesting that these proteins may be involved in formation of a larger RNP complex. A STRING analysis shows a potential network of known interactions between most of the hits (Figure 5E).

Taken together, these results suggest that domain organization within the full-length Unr is of critical importance for its role in translation and for maintaining the integrity of the Unr RNA interactome. In this context, Unr interdomain interactions between canonical CSDs and ncCSDs are determinants of RNA tertiary structure specificity. Further, by using the mutant protein as a binding-decreased interaction control of Unr in our proteomics analysis, we could gain insight into the Unr interactome and its specific role in several cellular processes and complexes.

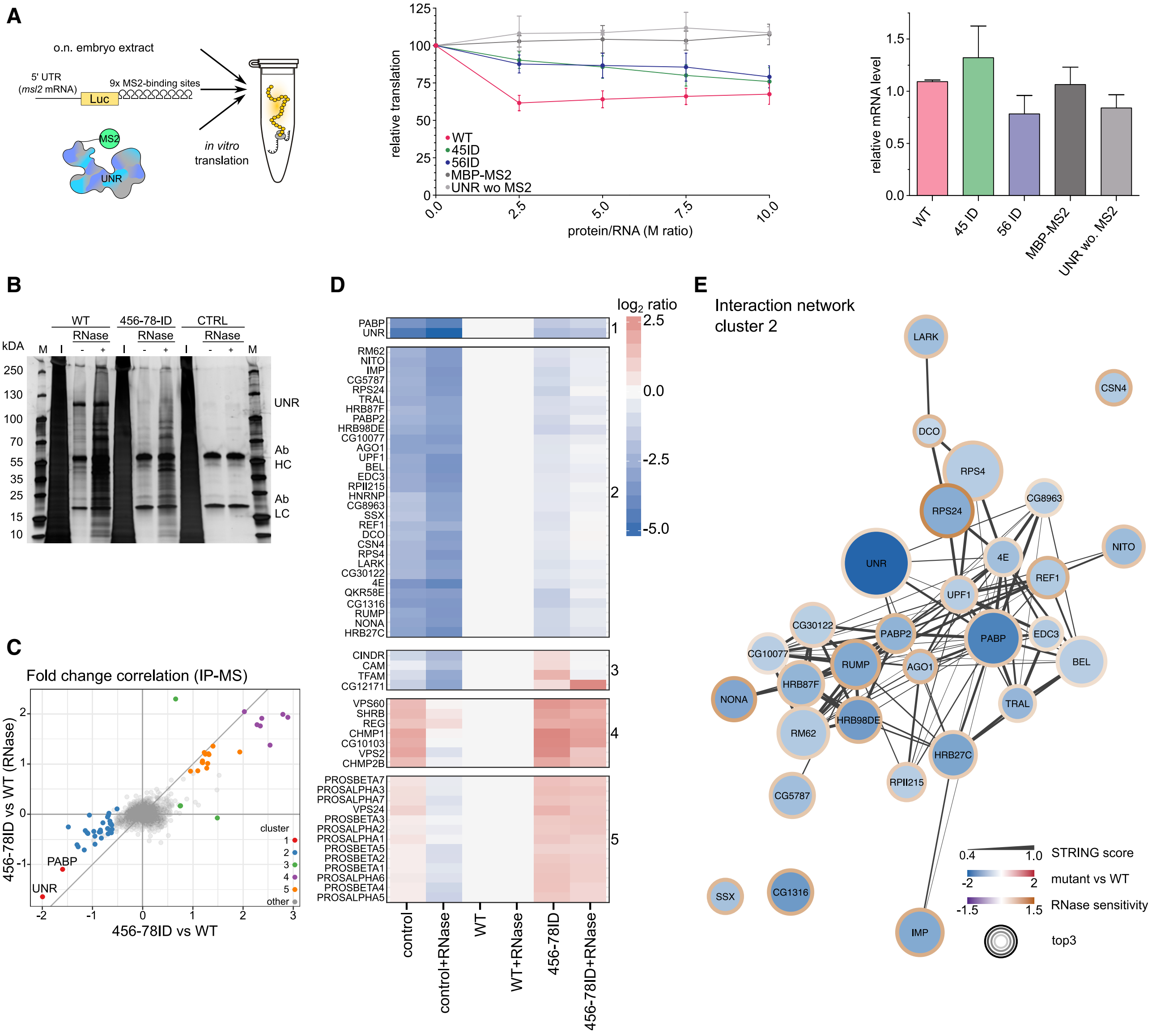

Figure 5. Influence of Unr Interdomain Contacts on Cofactor Binding

(A) Schematic representation of the *in vitro* translation assay. MS2-tagged Unr gets tethered to the 3′ UTR, which contains nine MS2 binding loops. Binding leads to repression of translation. The same mRNA construct was used before (Abaza and Gebauer, 2008) (BmutLMS2). Middle: relative *in vitro* translation of the firefly reporter gene over the internal control Renilla after adding increasing amounts of Unr; n = 3. Right: relative reporter mRNA levels after *in vitro* translation (point of 2.5 molar excess of Unr over RNA), measured by qRT-PCR; n = 3. The mean is shown for each data point, and the error bars indicate the standard deviation.

(B) A silver-stained polyacrylamide gel, showing the input (I) and elution samples with (+) and without (−) RNase treatment for WT, 456-78 ID, and the empty vector control that were used for the proteomics analysis. Unr, the antibody heavy chain (Ab HC), and the antibody light chain (ab LC) are labeled at the side of the gel.

(C) Scatterplot showing the correlation of interdomain mutant versus WT (x axis) and RNase-treated interdomain mutant versus RNase-treated WT (y axis). Colors indicate the cluster number from (D); n = 3.

(D) Heatmap representation of the different protein targets of WT and mutant protein samples. Proteins were clustered in five clusters using k means algorithm. The RNase-treated samples were normalized to the RNase-treated WT sample, and the non-treated samples were normalized to the non-treated WT sample. Red indicates upregulation and blue downregulation; n = 3.

(E) STRING interaction network, showing possible interactions between the targets that were enriched for the WT samples shown in clusters 1 and 2 of (D). The line thickness represents the STRING score, which represents the strength of data support for the individual interactions (Szklarczyk et al., 2019). The inner color of each circle represents the fold-change between interdomain mutant and WT sample; the border line color indicates the difference of enrichments of interdomain mutant versus WT between RNase-treated and non-treated (RNase sensitivity), and the size indicates the average abundance of a protein in the immunoprecipitation mass spectrometry run (top3 value), which correlates with the pull-down efficiency.

DISCUSSION

Understanding protein-RNA recognition and RNA-binding specificity is a prerequisite for obtaining mechanistic and functional insights of how RBPs regulate RNA fate. However, the number of available high-resolution structures of multi-domain RBPs remains very limited. In the work reported here, we describe several high-resolution structures that dissect the multi-domain organization of Unr. Importantly, we discovered that Unr contains previously unknown ncCSDs. As we show here, ncCSDs display a high degree of structural similarities to CSDs but lack RNA-binding motifs, which might have been the reason why they remained cryptic and unannotated until now (Figures 1A, 1C, and S1B–S1J). This suggests that ncCSDs might have been overlooked in other proteins as well, and a hidden Markov model-based iterative search through UniProtKB (Bateman, 2019) indicates that these ncCSDs are not only present in *Drosophila* Unr, but are in several other proteins throughout different phyla, often located close to canonical CSDs in an alternating fashion (Figure S1D). Due to this strong coappearance of CSDs and ncCSDs within one protein and due to their high similarity in structure, it is likely that they evolved from the same domain, maybe by domain duplication (Bagowski et al., 2010). However, it is currently not clear whether canonical CSDs lost RNA-binding residues during evolution to form ncCSDs or vice versa. Based on these observations, we think that ncCSDs represent an important feature of many proteins that justify further study in order to fully understand the range of their biological roles.

The work reveals an essential scaffolding role for ncCSDs in Unr. Although ncCSDs lack the ability to bind RNA independently, they mediate interdomain contacts that impact overall protein stability (Figures 2 and 3), and we propose that ncCSDs have an essential role in RNA tertiary structure specificity, translational activity, as well as Unr RNP assembly and composition. Mutational studies in embryo extracts and SL2 cells demonstrated that disruption of this scaffolding function affects translation, which means that these mutations affect RNA binding, RNP composition, and/or RNP interactions with molecular machineries (Figures 4E and 5A). Indeed, RIP-seq experiments showed that interdomain mutations lead to differential binding to RNA targets by Unr (Figures 4C and 4D), strongly supporting the proposed scaffolding role.

The ultimate verification of our hypothesis will require structural characterization of the full-length Unr in complex with its target RNA. Given the challenges of preparing stable full-length Unr or CSD1-9 samples, investigations of this complex will likely need to be done in the context of a Unr-dependent RNP. Toward this end, we report here identification of 31 proteins that may be involved in the potential RNP-interactome of *Drosophila* Unr (Figures 5D and 5E). Thus, this work will inform future efforts to reconstitute an entire RNP for structural studies.

Structural studies with RNA would also provide insight into the exact role of each of the domains in Unr biology. Our results and those reported before show that full-length Unr interacts with the model RNA sequence (SL67 of roX2 lncRNA) with a binding affinity similar to that of the N-terminal construct CSD123. This would suggest that in this context, CSD123 would be sufficient for normal physiology. However, although the N-terminal half of Unr is sufficient for early fly development, the full-length protein is necessary for fly viability at later stages. Flies expressing a truncated version of Unr (CSD1-6 plus the first Q-rich domain) die shortly after eclosion and present dramatic defects on dosage compensation (Patalano et al., 2009), indicating that CSDs and ncCSDs other than CSD123 have an essential role in proper protein function. Given that all canonical CSDs share the same RNA-binding residues, have a similar fold, and represent highly promiscuous RNA binders (Hennig et al., 2014a; Triqueneaux et al., 1999), we would expect to see CSD123 being able to compensate for other domains, which is not the case. Instead, we propose that ncCSDs support binding of Unr to structured RNA by reducing conformational heterogeneity. Single-stranded regions within RNAs with an ideal three-dimensional fold could be bound by each RNA-binding motif of canonical CSDs, resulting in a cumulative high-affinity binding. RNAs that have a different, less recognizable fold would be bound by fewer CSDs, resulting in weaker binding. In this model, ncCSDs impose conformational restrictions on the orientations of CSDs and therefore define the target specificity, as well as allow for cooperative mechanisms of binding. Moreover, ncCSDs are likely involved in forming specific protein-protein interactions, which further places restrictions on the ultimate RNA sequence specificity. Thus, despite the fact that each individual CSD is relatively promiscuous and uses short RNA sequences for binding, collectively, restraints imposed by ncCSDs and additional binding partners result in a limited scope of RNA features that the RNP can select for. More speculatively, ncCSDs may represent a new class of pseudo-RBDs, domains that structurally resemble classical RBDs but lack residues critical for RNA binding. Although our analysis suggests that ncCSDs are present in a wide range of proteins across species, future studies will be needed to explore the idea of pseudo-RBDs further. If proven to be relevant to other RBDs, we expect that pseudo-RBDs may become an important platform for synthetic biology and protein-protein interaction inhibitor development.

Overall, the study presented here, as well as previous work on IMP3 integrating SELEX and structural data (Schneider et al., 2019), show the importance of developing new methods to detect, characterize, and predict protein-RNA interactions at a global scale. We expect that the work reported here will result in a significant number of revisions to CSD-containing proteins because many of them are likely to contain ncCSDs. Furthermore, advancing systematic strategies for structural, biophysical, transcriptomic, and proteomic characterization of RBPs (Dimitrova-Paternoga et al., 2020), such as the one described here, are essential for uncovering the remaining mysteries of RNA regulation in health and disease.

STAR★METHODS

Detailed methods are provided in the online version of this paper and include the following:

- KEY RESOURCES TABLE
- RESOURCE AVAILABILITY
  - Lead Contact

- Materials Availability
- Data and Code Availability
- EXPERIMENTAL MODEL AND SUBJECT DETAILS
  - Bacterial Strains and Culture Conditions to generate plasmids and express protein
  - Culturing of Schneider's *Drosophila* Line 2 (SL2)
- METHOD DETAILS
  - Plasmids
  - Protein Purification
  - Crystal structure determination
  - NMR spectroscopy
  - SAXS data acquisition and analysis
  - Electrophoretic mobility-shift assays (EMSA)
  - Protein melting temperature
  - Circular dichroism
  - *In vitro* translation assay
  - RNAi, transfection and reporter gene assay
  - Unr immunoprecipitation
  - RNA sequencing and data analysis
  - Sample preparation and LC-MS/MS analysis of IP-MS samples
  - Data analysis of mass spectrometry experiments
  - Sequence alignment and HMMER prediction
  - Data presentation
- QUANTIFICATION AND STATISTICAL ANALYSIS

SUPPLEMENTAL INFORMATION

Supplemental Information can be found online at https://doi.org/10.1016/j.celrep.2020.107930.

ACKNOWLEDGMENTS

We thank the ESRF Grenoble (beamlines BM29 and ID-30A) and DESY Hamburg PETRA-3 (P12 beamline) local contacts for support (Martha Brennich and Alexey Kikhney). We gratefully acknowledge Kathryn Perez at the EMBL Protein Expression and Purification Facility for assisting with the NanoDSF and CD measurements. We also thank Martin Beck for critically reading and constructive criticism of parts of the manuscript. This work was supported by an EIPOD fellowship to P.K.A.J. cofunded by the EMBL and Marie Curie Actions Cofund grant MSCA-COFUND-FP. F.G. was supported by grants from the Spanish Ministry of Science and Innovation and Universities (MICINN, PGC2018-099697-B-100), "la Caixa" Foundation (ID 100010434) under the agreement LCF/PR/HR17/52150016, the Catalan Government (2017SGR534), and the Centre of Excellence Severo Ochoa. J.H. gratefully acknowledges support via an Emmy-Noether Fellowship and the Priority Program SPP1935 of the Deutsche Forschungsgemeinschaft (DFG). Finally, we thank the EMBL for funding.

AUTHOR CONTRIBUTIONS

Conceptualization, N.M.H. and J.H.; Methodology, B.S., D.M., V.B., M.M.S., F.G., and J.H.; Investigation, N.M.H., P.K.A.J., P.M., T.G., B.S., J.P., F.S., P.H., L.J.S., and J.H.; Resources, V.B., M.M.S., F.G., and J.H.; Writing – Original Draft, N.M.H. and J.H.; Writing – Review & Editing, N.M.H., P.K.A.J., P.M., T.G., B.S., J.P., F.S., P.H., L.J.S., V.B., M.M.S., F.G., and J.H.; Visualization, N.M.H., B.S., J.P., F.S., and J.H.; Supervision, V.B., M.M.S., F.G., and J.H.; Project Administration, F.G. and J.H.; Funding Acquisition, M.M.S., F.G., and J.H.

DECLARATION OF INTERESTS

The authors declare no competing interests.

Received: April 29, 2020
Revised: June 3, 2020
Accepted: June 29, 2020
Published: July 21, 2020

REFERENCES

Abaza, I., and Gebauer, F. (2008). Functional domains of Drosophila UNR in translational control. RNA *14*, 482–490.

Abaza, I., Coll, O., Patalano, S., and Gebauer, F. (2006). Drosophila UNR is required for translational repression of male-specific lethal 2 mRNA during regulation of X-chromosome dosage compensation. Genes Dev. *20*, 380–389.

Afroz, T., Cienikova, Z., Cléry, A., and Allain, F.H.T. (2015). One, Two, Three, Four! How Multiple RRMs Read the Genome Sequence. Methods Enzymol. *558*, 235–278.

Ahlner, A., Carlsson, M., Jonsson, B.H., and Lundström, P. (2013). PINT: a software for integration of peak volumes and extraction of relaxation rates. J. Biomol. NMR *56*, 191–202.

Auweter, S.D., Oberstrass, F.C., and Allain, F.H.-T. (2006). Sequence-specific binding of single-stranded RNA: is there a code for recognition? Nucleic Acids Res. *34*, 4943–4959.

Bagowski, C.P., Bruins, W., and Te Velthuis, A.J. (2010). The nature of protein domain evolution: shaping the interaction network. Curr. Genomics *11*, 368–376.

Bateman, A.; UniProt Consortium (2019). UniProt: a worldwide hub of protein knowledge. Nucleic Acids Res. *47* (*D1*), D506–D515.

Bates, G.J., Nicol, S.M., Wilson, B.J., Jacobs, A.-M.F., Bourdon, J.-C., Wardrop, J., Gregory, D.J., Lane, D.P., Perkins, N.D., and Fuller-Pace, F.V. (2005). The DEAD box protein p68: a novel transcriptional coactivator of the p53 tumour suppressor. EMBO J. *24*, 543–553.

Bieri, P., Greber, B.J., and Ban, N. (2018). High-resolution structures of mitochondrial ribosomes and their functional implications. Curr. Opin. Struct. Biol. *49*, 44–53.

Blighe, K., Rana, S., and Myles, L. (2019). EnhancedVolcano: publication-ready volcano plots with enhanced colouring and labeling. R package version 1.4.0. https://github.com/kevinblighe/EnhancedVolcano.

Boussadia, O., Niepmann, M., Créancier, L., Prats, A.-C., Dautry, F., and Jacquemin-Sablon, H. (2003). Unr is required in vivo for efficient initiation of translation from the internal ribosome entry sites of both rhinovirus and poliovirus. J. Virol. *77*, 3353–3359.

Braman, J., Papworth, C., and Greener, A. (1996). Site-directed mutagenesis using double-stranded plasmid dna templates. In In Vitro Mutagenesis Protocols, M.K. Trower, ed. (Humana Press), pp. 31–44.

Cech, T.R., and Steitz, J.A. (2014). The noncoding RNA revolution-trashing old rules to forge new ones. Cell *157*, 77–94.

Chang, T.-C., Yamashita, A., Chen, C.-Y.A., Yamashita, Y., Zhu, W., Durdan, S., Kahvejian, A., Sonenberg, N., and Shyu, A.B. (2004). UNR, a new partner of poly(A)-binding protein, plays a key role in translationally coupled mRNA turnover mediated by the c-fos major coding-region determinant. Genes Dev. *18*, 2010–2023.

Chen, H., and Boutros, P.C. (2011). P.C. VennDiagram: a package for the generation of highly-customizable Venn and Euler diagrams in R. BMC Bioinformatics 12 *35*. https://doi.org/10.1186/1471-2105-12-35.

Cléry, A., and Allain, F.H.-T. (2011). From structure to function of RNA binding proteins. In Madame Curie Bioscience Database (Landes Bioscience and Springer Science+Business Media). https://www.ncbi.nlm.nih.gov/books/NBK63528/.

Conlon, E.G., and Manley, J.L. (2017). RNA-binding proteins in neurodegeneration: mechanisms in aggregate. Genes Dev. *31*, 1509–1528.

Delaglio, F., Grzesiek, S., Vuister, G.W., Zhu, G., Pfeifer, J., and Bax, A. (1995). NMRPipe: a multidimensional spectral processing system based on UNIX pipes. J. Biomol. NMR *6*, 277–293.

CellPress
OPEN ACCESS

DeLano, W. (2002). The PyMOL Molecular Graphics System (DeLano Scientific, LLC).

Dimitrova-Paternoga, L., Jagtap, P.K.A., Chen, P.C., and Hennig, J. (2020). Integrative Structural Biology of Protein-RNA Complexes. Structure *28*, 6–28.

Dobin, A., Davis, C.A., Schlesinger, F., Drenkow, J., Zaleski, C., Jha, S., Batut, P., Chaisson, M., and Gingeras, T.R. (2013). STAR: ultrafast universal RNA-seq aligner. Bioinformatics *29*, 15–21.

Dominguez, D., Freese, P., Alexis, M.S., Su, A., Hochman, M., Palden, T., Bazile, C., Lambert, N.J., Van Nostrand, E.L., Pratt, G.A., et al. (2018). Sequence, Structure, and Context Preferences of Human RNA Binding Proteins. Mol. Cell *70*, 854–867.e9.

Dormoy-Raclet, V., Markovits, J., Jacquemin-Sablon, A., and Jacquemin-Sablon, H. (2005). Regulation of Unr expression by 5′- and 3′-untranslated regions of its mRNA through modulation of stability and IRES mediated translation. RNA Biol. *2*, e27–e35.

Drozdetskiy, A., Cole, C., Procter, J., and Barton, G.J. (2015). JPred4: a protein secondary structure prediction server. Nucleic Acids Res. *43* (*W1*), W389–W394.

Duncan, K., Grskovic, M., Strein, C., Beckmann, K., Niggeweg, R., Abaza, I., Gebauer, F., Wilm, M., and Hentze, M.W. (2006). Sex-lethal imparts a sex-specific function to UNR by recruiting it to the msl-2 mRNA 3′ UTR: translational repression for dosage compensation. Genes Dev. *20*, 368–379.

Duncan, K.E., Strein, C., and Hentze, M.W. (2009). The SXL-UNR corepressor complex uses a PABP-mediated mechanism to inhibit ribosome recruitment to msl-2 mRNA. Mol. Cell *36*, 571–582.

El-Gebali, S., Mistry, J., Bateman, A., Eddy, S.R., Luciani, A., Potter, S.C., Qureshi, M., Richardson, L.J., Salazar, G.A., Smart, A., et al. (2019). The Pfam protein families database in 2019. Nucleic Acids Res. *47* (*D1*), D427–D432.

Emsley, P., Lohkamp, B., Scott, W.G., and Cowtan, K. (2010). Features and development of Coot. Acta Crystallogr. D Biol. Crystallogr. *66*, 486–501.

Fenger-Grøn, M., Fillman, C., Norrild, B., and Lykke-Andersen, J. (2005). Multiple processing body factors and the ARE binding protein TTP activate mRNA decapping. Mol. Cell *20*, 905–915.

Fishbein, L., Leshchiner, I., Walter, V., Danilova, L., Robertson, A.G., Johnson, A.R., Lichtenberg, T.M., Murray, B.A., Ghayee, H.K., Else, T., et al.; Cancer Genome Atlas Research Network (2017). Comprehensive Molecular Characterization of Pheochromocytoma and Paraganglioma. Cancer Cell *31*, 181–193.

Franke, D., Petoukhov, M.V., Konarev, P.V., Panjkovich, A., Tuukkanen, A., Mertens, H.D.T., Kikhney, A.G., Hajizadeh, N.R., Franklin, J.M., Jeffries, C.M., and Svergun, D.I. (2017). *ATSAS 2.8*: a comprehensive data analysis suite for small-angle scattering from macromolecular solutions. J. Appl. Cryst. *50*, 1212–1225.

Franken, H., Mathieson, T., Childs, D., Sweetman, G.M.A., Werner, T., Tögel, I., Doce, C., Gade, S., Bantscheff, M., Drewes, G., et al. (2015). Thermal proteome profiling for unbiased identification of direct and indirect drug targets using multiplexed quantitative mass spectrometry. Nat. Protoc. *10*, 1567–1593.

Gaspar, I., Wippich, F., and Ephrussi, A. (2017). Enzymatic production of single-molecule FISH and RNA capture probes. RNA *23*, 1582–1591.

Gáspár, I., Wippich, F., and Ephrussi, A. (2018). Terminal Deoxynucleotidyl Transferase Mediated Production of Labeled Probes for Single-molecule FISH or RNA Capture. Bio-Protococol *8*, e2750.

Gatfield, D., Unterholzner, L., Ciccarelli, F.D., Bork, P., and Izaurralde, E. (2003). Nonsense-mediated mRNA decay in Drosophila: at the intersection of the yeast and mammalian pathways. EMBO J. *22*, 3960–3970.

Gatto, L., and Lilley, K.S. (2012). MSnbase-an R/Bioconductor package for isobaric tagged mass spectrometry data visualization, processing and quantitation. Bioinformatics *28*, 288–289.

Gattoni, R., Mahé, D., Mähl, P., Fischer, N., Mattei, M.G., Stévenin, J., and Fuchs, J.P. (1996). The human hnRNP-M proteins: structure and relation with early heat shock-induced splicing arrest and chromosome mapping. Nucleic Acids Res. *24*, 2535–2542.

Gebauer, F., Corona, D.F.V., Preiss, T., Becker, P.B., and Hentze, M.W. (1999). Translational control of dosage compensation in Drosophila by Sex-lethal: cooperative silencing via the 5′ and 3′ UTRs of msl-2 mRNA is independent of the poly(A) tail. EMBO J. *18*, 6146–6154.

Gerstberger, S., Hafner, M., and Tuschl, T. (2014). A census of human RNA-binding proteins. Nat. Rev. Genet. *15*, 829–845.

Gouet, P., Robert, X., and Courcelle, E. (2003). ESPript/ENDscript: Extracting and rendering sequence and 3D information from atomic structures of proteins. Nucleic Acids Res. *31*, 3320–3323.

Graindorge, A., Carré, C., and Gebauer, F. (2013). Sex-lethal promotes nuclear retention of msl2 mRNA via interactions with the STAR protein HOW. Genes Dev. *27*, 1421–1433.

Graumann, P., Wendrich, T.M., Weber, M.H.W., Schröder, K., and Marahiel, M.A. (1997). A family of cold shock proteins in *Bacillus subtilis* is essential for cellular growth and for efficient protein synthesis at optimal and low temperatures. Mol. Microbiol. *25*, 741–756.

Güntert, P., and Buchner, L. (2015). Combined automated NOE assignment and structure calculation with CYANA. J. Biomol. NMR *62*, 453–471.

Guo, H., Li, Y., Shen, L., Wang, T., Jia, X., Liu, L., Xu, T., Ou, M., Hoekzema, K., Wu, H., et al. (2019). Disruptive variants of CSDE1 associate with autism and interfere with neuronal development and synaptic transmission. Sci. Adv. *5*, eaax2166.

Hansen, H.T., Rasmussen, S.H., Adolph, S.K., Plass, M., Krogh, A., Sanford, J., Nielsen, F.C., and Christiansen, J. (2015). Drosophila Imp iCLIP identifies an RNA assemblage coordinating F-actin formation. Genome Biol. *16*, 123.

Hennig, J., and Sattler, M. (2015). Deciphering the protein-RNA recognition code: combining large-scale quantitative methods with structural biology. BioEssays *37*, 899–908.

Hennig, J., Militti, C., Popowicz, G.M., Wang, I., Sonntag, M., Geerlof, A., Gabel, F., Gebauer, F., and Sattler, M. (2014a). Structural basis for the assembly of the Sxl-Unr translation regulatory complex. Nature *515*, 287–290.

Hennig, J., Gebauer, F., and Sattler, M. (2014b). Breaking the protein-RNA recognition code. Cell Cycle *13*, 3619–3620.

Hentze, M.W., Castello, A., Schwarzl, T., and Preiss, T. (2018). A brave new world of RNA-binding proteins. Nat. Rev. Mol. Cell Biol. *19*, 327–341.

Hooft, R.W.W., Vriend, G., Sander, C., and Abola, E.E. (1996). Errors in protein structures. Nature *381*, 272.

Horos, R., and von Lindern, M. (2012). Molecular mechanisms of pathology and treatment in Diamond Blackfan Anaemia. Br. J. Haematol. *159*, 514–527.

Huber, W., von Heydebreck, A., Sültmann, H., Poustka, A., and Vingron, M. (2002). Variance stabilization applied to microarray data calibration and to the quantification of differential expression. Bioinformatics *18* (*Suppl 1*), S96–S104.

Hughes, C.S., Foehr, S., Garfield, D.A., Furlong, E.E., Steinmetz, L.M., and Krijgsveld, J. (2014). Ultrasensitive proteome analysis using paramagnetic bead technology. Mol. Syst. Biol. *10*, 757.

Jacquemin-Sablon, H., Triqueneaux, G., Deschamps, S., le Maire, M., Doniger, J., and Dautry, F. (1994). Nucleic acid binding and intracellular localization of unr, a protein with five cold shock domains. Nucleic Acids Res. *22*, 2643–2650.

Jiang, W., Hou, Y., and Inouye, M. (1997). CspA, the major cold-shock protein of Escherichia coli, is an RNA chaperone. J. Biol. Chem. *272*, 196–202.

Kabsch, W. (2010). XDS. Acta Crystallogr. D Biol. Crystallogr. *66*, 125–132.

Kay, L.E., Torchia, D.A., and Bax, A. (1989). Backbone dynamics of proteins as studied by 15N inverse detected heteronuclear NMR spectroscopy: application to staphylococcal nuclease. Biochemistry *28*, 8972–8979.

Keller, R. (2004). The Computer Aided Resonance Assignment Tutorial (CANTINA Verlag).

King, M.R., Matzat, L.H., Dale, R.K., Lim, S.J., and Lei, E.P. (2014). The RNA-binding protein Rumpelstiltskin antagonizes gypsy chromatin insulator function in a tissue-specific manner. J. Cell Sci. *127*, 2956–2966.

Kljashtorny, V., Nikonov, S., Ovchinnikov, L., Lyabin, D., Vodovar, N., Curmi, P., and Manivet, P. (2015). The Cold Shock Domain of YB-1 segregates RNA from DNA by non-bonded interactions. PLoS ONE *10*, e0130318.

Laskowski, R.A., Rullmannn, J.A., MacArthur, M.W., Kaptein, R., and Thornton, J.M. (1996). AQUA and PROCHECK-NMR: programs for checking the quality of protein structures solved by NMR. J. Biomol. NMR *8*, 477–486.

Lee, W., Tonelli, M., and Markley, J.L. (2015). NMRFAM-SPARKY: enhanced software for biomolecular NMR spectroscopy. Bioinformatics *31*, 1325–1327.

Lence, T., Akhtar, J., Bayer, M., Schmid, K., Spindler, L., Ho, C.H., Kreim, N., Andrade-Navarro, M.A., Poeck, B., Helm, M., and Roignant, J.Y. (2016). m^6A modulates neuronal functions and sex determination in Drosophila. Nature *540*, 242–247.

Liebschner, D., Afonine, P.V., Baker, M.L., Bunkóczi, G., Chen, V.B., Croll, T.I., Hintze, B., Hung, L.W., Jain, S., McCoy, A.J., et al. (2019). Macromolecular structure determination using X-rays, neutrons and electrons: recent developments in Phenix. Acta Crystallogr. D Struct. Biol. *75*, 861–877.

Lim, C., Lee, J., Choi, C., Kilman, V.L., Kim, J., Park, S.M., Jang, S.K., Allada, R., and Choe, J. (2011). The novel gene twenty-four defines a critical translational step in the Drosophila clock. Nature *470*, 399–403.

Lin, C., Yang, L., Yang, J.J., Huang, Y., and Liu, Z.-R. (2005). ATPase/helicase activities of p68 RNA helicase are required for pre-mRNA splicing but not for assembly of the spliceosome. Mol. Cell. Biol. *25*, 7484–7493.

Linge, J.P., Habeck, M., Rieping, W., and Nilges, M. (2003). ARIA: automated NOE assignment and NMR structure calculation. Bioinformatics *19*, 315–316.

Love, M.I., Huber, W., and Anders, S. (2014). Moderated estimation of fold change and dispersion for RNA-seq data with DESeq2. Genome Biol. *15*, 550.

Madeira, F., Park, Y.M., Lee, J., Buso, N., Gur, T., Madhusoodanan, N., Basutkar, P., Tivey, A.R.N., Potter, S.C., Finn, R.D., and Lopez, R. (2019). The EMBL-EBI search and sequence analysis tools APIs in 2019. Nucleic Acids Res. *47* (*W1*), W636–W641.

Militti, C., Maenner, S., Becker, P.B., and Gebauer, F. (2014). UNR facilitates the interaction of MLE with the lncRNA roX2 during Drosophila dosage compensation. Nat. Commun. *5*, 4762.

Moggridge, S., Sorensen, P.H., Morin, G.B., and Hughes, C.S. (2018). Extending the Compatibility of the SP3 Paramagnetic Bead Processing Approach for Proteomics. J. Proteome Res. *17*, 1730–1740.

Moschall, R., Rass, M., Rossbach, O., Lehmann, G., Kullmann, L., Eichner, N., Strauss, D., Meister, G., Schneuwly, S., Krahn, M.P., and Medenbach, J. (2019). *Drosophila* Sister-of-Sex-lethal reinforces a male-specific gene expression pattern by controlling *Sex-lethal* alternative splicing. Nucleic Acids Res. *47*, 2276–2288.

Nielsen, F.C., Nielsen, J., and Christiansen, J. (2001). A family of IGF-II mRNA binding proteins (IMP) involved in RNA trafficking. Scand. J. Clin. Lab. Invest. Suppl. *234*, 93–99.

Niklasson, M., Otten, R., Ahlner, A., Andresen, C., Schlagnitweit, J., Petzold, K., and Lundström, P. (2017). Comprehensive analysis of NMR data using advanced line shape fitting. J. Biomol. NMR *69*, 93–99.

Okamura, K., Ishizuka, A., Siomi, H., and Siomi, M.C. (2004). Distinct roles for Argonaute proteins in small RNA-directed RNA cleavage pathways. Genes Dev. *18*, 1655–1666.

Patalano, S., Mihailovich, M., Belacortu, Y., Paricio, N., and Gebauer, F. (2009). Dual sex-specific functions of Drosophila Upstream of N-ras in the control of X chromosome dosage compensation. Development *136*, 689–698.

Pereira, B., Billaud, M., and Almeida, R. (2017). RNA-Binding Proteins in Cancer: Old Players and New Actors. Trends Cancer *3*, 506–528.

Pernot, P., Round, A., Barrett, R., De Maria Antolinos, A., Gobbo, A., Gordon, E., Huet, J., Kieffer, J., Lentini, M., Mattenet, M., et al. (2013). Upgraded ESRF BM29 beamline for SAXS on macromolecules in solution. J. Synchrotron Radiat. *20*, 660–664.

Poon, D.K.Y., Withers, S.G., and McIntosh, L.P. (2007). Direct demonstration of the flexibility of the glycosylated proline-threonine linker in the Cellulomonas fimi Xylanase Cex through NMR spectroscopic analysis. J. Biol. Chem. *282*, 2091–2100.

Potter, S.C., Luciani, A., Eddy, S.R., Park, Y., Lopez, R., and Finn, R.D. (2018). HMMER web server: 2018 update. Nucleic Acids Res. *46* (*W1*), W200–W204.

Receveur, V., Czjzek, M., Schülein, M., Panine, P., and Henrissat, B. (2002). Dimension, shape, and conformational flexibility of a two domain fungal cellulase in solution probed by small angle X-ray scattering. J. Biol. Chem. *277*, 40887–40892.

Rieping, W., Habeck, M., Bardiaux, B., Bernard, A., Malliavin, T.E., and Nilges, M. (2007). ARIA2: automated NOE assignment and data integration in NMR structure calculation. Bioinformatics *23*, 381–382.

Ritchie, M.E., Phipson, B., Wu, D., Hu, Y., Law, C.W., Shi, W., and Smyth, G.K. (2015). limma powers differential expression analyses for RNA-sequencing and microarray studies. Nucleic Acids Res. *43*, e47.

Robert, X., and Gouet, P. (2014). Deciphering key features in protein structures with the new ENDscript server. Nucleic Acids Res. *42*, W320–W324.

Rodrigues, J.P., Rode, M., Gatfield, D., Blencowe, B.J., Carmo-Fonseca, M., and Izaurralde, E. (2001). REF proteins mediate the export of spliced and unspliced mRNAs from the nucleus. Proc. Natl. Acad. Sci. USA *98*, 1030–1035.

Sachs, R., Max, K.E.A., Heinemann, U., and Balbach, J. (2012). RNA single strands bind to a conserved surface of the major cold shock protein in crystals and solution. RNA *18*, 65–76.

Sanders, S.J., Murtha, M.T., Gupta, A.R., Murdoch, J.D., Raubeson, M.J., Willsey, A.J., Ercan-Sencicek, A.G., DiLullo, N.M., Parikshak, N.N., Stein, J.L., et al. (2012). De novo mutations revealed by whole-exome sequencing are strongly associated with autism. Nature *485*, 237–241.

Sasse, A., Laverty, K.U., Hughes, T.R., and Morris, Q.D. (2018). Motif models for RNA-binding proteins. Curr. Opin. Struct. Biol. *53*, 115–123.

Sattler, M., Schleucher, J., and Griesinger, C. (1999). Heteronuclear multidimensional NMR experiments for the structure determination of proteins in solution employing pulsedfield gradients. Progress in Nuclear Magnetic Resonance Spectroscopy2nd, *34* (Science Direct), pp. 93–202.

Savitski, M.M., Wilhelm, M., Hahne, H., Kuster, B., and Bantscheff, M. (2015). A Scalable Approach for Protein False Discovery Rate Estimation in Large Proteomic Data Sets. Mol. Cell. Proteomics *14*, 2394–2404.

Schilling, F., Warner, L.R., Gershenzon, N.I., Skinner, T.E., Sattler, M., and Glaser, S.J. (2014). Next-generation heteronuclear decoupling for high-field biomolecular NMR spectroscopy. Angew. Chem. Int. Ed. Engl. *53*, 4475–4479.

Schneider, T., Hung, L.-H., Aziz, M., Wilmen, A., Thaum, S., Wagner, J., Janowski, R., Müller, S., Schreiner, S., Friedhoff, P., et al. (2019). Combinatorial recognition of clustered RNA elements by the multidomain RNA-binding protein IMP3. Nat. Commun. *10*, 2266.

Shen, Y., Delaglio, F., Cornilescu, G., and Bax, A. (2009). TALOS+: a hybrid method for predicting protein backbone torsion angles from NMR chemical shifts. J. Biomol. NMR *44*, 213–223.

Simon, B., and Köstler, H. (2019). Improving the sensitivity of FT-NMR spectroscopy by apodization weighted sampling. J. Biomol. NMR *73*, 155–165.

Singh, G., Pratt, G., Yeo, G.W., and Moore, M.J. (2015). The Clothes Make the mRNA: Past and Present Trends in mRNP Fashion. Annu. Rev. Biochem. *84*, 325–354.

Sreerama, N., and Woody, R.W. (1993). A self-consistent method for the analysis of protein secondary structure from circular dichroism. Anal. Biochem. *209*, 32–44.

Sreerama, N., and Woody, R.W. (2004). On the analysis of membrane protein circular dichroism spectra. Protein Sci. *13*, 100–112.

Sridharan, S., Kurzawa, N., Werner, T., Günthner, I., Helm, D., Huber, W., Bantscheff, M., and Savitski, M.M. (2019). Proteome-wide solubility and thermal stability profiling reveals distinct regulatory roles for ATP. Nat. Commun. *10*, 1155.

Strimmer, K. (2008). fdrtool: a versatile R package for estimating local and tail area-based false discovery rates. Bioinformatics *24*, 1461–1462.

Svergun, D., Barberato, C., and Koch, M.H.J. (1995). *CRYSOL* – a Program to Evaluate X-ray Solution Scattering of Biological Macromolecules from Atomic Coordinates. J. Appl. Crystallogr. *28*, 768–773.

CellPress
OPEN ACCESS

Szklarczyk, D., Gable, A.L., Lyon, D., Junge, A., Wyder, S., Huerta-Cepas, J., Simonovic, M., Doncheva, N.T., Morris, J.H., Bork, P., et al. (2019). STRING v11: Protein-Protein Association Networks With Increased Coverage, Supporting Functional Discovery in Genome-Wide Experimental Datasets. Nucleic Acids Res. https://doi.org/10.1093/nar/gky1131.

Szostak, E., García-Beyaert, M., Guitart, T., Graindorge, A., Coll, O., and Gebauer, F. (2018). Hrp48 and eIF3d contribute to msl-2 mRNA translational repression. Nucleic Acids Res. *46*, 4099–4113.

Terwilliger, T.C., Adams, P.D., Read, R.J., McCoy, A.J., Moriarty, N.W., Grosse-Kunstleve, R.W., Afonine, P.V., Zwart, P.H., and Hung, L.W. (2009). Decision-making in structure solution using Bayesian estimates of map quality: the PHENIX AutoSol wizard. Acta Crystallogr. D Biol. Crystallogr. *65*, 582–601.

Trewhella, J., Duff, A.P., Durand, D., Gabel, F., Guss, J.M., Hendrickson, W.A., Hura, G.L., Jacques, D.A., Kirby, N.M., Kwan, A.H., et al. (2017). 2017 publication guidelines for structural modelling of small-angle scattering data from biomolecules in solution: an update. Acta Crystallogr. D Struct. Biol. *73*, 710–728.

Triqueneaux, G., Velten, M., Franzon, P., Dautry, F., and Jacquemin-Sablon, H. (1999). RNA binding specificity of Unr, a protein with five cold shock domains. Nucleic Acids Res. *27*, 1926–1934.

Voorhees, R.M., and Ramakrishnan, V. (2013). Structural basis of the translational elongation cycle. Annu. Rev. Biochem. *82*, 203–236.

Vranken, W.F., Boucher, W., Stevens, T.J., Fogh, R.H., Pajon, A., Llinas, M., Ulrich, E.L., Markley, J.L., Ionides, J., and Laue, E.D. (2005). The CCPN data model for NMR spectroscopy: development of a software pipeline. Proteins *59*, 687–696.

Wan, R., Bai, R., and Shi, Y. (2019). Molecular choreography of pre-mRNA splicing by the spliceosome. Curr. Opin. Struct. Biol. *59*, 124–133.

Wang, M., Ly, M., Lugowski, A., Laver, J.D., Lipshitz, H.D., Smibert, C.A., and Rissland, O.S. (2017). ME31B globally represses maternal mRNAs by two distinct mechanisms during the *Drosophila* maternal-to-zygotic transition. eLife *6*, e27891.

Werner, T., Sweetman, G., Savitski, M.F., Mathieson, T., Bantscheff, M., and Savitski, M.M. (2014). Ion coalescence of neutron encoded TMT 10-plex reporter ions. Anal. Chem. *86*, 3594–3601.

Wickham, H. (2009). ggplot2 (Springer New York).

Wilhelm, J.E., Buszczak, M., and Sayles, S. (2005). Efficient protein trafficking requires trailer hitch, a component of a ribonucleoprotein complex localized to the ER in Drosophila. Dev. Cell *9*, 675–685.

Wilkinson, M.E., Lin, P.-C., Plaschka, C., and Nagai, K. (2018). Cryo-EM Studies of Pre-mRNA Splicing: From Sample Preparation to Model Visualization. Annu. Rev. Biophys. *47*, 175–199.

Williamson, M.P. (2013). Using chemical shift perturbation to characterise ligand binding. Prog. Nucl. Magn. Reson. Spectrosc. *73*, 1–16.

Wurth, L., Papasaikas, P., Olmeda, D., Bley, N., Calvo, G.T., Guerrero, S., Cerezo-Wallis, D., Martinez-Useros, J., García-Fernández, M., Hüttelmaier, S., et al. (2016). UNR/CSDE1 Drives a Post-transcriptional Program to Promote Melanoma Invasion and Metastasis. Cancer Cell *30*, 694–707.

Xia, K., Guo, H., Hu, Z., Xun, G., Zuo, L., Peng, Y., Wang, K., He, Y., Xiong, Z., Sun, L., et al. (2014). Common genetic variants on 1p13.2 associate with risk of autism. Mol. Psychiatry *19*, 1212–1219.

Yang, X.J., Zhu, H., Mu, S.R., Wei, W.J., Yuan, X., Wang, M., Liu, Y., Hui, J., and Huang, Y. (2019a). Crystal structure of a Y-box binding protein 1 (YB-1)-RNA complex reveals key features and residues interacting with RNA. J. Biol. Chem. *294*, 10998–11010.

Yang, Y., Wang, L., Han, X., Yang, W.L., Zhang, M., Ma, H.L., Sun, B.F., Li, A., Xia, J., Chen, J., et al. (2019b). RNA 5-Methylcytosine Facilitates the Maternal-to-Zygotic Transition by Preventing Maternal mRNA Decay. Mol. Cell *75*, 1188–1202.e11.

Zhu, G., Xia, Y., Nicholson, L.K., and Sze, K.H. (2000). Protein dynamics measurements by TROSY-based NMR experiments. J. Magn. Reson. *143*, 423–426.

Zou, F., Tu, R., Duan, B., Yang, Z., Ping, Z., Song, X., Chen, S., Price, A., Li, H., Scott, A., et al. (2020). *Drosophila* YBX1 homolog YPS promotes ovarian germ line stem cell development by preferentially recognizing 5-methylcytosine RNAs. Proc. Natl. Acad. Sci. USA *117*, 3603–3609.

STAR★METHODS

KEY RESOURCES TABLE

REAGENT or RESOURCE	SOURCE	IDENTIFIER
Antibodies		
Rabbit polyclonal antibody against Drosophila Unr	Generated in-house in Dr. Gebauer laboratory	N/A
Mouse monoclonal antibody against V5	Invitrogen	RRID: AB_2556564
Mouse monoclonal antibody against a-tubulin	Sigma	T9026-100UL; RRID: AB_477593
Bacterial and Virus Strains		
E. coli chemically competent BL21(DE3)	ThermoFisher	C600003
E. coli chemically competent Dh5a-T1R	ThermoFisher	12297016
Chemicals, Peptides, and Recombinant Proteins		
Anti-FLAG M2 magnetic beads	Sigma	M8823-1ML
Schneider's Drosophila medium	ThermoFisher	21720001
FBS	ThermoFisher	10270106
Effectene	QIAGEN	301425
Pierce ECL Western Blotting Substrate	ThermoFisher	32209
RNase OUT	ThermoFisher	10777019
pCp-Cy5	Jena Bioscience	NU-1706-Cy5
Pierce Protein A/G Magnetic Beads	ThermoFisher	88803
Sequencing Grade modified trypsin	Promega	V5111
TMT10plex	ThermoFisher	90110
AAA AAA AUG RNA oligomer	Biomers	N/A
AAA AAA AAA AAA AAA RNA oligomer	Biomers	N/A
Critical Commercial Assays		
BCA Assay Kit	ThermoFisher	23227
Dual Luciferase Assay System	Promega	E1910
Megascript T7 kit	ThermoFisher	AM1334
Galacto Star (Tropix)	ThermoFisher	T1012
Renilla-Glo® Luciferase Assay System	Promega	E2710
Turbo DNA free Kit	ThermoFisher	AM1907
SuperScript II reverse transcriptase	ThermoFisher	18064014
NEBNext Ultra II Directional RNA Library Prep Kit for Illumina	NEB	E7760
Pierce Silver Stain Kit	ThermoFisher	24612
Deposited Data		
Structure of *drosophila* Unr CSD12	This paper	PDB: 6Y6M
Structure of *drosophila* Unr CSD456	This paper	PDB: 6Y6E
Structure of *drosophila* Unr CSD78	This paper	PDB: 6Y4H
Structure of *drosophila* Unr CSD9	This paper	PDB: 6Y96
NMR data of *drosophila* Unr CSD12	This paper	BMRB: 34493
NMR data of *drosophila* Unr CSD456	This paper	BMRB: 28088
NMR data of *drosophila* Unr CSD78	This paper	BMRB: 34492
NMR data of *drosophila* Unr CSD789	This paper	BMRB: 28086
NMR data of *drosophila* Unr CSD9	This paper	BMRB: 34498
SAXS data of *drosophila* Unr CSD456	This paper	SASBDB: SASDHJ7
SAXS data of *drosophila* Unr CSD78	This paper	SASBDB: SASDHK7

(*Continued on next page*)

Continued

REAGENT or RESOURCE	SOURCE	IDENTIFIER
Proteomics data	This paper	ProteomeXchange: PXD018115
RIP-seq data	This paper	https://www.ebi.ac.uk/ena: PRJEB37467
Experimental Models: Cell Lines		
Drosophila melanogaster: Schneider's Drosophila Line 2	ATCC	ATCC CRL-1963
Oligonucleotides		
Primers for RNAi and qPCR primers, see Table S5	Specific for each primer, check Table S5	N/A
Software and Algorithms		
XDS	Kabsch, 2010	SBGrid Consortium
Phenix	Liebschner et al., 2019	SBGrid Consortium
Coot	Emsley et al., 2010	SBGrid Consortium
NMRPipe	Delaglio et al., 1995	SBGrid Consortium
Cara	Keller, 2004	SBGrid Consortium
CYANA 3.98	Güntert and Buchner, 2015	http://www.cyana.org/wiki/index.php/Main_Page
Talos	Shen et al., 2009	SBGrid Consortium
ARIA 1.2	Rieping et al., 2007	SBGrid Consortium
PROCHECK	Laskowski et al., 1996	SBGrid Consortium
WHATCHECK	Hooft et al., 1996	SBGrid Consortium
NMRFAM-Sparky	Lee et al., 2015	SBGrid Consortium
CcpNMR	Vranken et al., 2005	SBGrid Consortium
PINT	Niklasson et al., 2017	https://pint-nmr.github.io/PINT/
ATSAS 2.7.1	Franke et al., 2017	SBGrid Consortium
STAR aligner 2.7.1a	Dobin et al., 2013	https://github.com/alexdobin/STAR
RStudio	RStudio	SBGrid Consortium
Inkscape	Inkscape developers, GNU	https://inkscape.org/
GraphPad Prism 5	GraphPad software Inc.	https://www.graphpad.com/scientific-software/prism/
PyMol 1.8.2.3	Schrödinger, LLC	https://pymol.org/2/
Gnuplot 4	Gnuplot	http://www.gnuplot.info

RESOURCE AVAILABILITY

Lead Contact

Further information and requests for resources should be directed to and will be fulfilled by the Lead Contact, Janosch Hennig (janosch.hennig@embl.de).

Materials Availability

This study did not generate new unique reagents.

Data and Code Availability

The high resolution structures of the different protein constructs generated in this study are available at the PDB (*Drosophila* Unr CSD12: 6Y6M; *Drosophila* Unr CSD456: 6Y6E; *Drosophila* Unr CSD78: 6Y4H; *Drosophila* Unr CSD9: 6Y96), the NMR assignments at the BMRB (*Drosophila* Unr CSD12: 34493; *Drosophila* Unr CSD456: 28088; *Drosophila* Unr CSD78: 34492; *Drosophila* Unr CSD789: 28086; *Drosophila* Unr CSD9: 34498) and the SAXS data at the SASBDB (*Drosophila* Unr CSD456: SASDHJ7; *Drosophila* Unr CSD78: SASDHK7).

The proteomics datasets generated and analyzed during this study are available at ProteomeXchange (PXD018115) and the RIP-seq datasets at https://www.ebi.ac.uk/ena (PRJEB37467). This study did not generate codes.

EXPERIMENTAL MODEL AND SUBJECT DETAILS

Bacterial Strains and Culture Conditions to generate plasmids and express protein

E. coli DH5 α (*fhuA2 lac(del)U169 phoA glnV44 Φ80' lacZ(del)M15 gyrA96 recA1 relA1 endA1 thi-1 hsdR17*) was used to generate plasmids that were cloned in this study. The cells were grown in LB medium at 37°C and harvested after overnight cultures.

E. coli BL21 (DE3) cells (*E. coli B dcm ompT hsdS*($r_B^- m_B^-$) *gal*) were used to express the different recombinant proteins. The cells were grown at 37°C. For NMR spectroscopy expression was conducted in isotope labeled M9 minimal medium in H_2O or D_2O (for backbone assignments of CSD456 and CSD789), supplemented with $^{15}NH_4Cl$ and/or ^{13}C-glucose as sole nitrogen and carbon source (isotopes were purchased from Cambridge Isotope Laboratories). Proteins that were used for other purposes than NMR were expressed in TB medium. The cultures were induced with 0.2 mM IPTG at an OD_{600} of 0.8 for minimal and 1.2 for TB media and grown over night at 17°C.

Culturing of Schneider's *Drosophila* Line 2 (SL2)

SL2 cells (ATCC CRL-1963; male) were kept in culture at 25°C in Schneider's Medium with penicillin/streptomycin (1% v/v) and 10% FBS.

METHOD DETAILS

Plasmids

Plasmids for the expression of *Drosophila* Unr CSD12 (R186-V344), CSD123 (R186-L414), CSD456 (E422-H677), CSD6 (F593-H677), CSD78 (A756-K922), CSD789 (A756-K922), CSD8 (P840-K922) and CSD9 (G911-D990) (UniprotKB: Q9VSK3) and all other 111 tested constructs (Table S1) were derived from pETM11 (derived from pBR322; G. Stier) and comprise a His_6- affinity tag connected via a tobacco etch virus protease (TEV)-cleavage site to Unr.

For *in vitro* translation assays Unr full-length was cloned into a pET15b-derived MS2 fusion vector, to express N-terminal His_6-MS2 fusion proteins. For SL2 cell culture experiments Unr full-length was cloned into a pAc5.1B vector, which contains a C-terminal His_6 and V5 tag.

The protein constructs were cloned using the restriction free cloning approach. Point mutations were inserted by site directed mutagenesis (Braman et al., 1996).

The msl2 promotor-constructs (msl2-FC-bGal (Graindorge et al., 2013) and BmutLMS2 (Abaza and Gebauer, 2008)) were used as described before.

Protein Purification

After expression of the proteins, the harvested cells were resuspended in 50 mM HEPES/NaOH pH 8.0, 500 mM NaCl, 1.4 mM β-mercaptoethanol, 30 mM imidazole, (and 1M urea for full-length Unr) and lysed using a French press. The cleared lysate was applied to a 5 mL Nickel-nitrilotriacetic acid (Ni-NTA) column (Macherey-Nagel) and after washing with 10 column volumes (CVs) lysis buffer, the protein was eluted by increasing the imidazole concentration to 500 mM. Except for MS2 tagged full-length Unr constructs, all proteins were cleaved with TEV-protease and dialyzed overnight at 4°C against 10 mM imidazole and 150 mM NaCl using dialysis tubes with cut-offs between 3.5-10 kDa. After passing through a second Ni-NTA column, constructs, that include CSD1 were injected on a 5ml FF Heparin column (GE) and eluted with a 2 M salt buffer to remove unspecifically bound bacterial RNAs. In a last step all proteins (except the His_6 tagged full-length Unr constructs, which were only buffer exchanged on a HiPrep 26/10 Desalting column (GE)) were purified and buffer exchanged via size-exclusion chromatography on a S75 gel-filtration column (GE) and concentrated to desired values using Amicon Ultra Centrifugal Filters with respective cut-off sizes (Merck-Millipore).

NMR sample buffer was 20 mM NaP (pH 6.5), 50 mM NaCl, 1 mM DTT (10 mM for CSD12), 10% D_2O and 0.01% NaN_3), whereas proteins for crystallization, SAXS and EMSA were prepared using 25 mM HEPES/NaOH pH 7.5, 150 mM NaCl, 1 mM DTT and 0.01% NaN_3. The MS2 tagged full-length Unr constructs, that were used for *in vitro* translation assays were, after the first Ni-NTA column, further purified using Anti-FLAG M2 magnetic beads according to the manufactures protocol and finally dialyzed against a buffer containing 20 mM HEPES/NaOH pH 7.4, 20% Glycerol, 1 mM DTT, 0.01% NP-40 and 0.2 mM EDTA.

Protein quality was assessed by Coomassie staining and protein quantity was assessed by using NanoDrop or BCA Assay Kit for CSD12, CSD123, CSD6, CSD8 and CSD9.

Crystal structure determination

CSD456 was concentrated to 20 mg/ml. The crystals have grown in 0.1 M tri-sodium citrate at pH 5.5 and 20% PEG3000 at room temperature to a size of about 0.7x0.2x0.2 mm size without any visible macroscopic defects. For heavy atom soaking, crystals were left in 0.1 mM $(C_2H_5HgO)_2HPO_2$ over night at room temperature. Before freezing, crystals were soaked in mother liquor supplemented with 40% glycerol as a cryoprotectant and multiwavelength anomalous diffraction (MAD) datasets were collected at the ID29 beamline of the European Synchrotron Radiation Facility (ESRF), Grenoble, France. Heavy atom-soaked crystals diffracted up to 2.2 Å. Data was processed in XDS (Kabsch, 2010) and phasing and initial automated model building was performed using AutoSol from

the Phenix suite (Liebschner et al., 2019; Terwilliger et al., 2009). The resulting structure was further refined with several rounds of model building in COOT (Emsley et al., 2010) and refinement in the Phenix suite. Structural statistics are listed in Table S3.

NMR spectroscopy

All NMR measurements were performed at 298 K on Bruker Avance III NMR spectrometers with magnetic field strengths corresponding to proton Larmor frequencies of 600 MHz, 700 MHz or 800 MHz equipped with a cryogenic triple resonance gradient probe head (600 and 800 MHz), or a room temperature triple resonance probe head (700 MHz). NMR sample concentrations for acquiring experiments necessary for structure calculation (backbone and sidechain assignment experiments, as well as 3D-NOESY-type experiments) were 0.5 mM for CSD78 and 0.3 mM for CSD12 with RNA (AAA AAA AUG) in 1.2x molar excess (to improve sample stability). For backbone assignments of CSD456 and 789, samples with a concentration of 0.3 and 0.5 mM, respectively were used. Experiments for backbone assignments have been performed on ^{13}C,^{15}N-labeled samples (using 70% D_2O in growth medium for CSD456 and CSD789) using conventional triple-resonance experiments (HNCO, HNCA, CBCA(CO)NH, HN(CO)CA and HNCACB) (Sattler et al., 1999). Side chain assignments were done using HBHA(CO)NH, HCCH-TOCSY, and CCH-TOCSY spectra. 3D ^{13}C-NOESY-HMQC and ^{15}N- NOESY-HSQC spectra with 100 ms mixing times and 3D (H)CCH HMQC-NOESY-HMQC, 3D HCH NOESY-HMQC and (H)CNH HMQC-NOESY-HSQC spectra with 70 ms mixing times were used for side chain and NOE assignments to derive distance restraints. 3D HMQC-based spectra were recorded in D_2O with a decoupling scheme as described in (Schilling et al., 2014). All spectra were acquired using the apodization weighted sampling scheme (Simon and Köstler, 2019) and processed using NMRPipe (Delaglio et al., 1995). Resonance assignments were done with the program Cara (Keller, 2004).

CYANA 3.98 (Güntert and Buchner, 2015) was used for NOE-based structure calculation. Dihedral angle restraints were derived from backbone chemical shifts, using the program TALOS (Shen et al., 2009). A final water refinement was done using ARIA 1.2 (Linge et al., 2003; Rieping et al., 2007). Structure validation of the final ensemble of 20 structures with lowest energies was done using PROCHECK and WHATCHECK (Hooft et al., 1996; Laskowski et al., 1996). The structural statistics are shown in Table S2.

For NMR-based RNA titrations, a protein concentration of 0.1 mM was used for CSD6, CSD8, CSD9 and CSD78. CSD12, CSD456 and CSD789 were titrated at a concentration of 0.2 mM. The 15N labeled proteins were titrated with various ratios of a purchased RNA oligonucleotide (AAA AAA AUG), and a ^{1}H,^{15}N HSQC was recorded for each titration point. Further for CSD789 a deuterated protein sample was used to titrate an A15-mer RNA oligonucleotide. As the RNA stock solution was highly concentrated (10 mM), the dilution effect was negligible but still taken into account. Titration data was analyzed using Sparky (Lee et al., 2015) and chemical shift perturbations δ (ppm) at saturation were calculated according to: $\delta(\text{ppm}) = \sqrt{(\Delta H)^2 + (0.2 * \Delta N)^2}$ (Williamson, 2013). CCP was used to determine the dissociation constants by fitting the chemical shift perturbations versus the RNA concentration of residues which shift at a protein:RNA ratio of 1:2 more than the average plus the standard deviation of all measured shifts using $A(B + x - \sqrt{((B+x)^2 + 4x)})$ as a fitting function (Vranken et al., 2005).

R1, R2 and 1H-15N heteronuclear NOE experiments were acquired using standard pulse sequences (Kay et al., 1989; Zhu et al., 2000). Relaxation delays for R_2 and R_1 were chosen dependent on the size of the protein (CSD78, R_1: relaxation delays of 1600, 20, 1300, 50, 800, 100, 500, 250, 650, 150, 1000, 400, 50 and 500 ms, R_2: 16, 128, 192, 48, 80, 160, 32, 112, 64, 96, 144, 16, 80 and 160 ms, CSD12, R_1: 2000, 50, 100, 700, 300, 400, 200, 1000, 150, 500, 1600 and 50 ms, R_2:16, 132, 64, 32, 50, 100, 116, 166, 200, 16, 132 and 64 ms)

PINT (Ahlner et al., 2013; Niklasson et al., 2017) was used for the analysis of peak integration and data fitting to derive spin relaxation parameters from which the rotational correlation time (τ_c) was calculated for each construct according to (Kay et al., 1989).

SAXS data acquisition and analysis

SAXS statistics are listed in Table S4 according to community guidelines (Trewhella et al., 2017). The proteins were measured in the BioSAXS beamline BM29 (Pernot et al., 2013) at the ESRF, Grenoble, using an X-ray wavelength of 0.992 Å. For the measurements 30 µL of protein sample or buffer were purged through a quartz capillary at 25°C, while 10 frames with 0.5 s exposure time per frame were collected using a Pilatus 1M detector. Each individual frame was checked for radiation damage and all frames without damage were merged. The buffer was measured before and after each sample and its contribution was subtracted from the merged datasets of the protein samples. A Guinier analysis was carried out to assess data quality. The data were analyzed using the data analysis software package ATSAS 2.7.1 (Franke et al., 2017). CRYSOL calculations were done using the default settings (Svergun et al., 1995).

Electrophoretic mobility-shift assays (EMSA)

SL67 RNA that was used for the EMSAs (5′-ACAAUAUGCAAUACAAUACAAUACAAGACAAAAAAAUGUGUCUUGGAACCAA CAUUGUACAAGUCGCAAUGCAAACUGAAGUCUUAAAAGACGUGUAAAAUGUUGCAAAUUAAGCAAAUAUAUAUGCAUAUAUGG GUAACGUUUUACGCGCCUUAACCAGU-3’) was prepared by T7 *in vitro* transcription using unlabeled rNTPs and a template which was cloned into pUC19 plasmid DNA and contained a hammerhead ribozyme (HH) cleavage site (in *cis*) at the 5′ end and a VS (Varkud satellite) ribozyme recognition sequence at the 3′ end (for cleavage in *trans*). After transcription, proteins were removed by phenol/chloroform extraction. The RNA was purified by denaturing 12% PAGE and extracted from the gel by electro-elution. The final sample was concentrated and dialyzed against 20 mM NaPi, pH 6.5 buffer

All RNA-binding reactions were performed in a binding buffer containing 20 mM HEPES/NaOH pH 7.5, 50 mM NaCl, 10% [v/v] glycerol and 2 mM DTT. Reactions were equilibrated for 1 hr at 4°C. Next, the samples were resolved in a 6% native polyacrylamide gel in 0.5xTBE at 4°C for 3-4 hours. Each reaction contained a fluorescently labeled probe (~25 nM RNA) which was obtained by 3′ end labeling with T4 NA ligase, ATP and pCp-Cy5. The gels were imaged with a Typhoon Trio imager (GE Healthcare).

Protein melting temperature

Protein melting temperature was determined using nanoDSF technology (nanotemper) and the intrinsic tryptophan fluorescence of some Unr constructs. Proteins were soaked into the capillary and heated up 1°C/min. Depending on the protein concentration the excitation varied from 10%–30%. The data analysis was done with provided software, and the temperature at which 50% of the protein is unfolded was taken as melting temperature.

Circular dichroism

The proteins were dialyzed into a buffer containing 20mM HEPES/NaOH pH 7.5, 20 mM NaCl and 1 mM DTT for circular dichroism (CD) measurements. The measurements were done at 10 μM concentration in a 0.2 mm cuvette at 20°C, using a Jasco J-815 CD spectrometer. The wavelength range was 240 to 190 nm, measured with 0.1 nm steps, and averaged over 5 points per wavelength. Analysis was done using SELCON3 to calculate the secondary structure content (Sreerama and Woody, 1993, 2004).

In vitro translation assay

The mRNAs were *in vitro* transcribed from a linearized vector using a T3 polymerase (Gebauer et al., 1999). All mRNAs contained a 5′ M7GpppG cap and a poly(A) tail of 73 nucleotides. After *in vitro* transcription all RNAs were purified using G_{50} desalting columns (GE) following the manufactures protocol and a phenol/chloroform extraction, and their quality was assessed in agarose gels.

The *in vitro* translation reactions in *Drosophila* embryo extracts were performed in a final volume of 12.5 μl, as described previously (Gebauer et al., 1999), with a final concentration of 60 μM amino acids, 0.6 mM DTT, 24 mM HEPES/KOH pH7.4, 0.26 mM $Mg(OAc)_2$, 48 mM KOAc, 16.8 mM creatin phosphate, 80 ng/μl creatin kinase, 0.4 ng/μl Renilla mRNA and 1.6 ng/μl BmutL-MS2. Further, increasing amounts of full-length *Drosophila*-MS2 tagged Unr, or mutated versions of it, were added prior to incubation.

The translation efficiency was measured using the Dual Luciferase Assay System. The Renilla values were used as an internal control to correct the Firefly expression.

RNAi, transfection and reporter gene assay

RNAi was performed in 6 well dishes as described earlier (Duncan et al., 2006). Briefly, 2x106 SL2 cells per well were pelleted and resuspended in 1 mL Schneider's medium without FBS. 15 ug/ml of dsRNA against the 3′UTR of the endogenous Unr or GFP as a control were added directly to each well. After 40 min, 1 mL of 20% supplemented FBS Schneider's medium was added. The dsRNAs were *in vitro* transcribed from amplified template DNA strands using the Megascript T7 kit. Oligonucleotides used to amplify dsRNAs are listed in the Table S7. The cells were transfected with Effectene according to the recommendations three days after the knock-down. 2 ng of pAC-SXL, 75 ng of the reporter gene construct, 100 ng of pAC-V5-Unr, 10 ng pf pAC-Renilla and 163 ng of an empty pAC vector from endotoxin free isolated DNA were used per reaction. β-gal activity was measured with Galacto-Star and Rluc activity with Renilla substrate, both according to recommendations. The luminescence activities were later normalized against mRNA levels of β-gal and RLuc obtained by RT-qPCR. qPCR was performed using SYBR Green on Applied Biosystems 7000 and the used primers are listed in the Supplemental Material. RNA was extracted using Trizol reagent and the DNA was digested using the Turbo DNase Kit according to manufacturer's protocol. Reverse transcription was done using the SuperScript II reverse transcriptase according to manufacturer's protocol. A western blot was done to assess the quality of the knock-down and the transfection efficiency of Unr. After blotting the gel on a nitrocellulose membrane and blocking in 5% milk in PBS-T for 2h at room temperature, the primary antibodies were added and incubated overnight in the cold room under agitation. For detection of Unr a polyclonal antibody serum against amino acids 1-156 of Unr (1:2000) and a monoclonal anti-V5 antibody (1:1000) were used. Tubulin was used as a loading control and detected by a monoclonal anti-tubulin antibody (1:2000). The blots were incubated with a secondary HRP-linked antibody afterward and developed using an ECL substrate.

Unr immunoprecipitation

For Unr immunoprecipitation, endogenous Unr was knocked down to liberate rate-limiting targets and the SL2 cells were transfected with 2 μg pAC-Unr wild-type and mutants (456 ID and 456-78 ID) and an empty pAC vector as described before in a 10 cm dish per reaction. UV crosslinking was done three days after the transfection in a thin layer of ice-cold PBS at 300 mJ/cm^3 to stabilize transient and weak RNA protein interactions. For cell lysis, cells were sonicated in 20 mM HEPES/NaOH pH 7.5, 100mM NaCl, 1mM $MgCl_2$, 0.05% NP-40 and 40U/ml RNaseOUT for 3 cycles of 30 s at a low energy level using a Bioruptor (Diagenode). Afterward the cleared lysate of the wild-type, mutant and empty vector transfected cells was incubated with 1 μg of V5 antibody per 3 mg of total protein and incubated at 4°C for 2h. The total protein concentration was determined earlier by a BCA assay. After incubation, 4 μL of magnetic protein A/G beads per 1 μg of used antibody was added and incubated for another 10 minutes. To get rid of unspecific binding the samples were washed with 1 mL 20 mM HEPES/NaOH pH 7.5, 150 mM NaCl, 1 mM EDTA, 0.5% NP-40, 0.5 mM DTT, 40U/ml RNaseOUT and 1% Triton-X for three times, before the beads were resuspended in the final buffer and volume. In case of the RNase

treated samples, 250U of benzonase were added during washing steps. For RIP-Seq experiments samples were resuspended in 125 μL of 20 mM HEPES/NaOH pH 7.5 and 150 mM NaCl and IP-MS samples were resuspended in 30 μL 20 mM HEPES/NaOH pH 7.5, 150 mM NaCl and 10% SDS.

RNA sequencing and data analysis

The immunoprecipitated samples were incubated with 0.2 mg/ml Proteinase K for 30 min at 55°C. Afterward 400 μL Trizol were added and the RNA was extracted according to recommendations. Finally, the RNA was resuspended in 10 μL water and the ribosomal RNA was depleted using an approach of fishing for ribosomal RNA with biotinylated oligonucleotides (Gaspar et al., 2017; Gáspár et al., 2018). After checking the RNA depletion on a Bioanalyzer (Agilent) a barcoded stranded cDNA library was generated using the NEBNext Ultra II Directional RNA Library Prep Kit for Illumina. Obtained libraries that passed the QC step were pooled in equimolar amounts; 1.9 pM solution of this pool was loaded on the Illumina sequencer NextSeq 500 and sequenced uni-directionally, generating ∼500 million reads, each 85 bases long. The alignment of the sequencing reads was done using STAR aligner version 2.7.1a to a genome reference of *Drosophila melanogaster* BDGP6.22.97 from ENSEMBL (Dobin et al., 2013). The read counts were obtained using in-built implementation of HTSeq-count in STAR aligner with the '–quantMode GeneCounts' option (Table S6). Finally the analysis of triplicate samples to generate PCA plots and assess differentially expressed genes was done in R v3.5.1 using DESeq2 v1.20.0 (Love et al., 2014). The EnhancedVolcano package v1.3.5 was used to generate the volcano plots (Blighe et al., 2019). To tweak the appearance of the resulting figures ggplot2 was used (Wickham, 2009). The VennDiagram package v1.6.20 was used to generate the Venn diagrams (Chen and Boutros, 2018).

Sample preparation and LC-MS/MS analysis of IP-MS samples

The immunoprecipitated samples of control, wild-type and mutant in the absence and presence or RNase were incubated for 5 min at 95°C and subjected to an in-solution tryptic digest using a modified version of the Single-Pot Solid-Phase-enhanced Sample Preparation (SP3) protocol (Hughes et al., 2014; Moggridge et al., 2018). In total three biological replicates were prepared including control, wild-type and mutant derived lysates (n = 3). To check the pull down efficiency a TGX 4%–20% gradient polyacrylamide gel was silver stained using the Pierce Silver Stain kit according to manufacturer's protocol. The lysates were added to Sera-Mag Beads in 10 μl 15% formic acid and 30 μl of ethanol. Binding of proteins was achieved by shaking for 15 min at room temperature. SDS was removed by 4 subsequent washes with 200 μl of 70% ethanol. Proteins were digested overnight at room temperature with 0.4 μg of sequencing grade modified trypsin in 40 μl HEPES/NaOH, pH 8.4 in the presence of 1.25 mM TCEP and 5 mM chloroacetamide. Beads were separated, washed with 10 μl of an aqueous solution of 2% DMSO and the combined eluates were dried down. Peptides were reconstituted in 10 μl of H2O and reacted for 1 h at room temperature with 80 μg of TMT10plex (Werner et al., 2014) label reagent dissolved in 4 μl of acetonitrile. Excess TMT reagent was quenched by the addition of 4 μl of an aqueous 5% hydroxylamine solution. Peptides were reconstituted in 0.1% formic acid, mixed to achieve a 1:1 ratio across all TMT-channels and purified by a reverse phase clean-up step (OASIS HLB 96-well μElution Plate, Waters). Peptides were subjected to an offline fractionation under high pH conditions (Hughes et al., 2014). The resulting 12 fractions were then analyzed by LC-MS/MS using a 2h gradient on an Orbitrap Fusion Lumos mass spectrometer (Thermo Scientific) as previously described (Sridharan et al., 2019). To this end, peptides were separated using an Ultimate 3000 nano RSLC system (Dionex) equipped with a trapping cartridge (Precolumn C18 PepMap100, 5 mm, 300 μm i.d., 5 μm, 100 Å) and an analytical column (Acclaim PepMap 100. 75 × 50 cm C18, 3 mm, 100 Å) connected to a nanospray-Flex ion source. The peptides were loaded onto the trap column at 30 μl per min using solvent A (0.1% formic acid) and eluted using a gradient from 2 to 40% Solvent B (0.1% formic acid in acetonitrile) over 2 h at 0.3 μl per min (all solvents were of LC-MS grade). The Orbitrap Fusion Lumos was operated in positive ion mode with a spray voltage of 2.4 kV and capillary temperature of 275°C. Full scan MS spectra with a mass range of 375–1500 m/z were acquired in profile mode using a resolution of 120,000 (maximum fill) time of 50 ms or a maximum of 4e5 ions (AGC) and a RF lens setting of 30%. Fragmentation was triggered for 3 s cycle time for peptide like features with charge states of 2–7 on the MS scan (data-dependent acquisition). Precursors were isolated using the quadrupole with a window of 0.7 m/z and fragmented with a normalized collision energy of 38. Fragment mass spectra were acquired in profile mode and a resolution of 30,000 in profile mode. Maximum fill time was set to 64 ms or an AGC target of 1e5 ions). The dynamic exclusion was set to 45 s.

Acquired data were analyzed using IsobarQuant (Franken et al., 2015) and Mascot V2.4 (Matrix Science) using a reverse UniProt FASTA *Drosophila melanogaster* database (UP000000803) (Bateman, 2019) including common contaminants. The following modifications were taken into account: Carbamidomethyl (C, fixed), TMT10plex (K, fixed), Acetyl (N-term, variable), Oxidation (M, variable) and TMT10plex (N-term, variable). The mass error tolerance for full scan MS spectra was set to 10 ppm and for MS/MS spectra to 0.02 Da. A maximum of 2 missed cleavages were allowed. A minimum of 2 unique peptides with a peptide length of at least seven amino acids and a false discovery rate below 0.01 were required on the peptide and protein level (Savitski et al., 2015).

Data analysis of mass spectrometry experiments

The protein.txt output files of IsobarQuant (Franken et al., 2015) were processed with the R programming language (ISBN 3-900051-07-0). To ensure a good data quality, only proteins that were quantified with at least 2 unique peptides (qupm column > = 2) were used for the following analysis. Furthermore, only proteins that have been identified in two out of three replicates were kept. The 'signal_sum' columns were cleaned for batch effects using the removeBatchEffect function of the limma package

(Ritchie et al., 2015). Then, data were normalized with the vsn package (Huber et al., 2002). A separate normalization was applied for control conditions, normal pull-down conditions and RNase treated pull-down conditions. Potential missing values were imputed with the impute function of the Msnbase package (Gatto and Lilley, 2012). Limma was used to test for differential abundance. Within this analysis, imputed values were given a weight of 5%. When testing for differential abundance between conditions of different normalization groups (e.g., normal pull-down versus control condition), adjusted p values from limma output were used as the false discovery rate (fdr). For all other tests, t-values from the limma output were used as an input to the fdrtool function of fdrtool (Strimmer, 2008) in order to calculate the fdr (qvalues were used). Proteins were classified as 'hit' with an fdr smaller 5% and a fold-change of at least 100% and as 'candidate' with an fdr smaller 20% and a fold-change of at least 50%. Hit and candidate proteins (tests: mutant versus WT, mutant_RNase versus WT_RNase, mutant versus WT / mutant_RNase versus WT_RNase) were clustered based on the Euclidean distance between normalized tmt reporter io signals (signal_sums) normalized by the WT or WT_RNase condition using the kmeans algorithm.

Sequence alignment and HMMER prediction

Sequences were aligned using the clustal omega tool (Madeira et al., 2019). Afterward the alignments were graphically modified using ESPript (Gouet et al., 2003). For the hidden markov model-based search, a sequence alignment only from the non-canonical CSDs was used as an input. The search was then run on the webserver (Potter et al., 2018).

Data presentation

Graphs were plotted using either Gnuplot 4 or Prism 5. Structure representations were done using PyMOL 2.3.2 (DeLano, 2002). Structures were superimposed using either the align algorithm for molecules with sequence identity, or the super algorithm for proteins, that differ in their protein sequence. The figures were generated using Inkscape version 0.92.3.

QUANTIFICATION AND STATISTICAL ANALYSIS

Chemical shift perturbations in Figure 2 were considered as significant for values bigger than the average plus the standard deviation of all measured shifts, which is indicated with a red line within each plot. The errors for the relaxation data in Figures 3C and 3E are derived from duplicate measurements of two relaxation delays for each experiment and further include the error of the exponential fit. These values are generated by the software (PINT; Ahlner et al., 2013; Niklasson et al., 2017). Except for Figure 4B, where only duplicates were measured, the mean of three individual experiments and the corresponding standard deviation is shown in Figures 4A and 4E, as indicated in Figure legend. These data were plotted and analyzed using GraphPad Prism 5. Hits for the RIP seq data (Figures 4C and 4D) were classified to be significant where the adjusted p value was lower than 0.1. In Figures 5D and 5E, proteins were classified as 'hit' with an fdr smaller 5% and a fold-change of at least 100% and as 'candidate' with an fdr smaller 20% and a fold-change of at least 50%. The analysis was done using RStudio.

Cell Reports, Volume *32*

Supplemental Information

Pseudo-RNA-Binding Domains Mediate

RNA Structure Specificity in Upstream of N-Ras

Nele Merret Hollmann, Pravin Kumar Ankush Jagtap, Pawel Masiewicz, Tanit Guitart, Bernd Simon, Jan Provaznik, Frank Stein, Per Haberkant, Lara Jayne Sweetapple, Laura Villacorta, Dylan Mooijman, Vladimir Benes, Mikhail M. Savitski, Fátima Gebauer, and Janosch Hennig

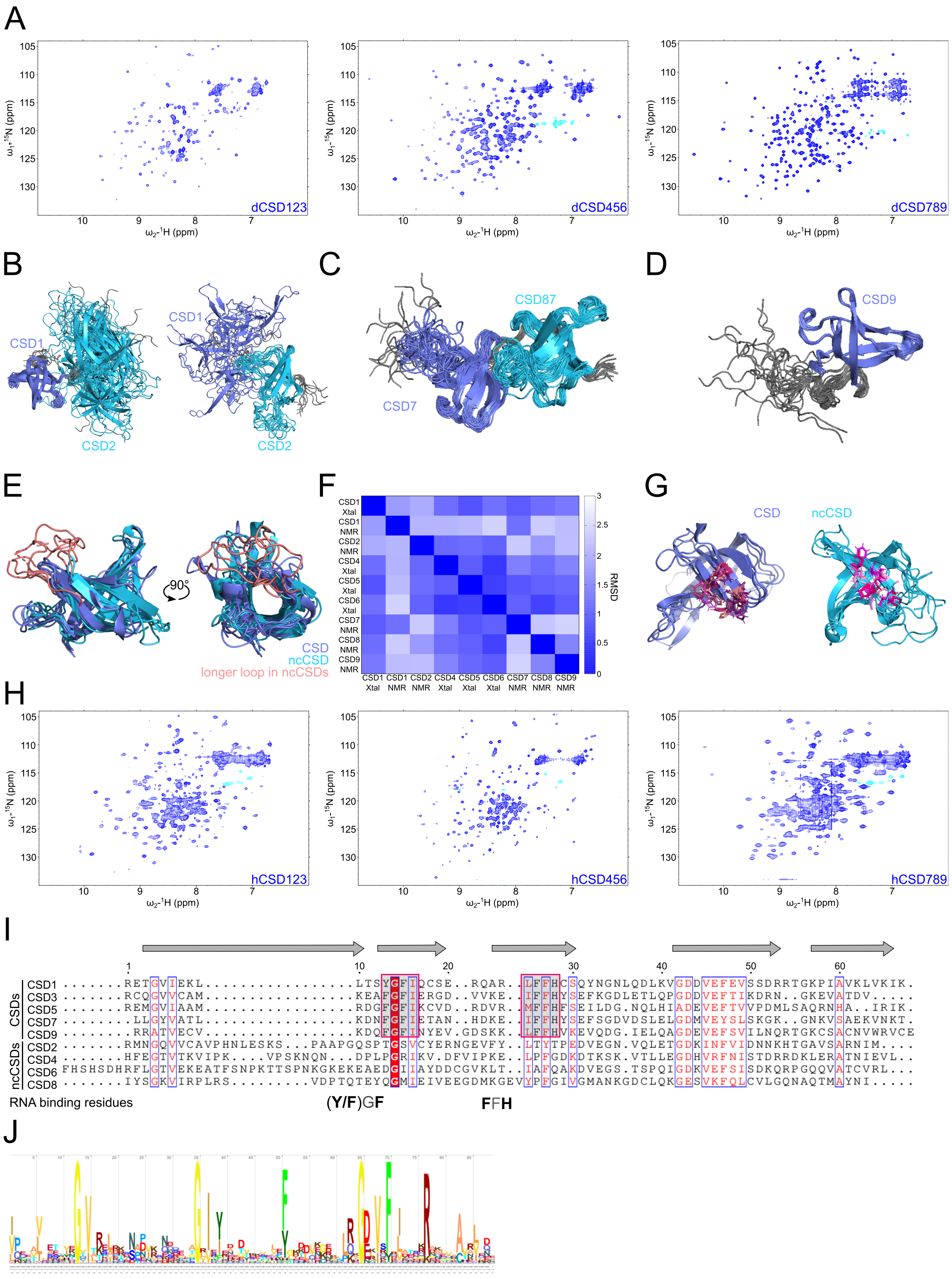

Supplementary Figure 1, Related to Figure 1: A: $^{1}H,^{15}N$-HSQC spectra of CSD123, CSD456 and CSD789 constructs. B: Ensemble of the 20 lowest energy NMR conformations of CSD12, superimposed on CSD1 (left) (residues: 197-237) and CSD2 (right) (residues: 269-274, 293-296 and 306-328) separately. C: Ensemble of the 20 lowest energy NMR conformations of CSD78 superimposed on secondary structure elements, excluding flexible loops and termini (residues 764-813, 824-834, 843-854, 867-873 and 879-920). D: Ensemble of the 20 lowest energy NMR conformations of CSD9 superimposed on the core CSD domain (residues 912-990). E: Superimposition of canonical CSDs 1, 5,

7 and 9 with ncCSDs 2, 4, 6 and 8. The additional loop that is present only in ncCSDs is highlighted in pink. F: Matrix of RMSD values from superimpositions of each single CSD of dUnr. For the comparison with CSD1 the crystal structure from PDB entry 4QQB was used. G: Superimposition of canonical CSDs 1, 5, 7 and 9 and ncCSDs 2,4,6 and 8 showing the RNA binding residues (F/Y-G-F and F-F-H) or equivalent residues pointing to the outside of the barrel (highlighted in shades of pink). H: ^{1}H,^{15}N-HSQC spectra of CSD123, CSD456 and CSD789 of human Unr exhibit peak dispersion indicative of folded proteins and similar to dUnr. I: Sequence alignment of canonical and ncCSDs of human Unr. The same or similar residues between all domains are colored, similar residue regions between the canonical CSDs are highlighted by boxes, which align with the two RNA binding regions (Y/FGF and FFH). The alignment has been done using Emboss Needle (Madeira et al., 2019) and ESPript (Robert and Gouet, 2014) has been used for illustration. J: Presentation of the sequence logo that was used for the hidden markov model search.

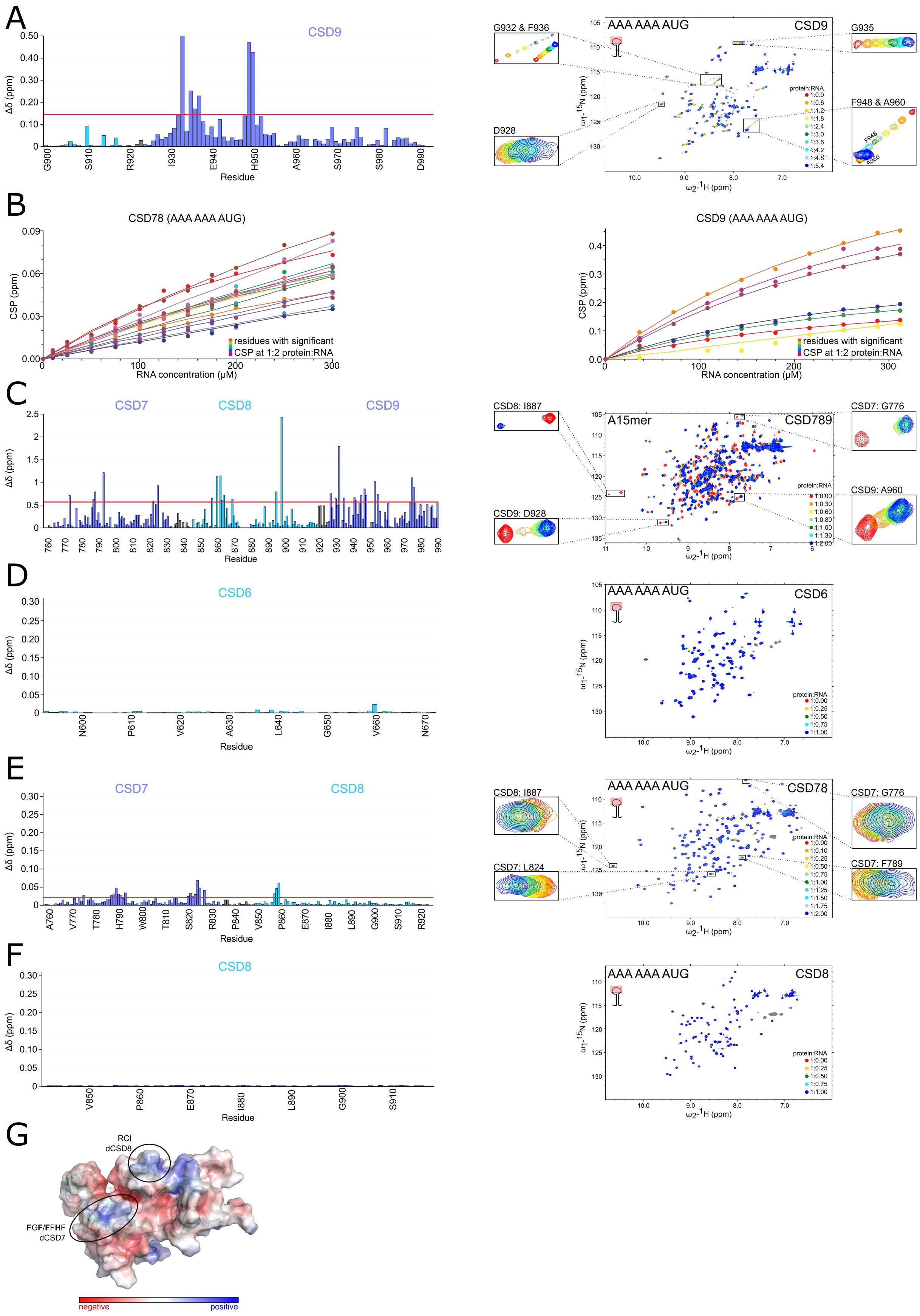

Supplementary Figure 2, Related to Figure 2: A: ^{1}H, ^{15}N-HSQC NMR titration of CSD9 (898-990) with a 9-mer RNA (AAA AAA AUG) zooming into regions, which show distinct shifts for some residues (right) and a histogram with chemical shift perturbation magnitude at end points per residue (left). B: The chemical shift perturbations and the corresponding fit for the different titration

concentrations for residues shifting significantly (CSP larger than the average plus standard deviation of all shifts) are shown. C: ^{1}H, ^{15}N-HSQC NMR titration of CSD789 (756-990) with an A15-mer RNA oligonucleotide zooming into regions, which show exemplary residues with distinct shifts (right) and a histogram with chemical shift perturbation magnitudes at the titration end points per residue (left). D: ^{1}H, ^{15}N-HSQC NMR titration of CSD6 (593-677) with a 9-mer RNA (AAA AAA AUG) (right) devoid of significant chemical shift perturbations, as shown also in the CSP plot (left). E: ^{1}H, ^{15}N-HSQC NMR titration of CSD78 (756-922) with a 9-mer RNA (AAA AAA AUG) zooming into regions, which show distinct shifts for some residues (right) and a histogram with chemical shift perturbations magnitude at end points per residue (left). F: ^{1}H, ^{15}N-HSQC NMR titration of CSD8 (841-922) with a 9-mer RNA (AAA AAA AUG) (right) devoid of significant chemical shift perturbations, as shown also in the CSP plot (left). G: Calculated surface potential for CSD78. Highlighted is the region of RNA binding residues of canonical CSD7 and the region of residues in ncCSD8, that show significant shifts after addition of RNA.

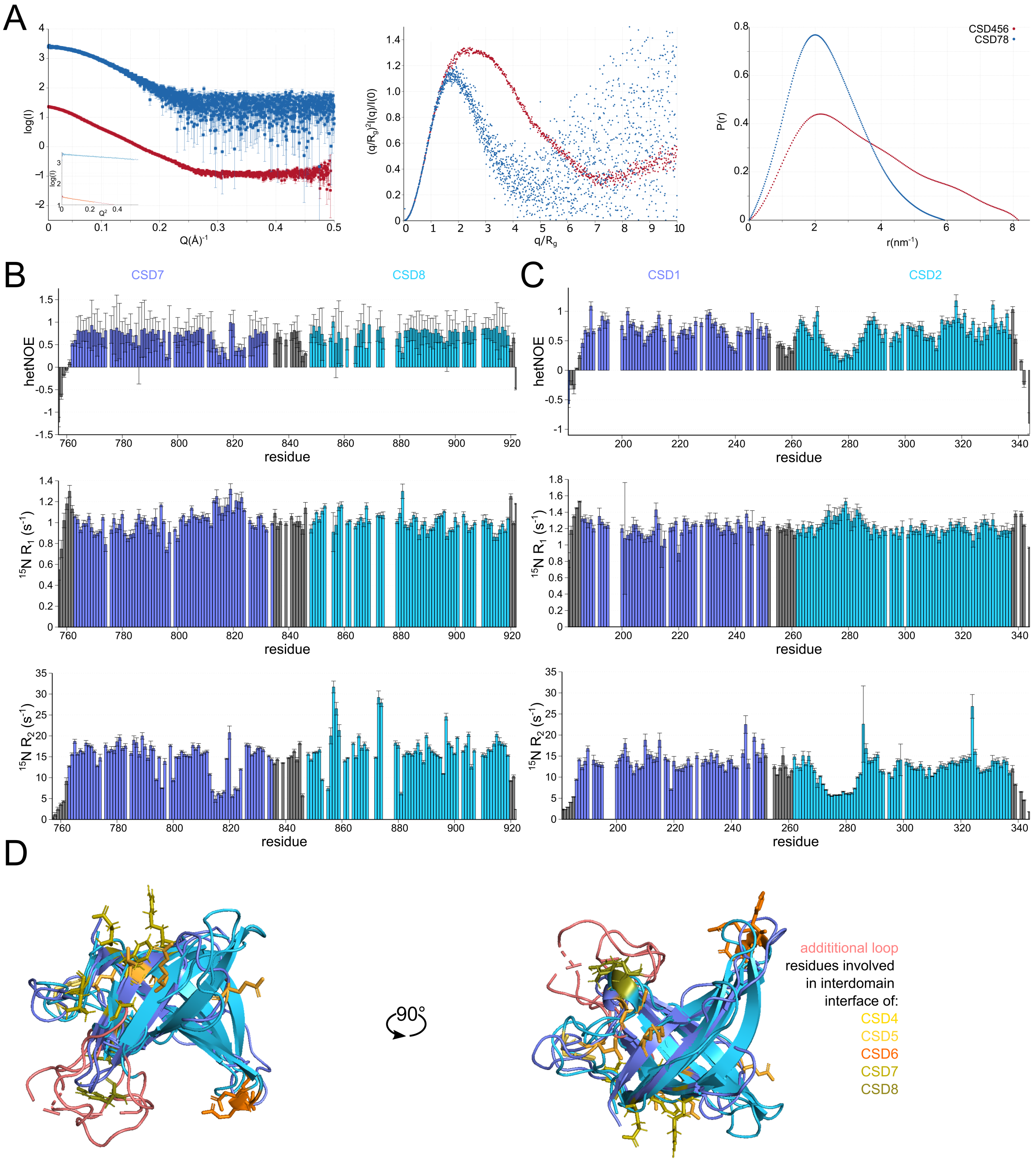

Supplementary Figure 3, Related to Figure 3: A left: *I*(*q*) *versus* *q* as log-linear plots with the inset showing the Guinier fits for $qR_g < 1.3$ indicating good data quality and no aggregation for the curves of CSD456 (red) and CSD78 (blue). Middle: Dimensionless Kratky plots indicate that proteins (CSD456: red; CSD78: blue) are mostly structured with low flexibility. Right: *P*(*r*) *versus* *r* profiles normalized to equal areas, showing the highest radius of gyration for CSD456 (red) and the lowest for CSD78 (blue). B and C: ^{15}N relaxation parameters of CSD78 (B) and CSD12 (C) showing the ^{1}H-^{15}N heteronuclear NOEs (hetNOE), the ^{15}N longitudinal relaxation rates (^{15}N R_1) and the ^{15}N transverse relaxation rates (^{15}N R_2). D: Superimposition of CSD5 and 7 and ncCSD4, 6 and 8, highlighting the additional loop that is present in the ncCSDs (pink) and the residues that are involved in formation of the interdomain interaction surface (shades from yellow to green).

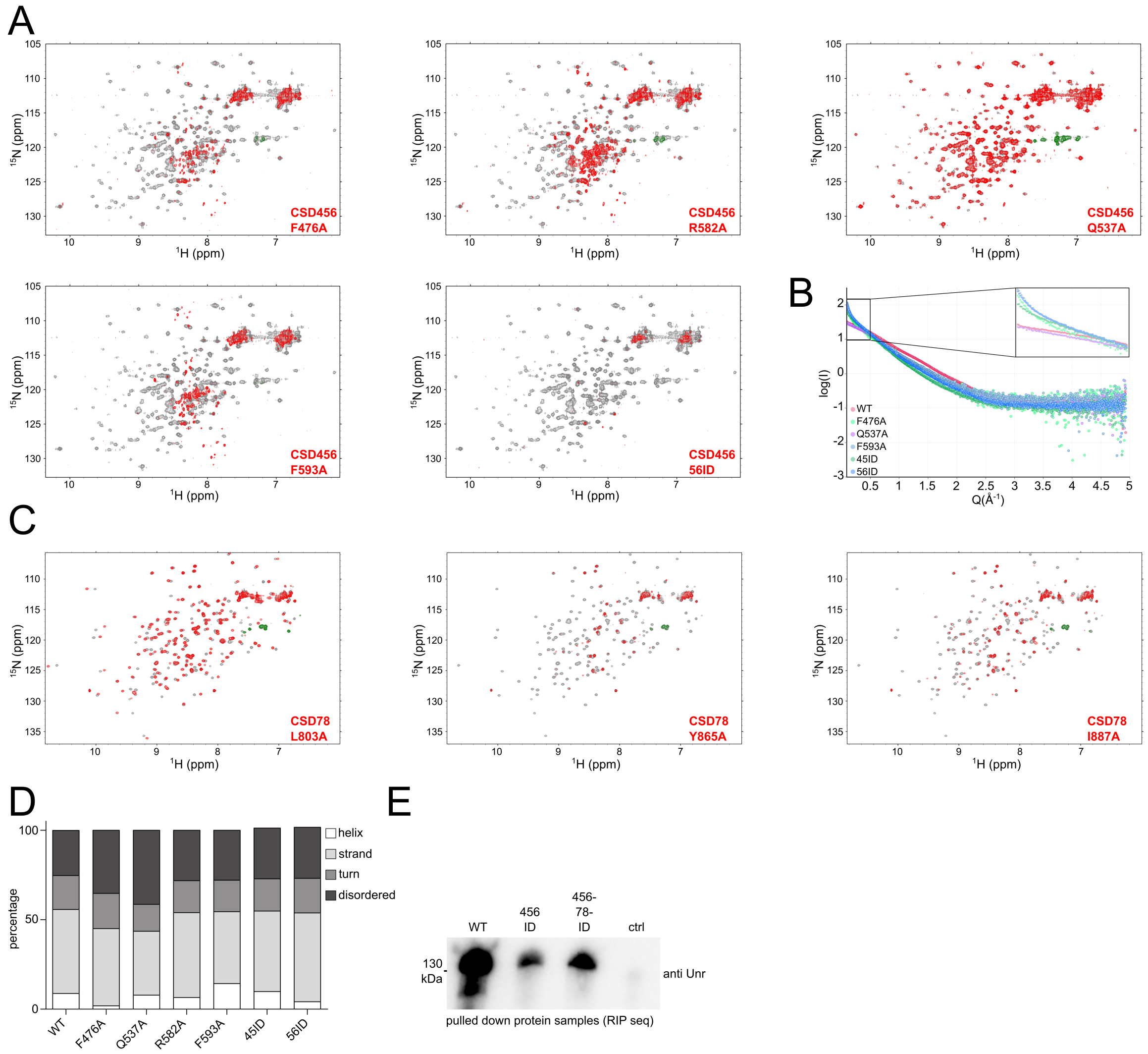

Supplementary Figure 4, Related to Figure 4: A: ^{1}H,^{15}N-HSQC spectra of CSD456 wild type (grey) overlaid with the spectra of CSD456 mutants (red) showing aggregation of most of the mutants compared to the wild type. The proteins had a concentration of 30 µM. B: SAXS curve of CSD456 wild type construct and the different mutants measured at the same concentration (1 mg/ml). Again, except for the Q538A mutant aggregation could be measured. C: ^{1}H,^{15}N-HSQCs of CSD78 wild type (grey) overlaid with the different spectra of CSD78 mutants (red). The L803A mutant shows CSPs compared to the wild type sample, whereas for the other two mutants aggregation could be observed The proteins had a concentration of 80 µM. D: Prediction of different secondary structure elements (helix, strand, turn and disordered) from CD curves of Unr full-length wt and different interdomain mutants, showing no difference between the wild type and mutant samples. E Western blot showing the efficiency of the Unr pull-down against the V5 tagged wild type and the two interdomain mutants of the samples (456ID, 456-78-ID) used for RIP seq. No protein was detected in the control sample, which was transfected with an empty vector. Unr antibody against an N-terminal part of the protein was used.

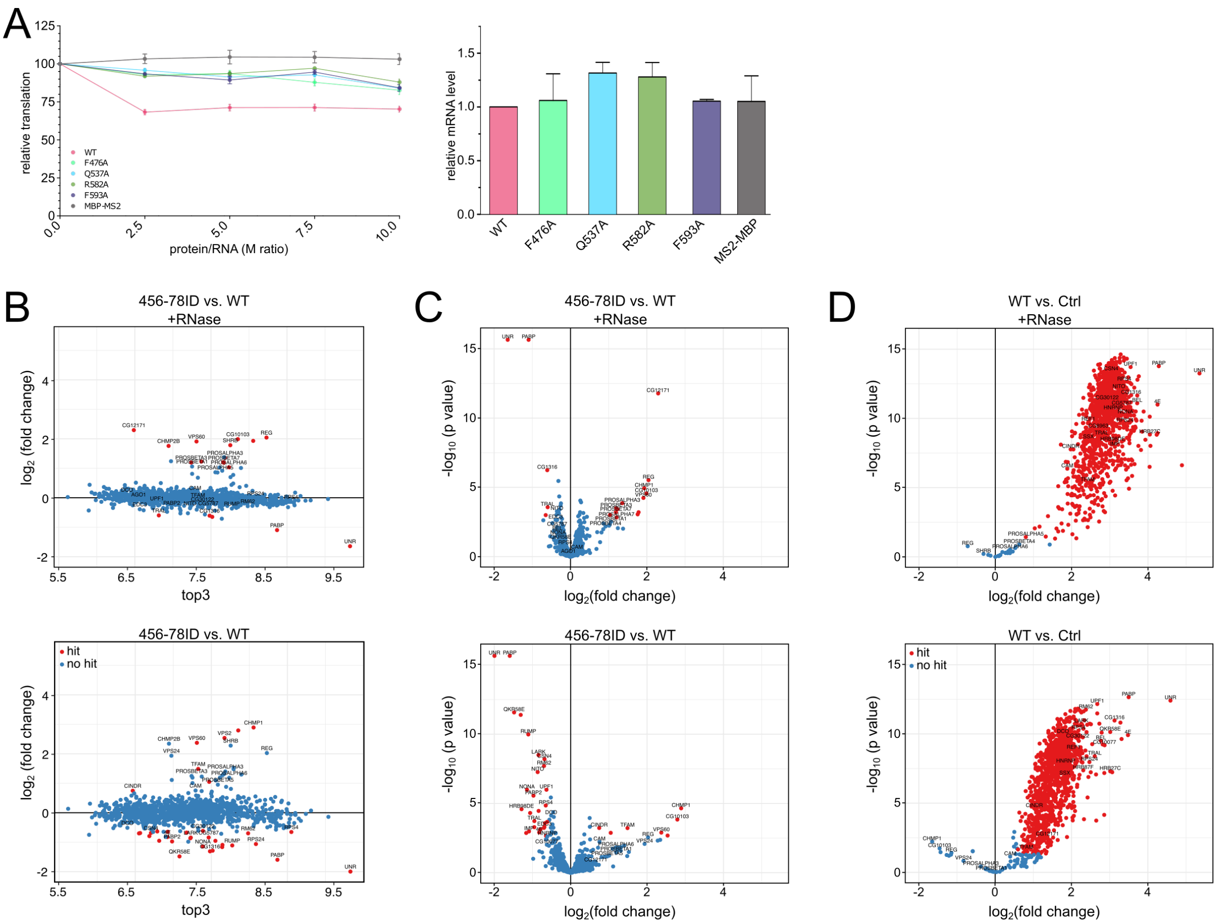

Supplementary Figure 5, Related to Figure 5: A left: Relative *in vitro* translation of the Firefly reporter gene over the internal control Renilla after adding different amounts of single dUnr mutants compared to wild type dUnr. N=3. The wild type protein shows a higher translation repression rate than the mutants. The control (MBP-MS2) doesn't show any repression. Right: Relative mRNA levels of the ratio of Renilla and Firefly after in vitro translation experiments, measured by qPCR. N=3 from 2.5 excess of protein over RNA, indicating, that the observed difference in signal is not due to different mRNA levels.. B: Scatter plot showing the top3 value (average abundance of a protein in the mass spectrometry run) versus the log2(fold change) of interdomain mutant vs WT for RNase treated (top) and non-treated (bottom) conditions. N=3. C/D: Volcano plots showing the difference of protein targets of the IP-MS between the WT sample and the 456-78 interdomain mutant (C) or the control (D) with (upper graph) and without RNase treatment (lower graph). The log_2(fold change) is plotted against the negative log_{10}(p value). Differentially regulated proteins are colored in red. D: Due to the low background level of the control sample, almost all proteins were classified as differentially regulated. N=3

Table S1, Related to Figure 1. List of soluble and unsoluble protein constructs tested for *Drosophila* Unr.

boundaries	old nomenclature	new nomenclature	tag
2-130	N-temrinus + q-rich	N-temrinus + q-rich	His
2-183	N-temrinus + q-rich	N-temrinus + q-rich	His
11-130	N-temrinus + q-rich	N-temrinus + q-rich	His
11-183	N-temrinus + q-rich	N-temrinus + q-rich	His
176-414	CSD1/2	CSD1/2/3	His
176-669	CSD123	CSD1-6	His
176-677	CSD123	CSD1-6	His
186-252	CSD1	CSD1	His-Trx
186-324	CSD1	CSD12	His
186-339	CSD1	CSD12	His
186-341	CSD1	CSD12	His
186-344	CSD1	CSD12	His
186-414	CSD1/2	CSD1/2/3	His
186-990	CSD1-5	CSD1-9	His
345-515	CSD2	CSD34	His
345-586	CSD35	CSD345	His
422-515		CSD4	His
422-586	CSD3	CSD4/5	His
422-677	CSD3	CSD456	His
422-853	CSD3/4	CSD4-7	His
428-990	CSD345	CSD4-9	His
516-581	CSD3	CSD5	His
516-669	CSD3	CSD5/6	His
593-677		CSD6	His
593-757		CSD6 + q-rich	
593-922	CSD4	CSD678	His
678-922	CSD4	q-rich + CSD78	
757--922	CSD4	CSD78	His
757-853	CSD4	CSD7	His
757-856	CSD4	CSD7	His
757-991	CSD4/5	CSD7/8/9	His
856-991	CSD5	CSD8/9	His
899-991	CSD5	CSD9	His
2-52	N-terminus	N-terminus	His
2-61	N-terminus	N-terminus	His
11-52	N-terminus	N-terminus	His
11-61	N-terminus	N-terminus	His
47-130	N-temrinus + q-rich	N-temrinus + q-rich	His
47-183	N-temrinus + q-rich	N-temrinus + q-rich	His
54-130	q-rich	q-rich	His

soluble - not used in publication
soluble - used in publication
not soluble

54-183	q-rich	q-rich	His
62-130	q-rich	q-rich	His
62-183	q-rich	q-rich	His
186-1017	CSD1-5	CSD1-9	His
186-669	CSD123	CSD1-6	His
186-677	CSD123	CSD1-6	His
285-424	CSD2	CSD2/3	His
286-502	CSD2	CSD34	His
292-414	CSD2	CSD23	His
293-581	CSD35	CSD345	His
293-586	CSD35	CSD345	His
293-642	CSD35	CSD345	His
293-669	CSD35	CSD3456	His
293-677	CSD35	CSD3456	His
298-502	CSD2	CSD34	His
298-515	CSD2	CSD34	His
345-414	CSD2	CSD3	His
345-414	CSD2	CSD3	His-MBP
345-502	CSD2	CSD34	His
345-581	CSD35	CSD345	His
345-643	CSD35	CSD345	His
345-669	CSD35	CSD3456	His
345-677	CSD35	CSD3456	His
422-1017	CSD345	CSD4-9	His
422-502		CSD4	His
422-581	CSD3	CSD4/5	His
422-642	CSD3	CSD45	His
422-832	CSD34	CSD4-7	His
422-835	CSD34	CSD4-7	His
422-856	CSD34	CSD4-7	His
422-875	CSD34	CSD4-7 (8half)	His
422-910	CSD34	CSD4-7 (8half)	His
422-910	CSD34	CSD4-8	His
422-990	CSD345	CSD4-9	His
428-1017	CSD345	CSD4-9	His
428-502		CSD4	His
428-515		CSD4	His
428-581	CSD3	CSD4/5	His
428-596	CSD3	CSD4/5	His
446-642	CSD3	CSD56	His
446-832	CSD34	CSD4-7	His
446-835	CSD34	CSD4-7	His
446-853	CSD34	CSD4-7	His
446-856	CSD34	CSD4-7	His

446-875	CSD34	CSD4-7 (8half)	His
446-910	CSD34	CSD4-8	His
516-586	CSD3	CSD5	His
516-586	CSD3	CSD5	His-MBP
516-586	CSD3	CSD5	His
516-677	CSD3	CSD5/6	His
516-832	CSD34	CSD5-7	His
516-835	CSD34	CSD5-7	His
516-853	CSD34	CSD5-7	His
516-856	CSD34	CSD5-7	His
516-875	CSD34	CSD5-7 (8half)	His
516-910	CSD34	CSD5-8	His
593-669		CSD6	His
678-757	q-rich	q-rich	His
678-703	q-rich	q-rich	His
703-757	q-rich	q-rich	His
757-1017	CSD4/5	CSD7/8/9	His
757-875	CSD4	CSD7	His
757-890	CSD4	CSD78	His
757-900	CSD4	CSD78	His
757-911	CSD4	CSD78	His
763-832	CSD4	CSD7	His
763-832	CSD4	CSD7	His-MBP
763-910	CSD4	CSD7	His
856-1017	CSD5	CSD8/9	His
899-1017	CSD5	CSD9	His
910-1017	CSD5	CSD9	His
911-990	CSD5	CSD9	His
923-990	CSD5	CSD9	His
345-414	CSD2	CSD3	His-Trx
516-586	CSD3	CSD5	His-Trx
763-832	CSD4	CSD7	His-Trx
923-990	CSD5	CSD9	His-Trx

Table S2, Related to Figure 1. Statistics of the NMR structure calculation of *Drosophila* Unr CSD12 bound to SL6 apical RNA, CSD78 and CSD9 and data collection and refinement statistics of the crystal structure of *Drosophila* Unr CSD456. Statistics for the highest-resolution shell are shown in parentheses.

NMR structure statistics			
	**dCSD12+SL6 apical**	**dCSD78**	**dCSD9**
	**Experimental restraints**		
Total NOEs	3911	4530	3712
distance restraints	1788	2323	1831
Short range (|i – j| ≤ 1)	1005	1242	544
Medium range (|i – j| < 5)	124	211	283
Long range (|i – j| > 5)	659	870	1004
Dihedral restraints (ϕ/ψ)	224	167	93
	**Structural Quality**		
Coordinate precision (Å)			
Backbone (N, Cα, C′)	0.40 (CSD1), 0.53 (CSD2) (a)	0.39 (CSD7), 0.39 (CSD8) (b)	0.38 (c)
Heavy atoms	0.99 (CSD1), 1.05 (CSD2) (a)	0.84 (CSD7), 0.86 (CSD8) (b)	0.80 (c)
Restraint RMSD			
Distance restraints, Å	0.02 +/- 0.0019	0.0196 +/- 0.002	0.0253 +/- 0.003
Dihedral restraints, °	0.666 +/- 0.104	1.437 +/- 0.101	0.565 +/- 0.105
Deviation from idealized geometry			
Bond lengths, Å	0.0038 +/- 0.00008	0.0036 +/- 0.00009	0.00434 +/- 0.00013
Bond angles, °	0.504 +/- 0.01	0.491 +/- 0.011	0.542 +/- 0.022
	**Whatcheck analysis**		

First generation packing	-3.271 +/- 0.228	-2.751 +/- 0.161	-1.912 +/- 0.191
Second-generation packing	-2.491 +/- 0.274	-2.402 +/- 0.239	-1.560 +/- 0.284
Ramachandran plot appearance	-3.806 +/- 0.298	-3.843 +/- 0.329	-4.442 +/- 0.480
χ-1/χ-2 rotamer normality	-3.806 +/- 0.532	-3.915 +/- 0.473	-5.416 +/- 0.431
Backbone confirmation	-1.261 +/- 0.534	-2.329 +/- 0.537	-1.326 +/- 0.376
	**Ramachandran analysis, %**		
Favored regions	80.9	76.7	80.5
Allowed regions	16.7	20.9	18.9
Generously allowed	1.9	1.7	0.6
Disallowed	0.4	0.7	0.0

a	For residues using 197-237 (CSD1) and 269-274, 293-296 and 306-328 (CSD2)
b	For residues using 764-813 and 824-834 (CSD7) and 843-854, 867-873 and 879-920 for (CSD8)
c	For residues using 912-990

**Crystallography statistics CSD456**	
Wavelength	1.005 Å
Resolution range	77.84 - 2.02 (2.092 - 2.02)
Space group	P 62
Unit cell	89.88 89.88 58.75 90 90 120
Total reflections	113928 (6419)
Unique reflections	34128 (2534)
Multiplicity	3.3 (2.5)
Completeness (%)	98.00 (87.3)
Mean I/sigma(I)	7.8 (1.0)
Wilson B-factor	39.76
R-merge	0.073 (0.78)
R-meas	0.087 (0.95)
R-pim	0.046 (0.54)
CC1/2	0.99 (0.57)
Reflections used in refinement	32428 (2409)
Reflections used for R-free	1698 (124)

R-work	0.22 (0.32)
R-free	0.25 (0.32)
Number of non-hydrogen atoms	2062
macromolecules	1842
ligands	7
solvent	213
Protein residues	238
RMS(bonds)	0.002
RMS(angles)	0.38
Ramachandran favored (%)	96.93
Ramachandran allowed (%)	2.63
Ramachandran outliers (%)	0.44
Rotamer outliers (%)	0.00
Average B-factor	47.73
macromolecules	47.83
ligands	51.27
solvent	46.76

Table S3, Related to Figure 3. Statistics of the SAXS data and processing statistics of *Drosophila* Unr CSD456 and CSD78.

	**dCSD456**	**dCSD78**
(a) Sample Details		
Organism	*E. coli* BL2 (DE3)	*E. coli* BL2 (DE3)
Source	*this work*	*this work*
Uniprot sequence ID	Q9VSK3	Q9VSK3
Description	UNR E422-H677, with TEV-cleaved N-terminal His6-tag	UNR A756-K922, with TEV-cleaved N-terminal His6-tag
Molecular mass M from chemical composition (Da)	29.532	18.597
loading concentration (mg/ml)	1.16 and 4.65 mg/ml	0.8 mg/ml
injection volume (ul)	30	30
concentration (uM)	40/160	45
Solvent composition and source	20 mM Hepes/NaOH pH 7.5, 150 mM NaCl and 1 mM DTT	
(b) SAS data collection parameter		
Source and instrument	Grenoble ESRF BM29 with Dectris Pilatus 1M	Hamburg PETRA-III P12 with Dectris Pilatus 6M (REF)
Wavelength (A)	0.9919	1.24
Sample-detector distance (m)	2,867	3.0
q-measurement range (nm-1)	0.0355-4.9391	0.0224-7.3176
Radiation daage monitoring	frame-by-frame comparison	
Exposure time (s) & number	1.0x10	0.195x20
Sample configuration	sample chamger with flow through capillary measurement	
Sample temperature (℃)	20	25
(c) Software employed for SAS data reduction, analysis and interprtation		
SAXS data processing	I(q) vs. q using Bsx cube, solvent subtraction and curve merging using PRIMUSqt from ATSAS (Franke et al., 2017)	
Basic analyses: Guinier, P(r), Vp	PRIMUSqt from ATSAS 2.7.1 (Franke et al., 2017)	

Atomic structure modelling	CRYSOL 2.8.2 from PRIMUSqt in ATSAS 2.8 (Svergun et al., 1995)	
Molecular graphics	--	--
(d) Structural parameters		
	Guinier analysis	
I(0) (raw)	22.8+/-0.06	25.4+/-0.05
R_g (Å)	26.7+/-0.1	18.1+/-0.01
qR_g max (q_{min} = 0.0066 $Å^{-1}$)	1.28	1.3
Coefficient of correlation, R^2	0.97	0.74
	P(r) Analysis from AUTOGNOM	
I(0) (cm-1)	22.8	25.43
R_g (Å)	22.61	18.2
d_{max} (Å)	81.5	59.1
q range ($Å^{-1}$)	0.102-3.00	0.089-4.427
χ^2 (total estimate from *GNOM*)	0.67	0.89
Porod volume ($Å^{-3}$) (ratio V_P/calculated *M)*	37490	27610
(f) Atomistic modelling		
Method	CRYSOL (Svergun et al., 1995)	
Crystal stucture	UNR CSD456 (6Y6E)	UNR CSD78 (6Y4H)
CRYSOL	Constant subtraction allowed	
χ^2	1,026	1,218
Predicted R_g (Å)	26.22	18.09
Vol (Å), Ra (Å), Dro (e $Å^{-3}$)	25504, 1.4, 0.07	17716, 1.8, 0.000

Table S5, Related to STAR Methods. Sequences for DNA oligonucleotides.

REAGENT or RESOURCE	SOURCE	IDENTIFIER
RNAi targeting Unr forward primer: TAATACGACTCACTATAGGGATTGCTGAAGAAGTTGTATAAGCAA	Militti et al., 2014	N/A
RNAi targeting Unr reverse primer: TAATACGACTCACTATAGGGACACTTGGATTTCAATTTCGTTTTGC	Militti et al., 2014	N/A
RNAi targeting GFP forward primer: TAATACGACTCACTATAGGGCCTGAAGTTCATCTGCACCA	Militti et al., 2014	N/A
RNAi targeting GFP reverse primer: TAATACGACTCACTATAGGGTGAAATTTGTGATGCTATTGCTTT	Militti et al., 2014	N/A
forward qPCR primer against firefly luciferase used in *in vitro* translation assay: TTGTTTCCAAAAAGGGGTTG	Graindorge et al., 2013	N/A
reverse qPCR primer against firefly luciferase used in *in vitro* translation assay: CATCGACTGAAATCCCTGGT	Graindorge et al., 2013	N/A
forward qPCR primer against renilla luciferase used in *in vitro* translation assay: TATTGCTTTGATCTTATCTTGATGC	This paper	N/A
reverse qPCR primer against renilla luciferase used in *in vitro* translation assay: ACAAATATCTTACTGCATGGTTTG	This paper	N/A
forward qPCR primer against β-galactosidase used in cell assay: AACGTCGTGACTGGGAAAAC	Graindorge et al., 2013	N/A
reverse qPCR primer against β-galactosidase used in cell assay: GGCCTCTTCGCTATTACGC	Graindorge et al., 2013	N/A
forward qPCR primer against renilla luciferase used in cell assay: ACAAGTACCTCACCGCTTGG	Graindorge et al., 2013	N/A
reverse qPCR primer renilla luciferase used in cell assay: GACACTCTCAGCATGGACGA	Graindorge et al., 2013	N/A
